# Supplementary material for: The influence of a manipulation of threat on experimentally-induced secondary hyperalgesia
Source: PeerJ. 2022 Jun 20;10:e13512. doi: 10.7717/peerj.13512 (PMC9220919; doi:10.7717/peerj.13512)
Supplement: Supplemental Information 1 [file peerj-10-13512-s001.docx]

### Piloting procedure

An extensive piloting procedure was conducted to ensure the effectiveness of the threat manipulation procedure. We predicted that it may be difficult to make participants feel threatened due to the controlled environment associated with experimental research paradigms. After each piloting procedures, we conducted open interviews with participants to obtain feedback on the effectiveness of the threat manipulation. Below is a summary of the piloting process including the objectives of each stage, themes arising from the post-procedure interviews, outcomes of each stage, and amendments made to the threat manipulation (Tables 1, 2 and 3). Originally included in Bedwell (2020).

#### Piloting: stage one

Table 1 Outlining the objectives, outcome, reflections, and strategies used to overcome the challenges of piloting stage one

| Number of participants:  n = 10 | **Objective:** To assess the effectiveness of a visual threat manipulation consisting of images of injured (assumed to be threatening - experimental) and uninjured (control) upper limbs.  **Themes arising from post-procedure interviews:**   - Difficulty in obtaining images of arms belonging to a variety of skin colours – potentially influencing the relatability of the threat manipulation to participants with difference skin colours to those in the images. - Participants were not concerned about potential tissue damage to their own arm while viewing images of other people’s injured arms. - The threat manipulation was not personal enough and therefore did not evoke a sense of threat in the participants.   **Outcome:** The visual threat manipulation was not effective in producing a plausible threat of tissue damage in the participants.  **Amendments to the threat manipulation:**  Goal: to make the threat more personal   - the threat manipulation was changed to be an audio voice recording explaining the potential risks of the HFS procedure. - Possessive pronouns, such as ‘*your* skin’, were used to make the threat more personal. |
| --- | --- |

####

#### Piloting: stage two

Table 2 Outlining the objectives, outcome, reflections, and strategies used to overcome the challenges of piloting stage two

| Number of participants:  n = 9 | **Objective:** To assess the effectiveness of an audio threat manipulation consisting of threatening (experimental) and non-threatening (control) information about the HFS procedure. This is a similar threat manipulation to that used by Van Damme et al. (2008) in which participants were given either threatening or neutral information about a cold pressor test prior to immersing their hand into the bucket of cold water. Using self-reported anxiety and catastrophic thoughts about the cold pressor test as the manipulation check, Van Damme et al. (2008) concluded that this threat manipulation was effective.  **Themes arising from post-procedure interviews:**   - Participants trusted that the HFS procedure would not be harmful and assumed the researchers were being excessively cautious. - Participants who were socially acquainted with the researchers were particularly trusting and did not find the threat manipulation compelling. - Again, the threat manipulation was not personal enough to evoke a personal threat of tissue damage.   **Outcome:** The audio threat manipulation was not effective in producing a plausible threat of tissue damage in the participants.  **Amendments to the threat manipulation:**  Goal: To make the threat manipulation even more personal.   - The threat manipulation was further adapted to a sham skin examination procedure. In this procedure the integrity of participants’ skin around the HFS site was ‘assessed’ (sham). - Where possible, this author avoiding recruiting people who were socially acquainted with the researchers involved in this project. |
| --- | --- |

####

#### Piloting: stage three

Table 3 Outlining the objectives, outcome, reflections, and strategies used to overcome the challenges of piloting stage three

| Number of participants:  n = 6 | **Objective:** To assess the effectiveness of a sham skin examination procedure for the threat manipulation. This is a similar threat manipulation to that used by Wiech et al. (2010). In that study, a sham skin integrity test was conducted in which areas of the skin at the site intended to receive laser stimulation were initially categorised as either “fully approved” for the delivery of laser stimulation or “approved with reservations”. Participants were informed that the sites that were “fully approved” were strong enough to withstand the laser stimulus. But the site “approved with reservations” needed close monitoring to prevent tissue damage during the laser stimulation. Using pain ratings from the laser stimulus as a manipulation check,  Wiech et al. (2010) concluded this threat manipulation to be effective in their cohort.  **Themes arising from post-procedure interviews:**   - The researcher conducting the experiment was able to figure out which arm was receiving the HFS under a condition of threat and which was not, thus rendering them unblinded. During this piloting stage, participants were randomised into two groups: Group 1 received the HFS under a condition of threat on the *right* arm and Group 2 received HFS under a condition of threat on the *left* arm. The researcher conducting the experiment knew whether participants were in Group 1 or Group 2 but was naïve to site allocation for each group, i.e. they were not informed which arm received the HFS under a condition of threat in each group. However, after conducting informal interviews with the researcher, it became apparent that the researcher was able to fairly accurately estimate site allocation, based on participants’ facial expressions during the HFS procedure and verbal feedback from participants after the procedure. Therefore, rendering them unblinded for the subsequent participant. - This threat manipulation was conducted on a very small cohort. Therefore, it was underpowered to perform statistical comparisons to determine whether there was a significant difference in anxiety ratings and/or threat ratings between site allocation (i.e. which arm received the HFS under a condition of threat). However, based on informal feedback discussions with participants, this threat manipulation was deemed to be the most compelling.   **Outcome:**   - Threat manipulation deemed compelling, based on informal feedback discussions from participants.   **Amendments to the threat manipulation:**  Goal: ensure blinding of the researcher conducting the experiment.  To achieve this goal, three researchers were involved in running the procedure to ensure blinding of the researcher conducting the outcome measurements. The first, independent researcher randomly allocated participants to site allocation, i.e. which arm would receive the HFS under a condition of threat. A second, independent assistant designated the site allocation in the Affect5 programme. The third researcher conducted the full HFS procedure and outcome measurements without being informed of the site allocation. Additionally, the third researcher (conducting the full HFS procedure and outcome measurements) completed a blinding assessment where they were asked two questions: 1) “Which arm do you think received the HFS under a condition of threat?” and 2) “How confident are you in your answer to the previous questions?” (answered on a on a five-point Likert scale consisting of “not at all confident”, “not confident”, “neutral”, “confident”, “extremely confident”).  On completion of piloting of the threat manipulation, the procedures for the full study was initiated. |
| --- | --- |

BEDWELL, G. J. 2020. *The effect of stimulus threat on experimentally induced secondary hyperalgesia.* Faculty of Health Sciences.
