# Supplementary figures and images for: The influence of a manipulation of threat on experimentally-induced secondary hyperalgesia
Source: PeerJ. 2022 Jun 20;10:e13512. doi: 10.7717/peerj.13512 (PMC9220919; doi:10.7717/peerj.13512)

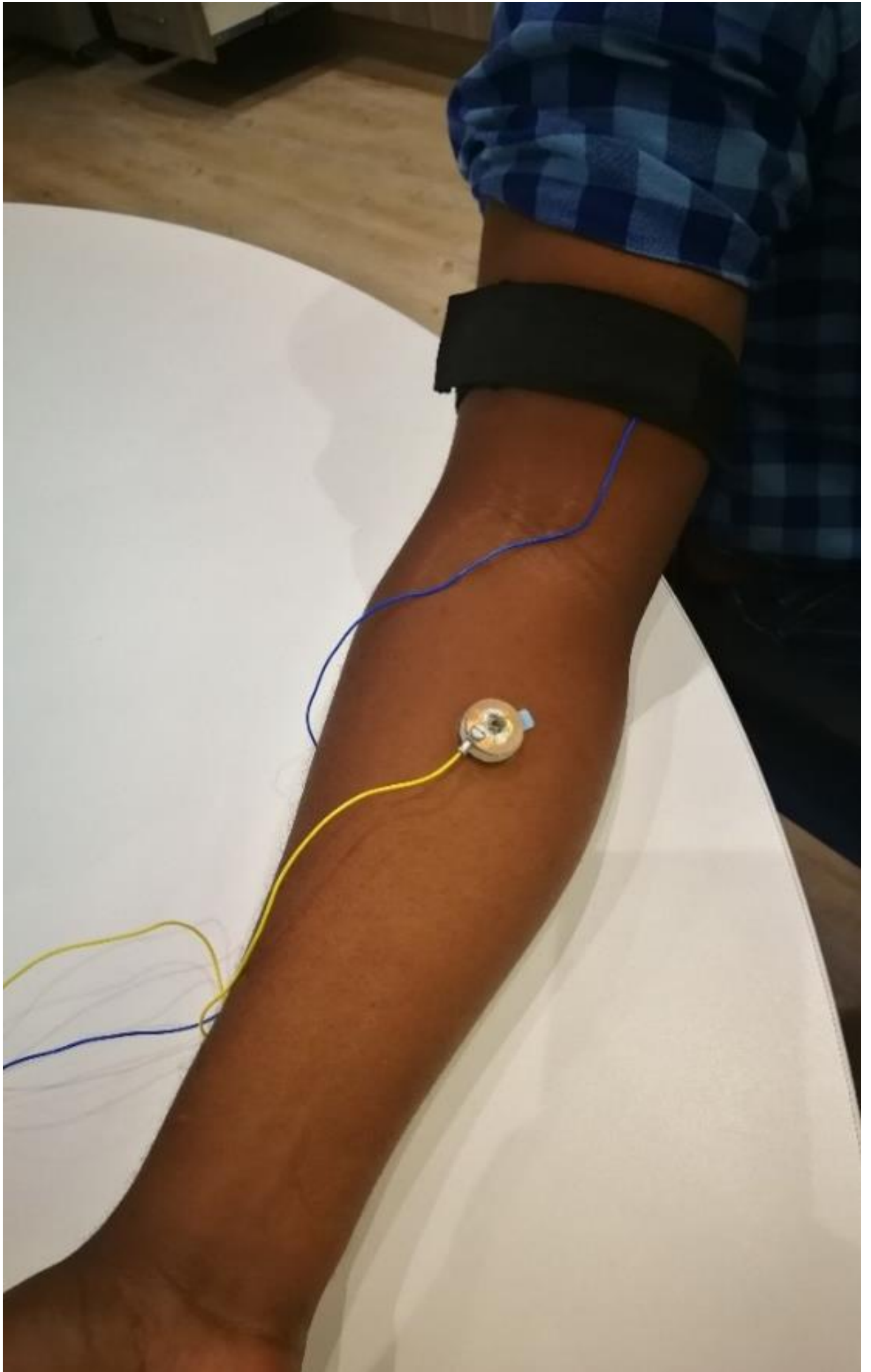

Supplement: Supplemental Information 2 — Cathode is secured with double-sided tape to the participant’s anterior forearm and anode secured with Velcro to participant’s mid-upper arm. Photo credit: Gillian J Bedwell. [file peerj-10-13512-s002.pdf]

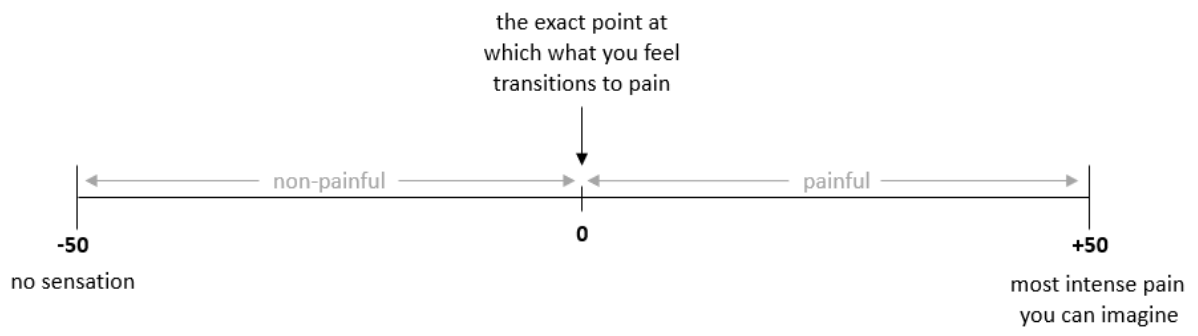

Supplement: Supplemental Information 3 [file peerj-10-13512-s003.pdf]

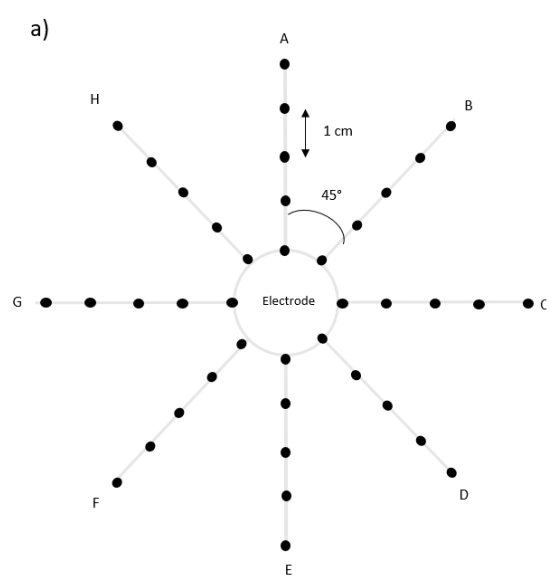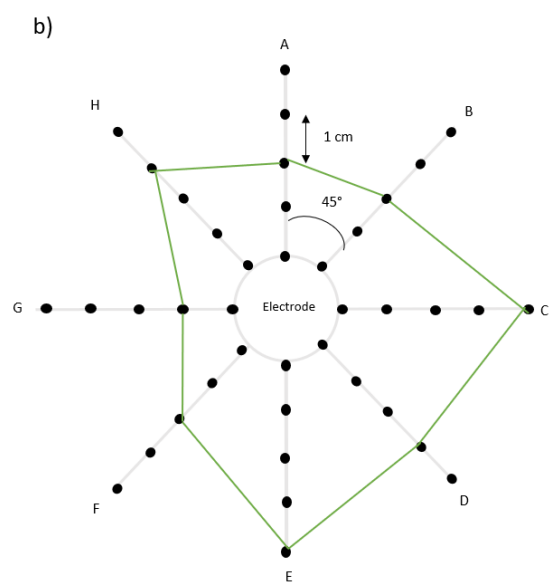

Supplement: Supplemental Information 4 — (a) An image of the eight radial lines that originate at the centre of the site of the electrode. Each line is at a 45° angle to its neighbours, and each dots are 1 cm apart. (b) An example of a mapped area of secondary hyperalgesia. The green lines indicate the border of the area of secondary hyperalgesia. [file peerj-10-13512-s004.pdf]
