# Supplementary material for: The influence of a manipulation of threat on experimentally-induced secondary hyperalgesia
Source: PeerJ. 2022 Jun 20;10:e13512. doi: 10.7717/peerj.13512 (PMC9220919; doi:10.7717/peerj.13512)
Supplement: Supplemental Information 5 [file peerj-10-13512-s005.pdf]

The influence of a manipulation of threat on experimentally induced secondary hyperalgesia

Gillian J Bedwell and Victoria J Madden

31 Mar 2022

Contents

Demographic data **2**

Manipulation check **2**

Primary outcome **2**

Secondary outcome **2**

Questionnaires **3**

Childhood and recent trauma scores **3**

Post-hoc analyses **3**

Order effect **3**

Exploratory outcomes **3**

Static light touch 3

Dynamic light touch 3

Electrical stimulation 3

Import data **3**

Demographic data 4

Manipulation checks **5**

HFS ratings 5

Anxiety ratings 10

Threat ratings 13

Blinding assessment **18**

Primary outcome: magnitude of secondary hyperalgesia **21**

Magnitude of secondary hyperalgesia: Assessment of model fit **29**

Secondary outcome: surface area of secondary hyperalgesia **31**

Surface area of secondary hyperalgesia: Assessment of model fit **35**

Childhood and recent trauma scores **38**

Order effect **44**

Exploratory outcomes: **47**

Static light touch 47

Dynamic light touch 51

Electrical stimulation 55

Session information **59**

Demographic data

- Determine the median (range) age in years.
- Determine the number of male and female participants.
- Determine the mean (SD) intensity used from the HFS induction.

Manipulation check

- Describe and compare HFS ratings between arms.
- Describe and compare participant anxiety self-ratings between arms.
- Describe and compare participant threat self-ratings between arms.
- Assess whether blinding of the assessor who conducted the experiment and sensory testing was upheld.

Primary outcome

Determine the magnitude of secondary hyperalgesia (SH) on each arm, and compare between arms.

Secondary outcome

Determine the surface area (SA) of secondary hyperalgesia (SH) on each arm, and compare between arms.

Questionnaires

- Describe scores on the Childhood Trauma Questionnaire (CTQ) and recent trauma scores on the modified World Health Organization's Composite International Diagnostic Interview for post-traumatic stress disorder (WMH-CIDI) scale.
- Plot and test for relationship between trauma with SA of SH (with results from both arms pooled).

Childhood and recent trauma scores

Note: The post-hoc analyses were not initially planned nor described in this study's protocol. These analyses were performed for exploratory purposes to inform the development of future research questions.

Post-hoc analyses

- Determine the mean (range) of the Pain Catastrophizing Scale.
- Determine the mean (range) of the 10-item Connor-Davidson Resilience Scale.

Order effect

Investigate an effect of order, i.e. between-group difference in differential effect when participants are grouped by order, with those who underwent threat first as one group, and those who underwent threat second in the second group.

Exploratory outcomes

Static light touch

- Determine the magnitude of static light touch on each arm, and compare between arms.

Dynamic light touch

- Determine the magnitude of dynamic light touch on each arm, and compare between arms.

Electrical stimulation

- Determine the magnitude of e-stim on each arm, and compare between arms.

Import data

```
master_data <- read_delim("formal_data.csv",
    ";", escape_double = FALSE, trim_ws = TRUE)

demo_info <- read_delim("Demographic_info.csv",
```

```
    ";", escape_double = FALSE, trim_ws = TRUE)

RA_blinding <- read_delim("RA_blinding_ax_final.csv",
    ";", escape_double = FALSE, trim_ws = TRUE)

library(readr)
questionnaire_output_all_tab <- read_delim("C:/Users/Gill/Desktop/questionnaire_output_all.tab.txt",
    delim = "\t", escape_double = FALSE,
    trim_ws = TRUE)
```

Demographic data

```
# Median age and range.
demo_info$Age
```

```
##  [1] 51 19 19 26 21 22 21 21 20 36 21 21 23 55 26 23 20 24 30 23 21 19 19 21 18
## [26] 29
```

```
median(demo_info$Age, na.rm = FALSE)
```

```
## [1] 21
```

```
range(demo_info$Age, na.rm = FALSE)
```

```
## [1] 18 55
```

```
# Sex
demo_info$Sex <- (as.factor(demo_info$Sex))# First change sex to be a character

freq_sex <- table(demo_info$Sex)
view(freq_sex)

# Average detection threshold on each arm across all participants.
mean(demo_info$Detection_threshold_R)# mean detection threshold on the right arm was 1.53
```

```
## [1] 1.534231
```

```
mean(demo_info$Detection_threshold_L)# mean detection threshold on the left was 1.56
```

```
## [1] 1.557692
```

```
# Average detection threshold across both arms across all participants.
mean(demo_info$Intensity_used, trim = 0, na.rm = TRUE)# Overall mean detection threshold was 1.6
```

```
## [1] 1.596154
```

```r
#Standard deviation of individual detection threshold
sd(demo_info$Intensity_used, na.rm = TRUE)
```

```
## [1] 0.6352831
```

Manipulation checks

HFS ratings

A mixed model analysis was used to compare ratings of the HFS trains between conditions. The order in which the two arms were stimulated was kept consistent across participants (right forearm first), but the allocation of forearm to condition was counterbalanced (random allocation to one of two equally sized groups). Therefore, the 'group' and 'site' information have been used to code for a 'condition' variable that is specified as A or B to maintain blinding of the analyst to actual experimental conditions. Here, we test for a main effect of condition on ratings, whilst allowing for a random intercept for each participant.

```r
hfs_1 <- master_data %>% mutate(condition = case_when(
  Group_allocation == '1' & site == 'right' ~ 'A',
  Group_allocation == '1' & site == 'left' ~ 'B',
  Group_allocation == '2' & site == 'right' ~ 'B',
  Group_allocation == '2' & site == 'left' ~ 'A')) %>%
  select(-Group_allocation,
         -site)

hfs <- hfs_1 %>%
  filter(phase == 'hfs') %>%
  select(id, condition, rating)

hfs$rating <- as.numeric(hfs$rating)

hfs %<>% group_by(id, condition) %>%
  mutate(train = row_number()) %>%
  ungroup()

hfs_wide <- hfs %>%
  group_by(id) %>%
  spread(key = condition,
         value = rating)

#Determine the mean (SD) per condition

mean(hfs_wide$A)
```

```
## [1] 38.76538
```

```r
sd(hfs_wide$A)
```

```
## [1] 11.34453
```

```
mean(hfs_wide$B)
```

```
## [1] 39.07231
```

```
sd(hfs_wide$B)
```

```
## [1] 11.31391
```

```
ggplot(data = hfs) +
  aes(x = train,
      y = rating,
      group = id,
      colour = id) +
  geom_point() +
  facet_grid(~ condition) +
  geom_line() +
  guides(colour = guide_legend("ID"), size = guide_legend("ID")) +
  labs( y = 'Ratings',
        x = 'train',
        title = 'Individual SPARS ratings during each HFS train, by site')
```

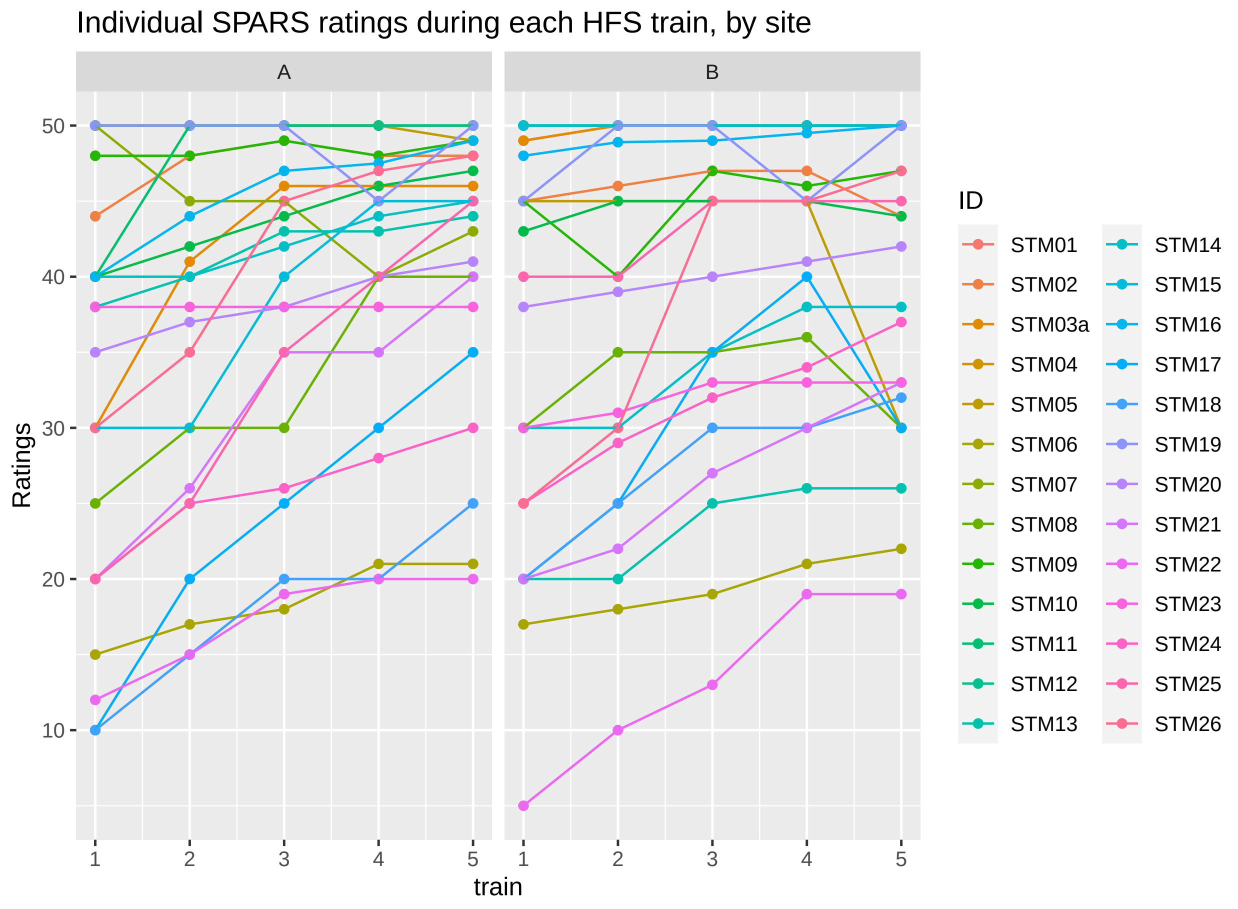

```r
#remove blinding to make graph clear
hfs$condition[hfs$condition == 'A'] <- 'Threat site'

hfs$condition[hfs$condition == 'B'] <- 'Control site'

ggplot(data = hfs) +
  aes(x = train,
      y = rating,
      group = condition,
      colour = condition) +
  geom_point() +
    geom_jitter(width = 0.3) +
    scale_y_continuous(limits = c(0 , 50)) +
  scale_colour_manual(values = c("#009E73", "#D55E00")) +
  guides(colour = guide_legend("Condition"), size = guide_legend("Condition")) +
  labs( y = 'SPARS ratings',
        x = 'Train')
```

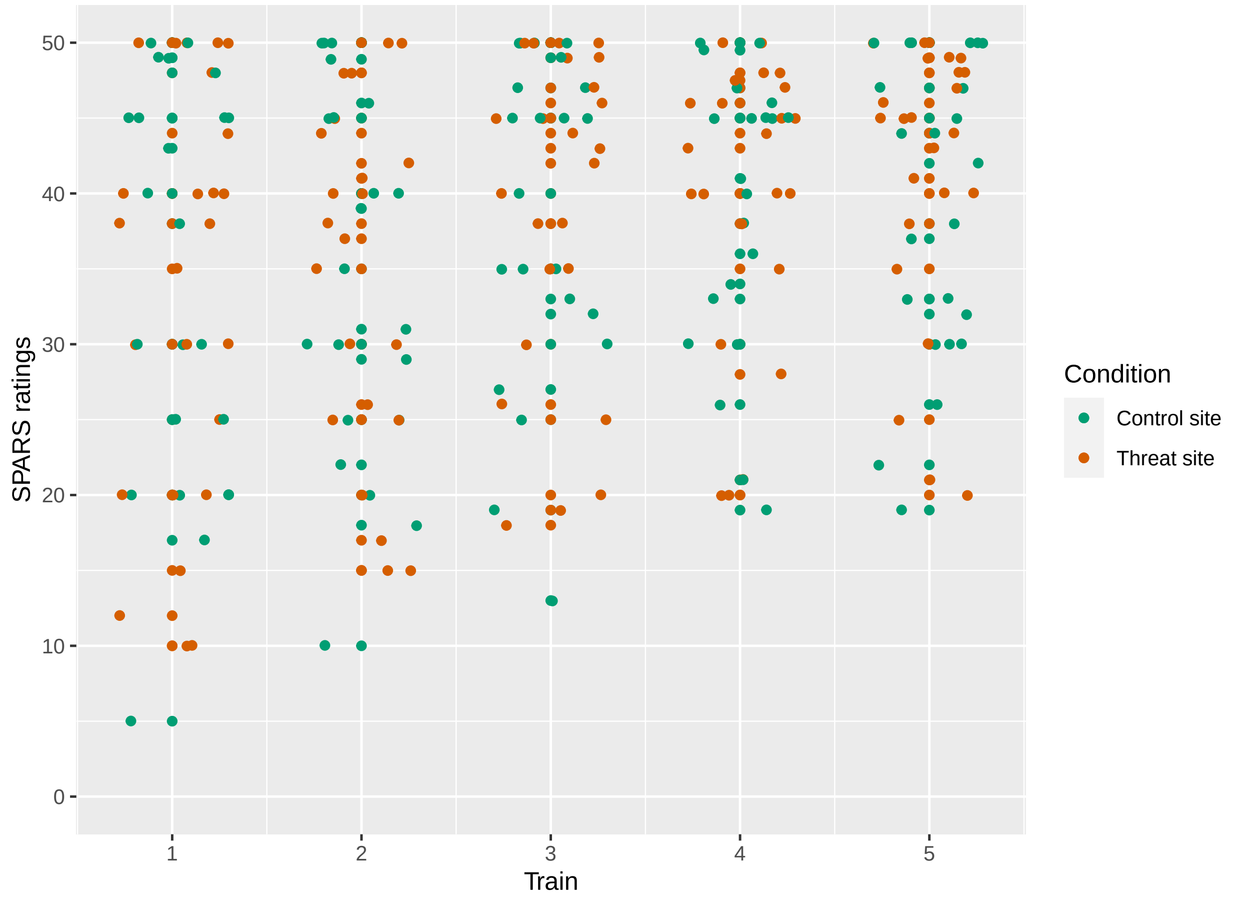


```r
ggplot(data = hfs) +
  aes(x = condition,
      y = rating,
      group = condition,
```

```r
    colour = condition)  +
  geom_boxplot(width = 2)  +
  geom_jitter(width = 0.4) +
  scale_colour_manual(values = c("#009E73", "#D55E00")) +
  guides(colour = guide_legend("Condition"), size = guide_legend("Condition")) +
  labs(y = 'SPARS rating (range: -50 to +50) during HFS induction trains',
       x = 'Condition')
```

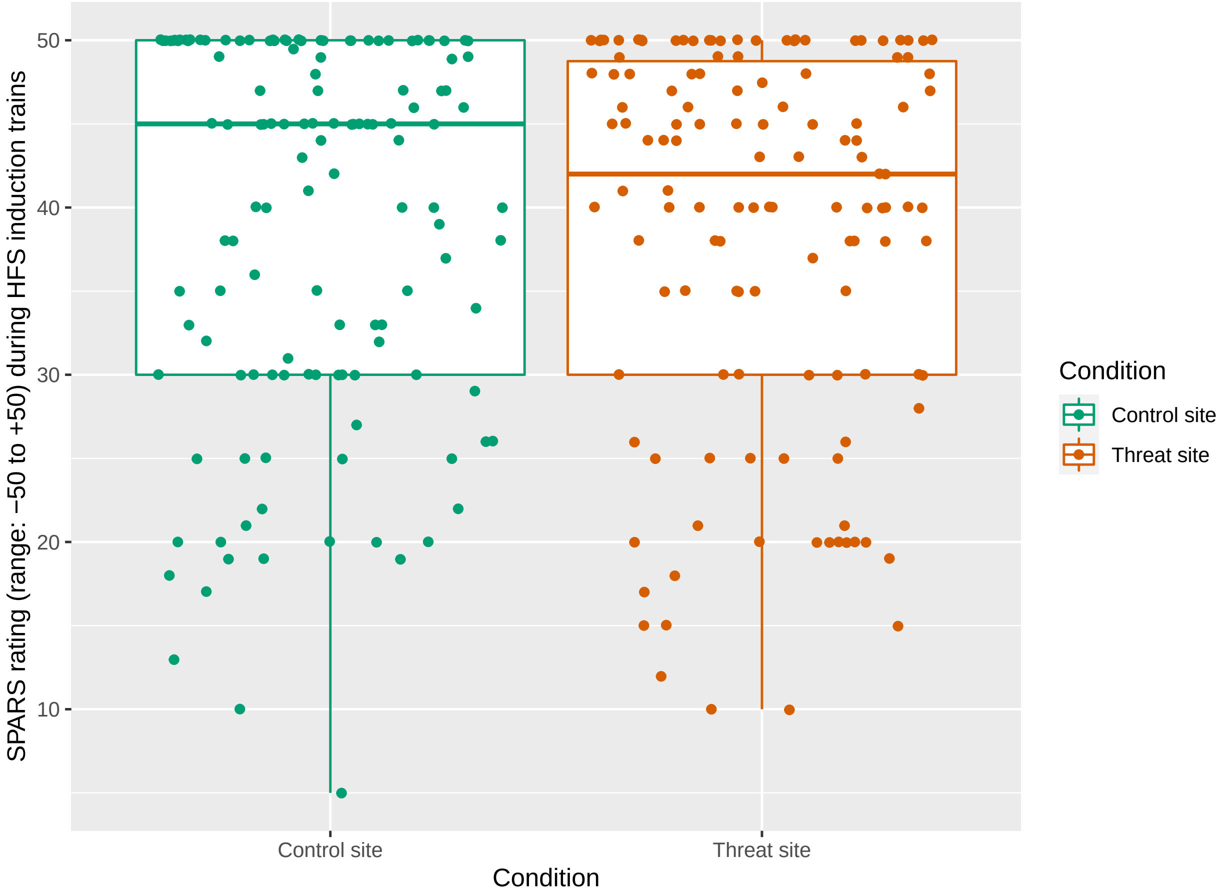


```r
range(hfs$rating)
```

```
## [1]  5 50
```

```r
# Null model
hfs_null <- lmer(rating ~ 1 + (1|id),
                 data = hfs)
hfs_condition <- lmer(rating ~ condition + (1|id),
                 data = hfs)
anova(hfs_condition, hfs_null) # hfs_condition is no better than null
```

```
## Data: hfs
## Models:
```

```
## hfs_null: rating ~ 1 + (1 | id)
## hfs_condition: rating ~ condition + (1 | id)
##               npar    AIC    BIC  logLik deviance  Chisq Df Pr(>Chisq)
## hfs_null         3 1710.2 1720.9 -852.11   1704.2
## hfs_condition    4 1712.0 1726.3 -852.01   1704.0 0.2126  1     0.6447
```

```r
summary(hfs_condition)
```

```
## Linear mixed model fit by REML. t-tests use Satterthwaite's method [
## lmerModLmerTest]
## Formula: rating ~ condition + (1 | id)
##    Data: hfs
##
## REML criterion at convergence: 1699.8
##
## Scaled residuals:
##     Min      1Q  Median      3Q     Max
## -3.3241 -0.4205  0.1031  0.5164  2.3287
##
## Random effects:
##  Groups   Name        Variance Std.Dev.
##  id       (Intercept) 102.63   10.131
##  Residual              28.91    5.376
## Number of obs: 260, groups:  id, 26
##
## Fixed effects:
##                      Estimate Std. Error       df t value Pr(>|t|)
## (Intercept)           39.0723     2.0420  26.3864   19.14   <2e-16 ***
## conditionThreat site  -0.3069     0.6669 233.0000   -0.46    0.646
## ---
## Signif. codes:  0 '***' 0.001 '**' 0.01 '*' 0.05 '.' 0.1 ' ' 1
##
## Correlation of Fixed Effects:
##             (Intr)
## cndtnThrtst -0.163
```

```r
# Conclude: HFS ratings were not predicted by condition.

model_condition <- lmer(rating ~
                          condition +(1|id),
                                     REML = TRUE,
                                     data = hfs) #Fitting a model using REML

anova(model_condition) #Performs F test on fixed effects using Satterthwaite approximation
```

```
## Type III Analysis of Variance Table with Satterthwaite's method
##           Sum Sq Mean Sq NumDF DenDF F value Pr(>F)
## condition 6.1231  6.1231     1   233  0.2118 0.6458
```

```r
summary(model_condition) #gives model output with estimated df and p values using Satterthwaite
```

```
## Linear mixed model fit by REML. t-tests use Satterthwaite's method [
```

```
## lmerModLmerTest]
## Formula: rating ~ condition + (1 | id)
##    Data: hfs
##
## REML criterion at convergence: 1699.8
##
## Scaled residuals:
##     Min      1Q  Median      3Q     Max
## -3.3241 -0.4205  0.1031  0.5164  2.3287
##
## Random effects:
##  Groups   Name        Variance Std.Dev.
##  id       (Intercept) 102.63   10.131
##  Residual              28.91    5.376
## Number of obs: 260, groups:  id, 26
##
## Fixed effects:
##                      Estimate Std. Error       df t value Pr(>|t|)
## (Intercept)           39.0723     2.0420  26.3864   19.14   <2e-16 ***
## conditionThreat site  -0.3069     0.6669 233.0000   -0.46    0.646
## ---
## Signif. codes:  0 '***' 0.001 '**' 0.01 '*' 0.05 '.' 0.1 ' ' 1
##
## Correlation of Fixed Effects:
##             (Intr)
## cndtnThrtst -0.163
```

```
# Conclusion: HFS ratings were not predicted by condition.
```

Anxiety ratings

We are interested in a main effect of condition on anxiety ratings.

```
anxiety <- master_data %>% select(Group_allocation,
                                  id,
                                  anxious_right,
                                  anxious_left)
anxiety <- unique(anxiety)

anxiety %<>%
  gather(key = arm,
         value = anxiety,
         3:4) %>%
  mutate(arm = case_when(
    arm == 'anxious_left' ~ 'left',
    arm == 'anxious_right' ~ 'right'
  ))

anxiety %<>% mutate(condition = case_when(
  Group_allocation == '1' & arm == 'right' ~ 'A',
  Group_allocation == '1' & arm == 'left' ~ 'B',
  Group_allocation == '2' & arm == 'right' ~ 'B',
  Group_allocation == '2' & arm == 'left' ~ 'A')) %>%
```

```r
  select(-Group_allocation)

anxiety_wide <- anxiety %>%
  group_by(id) %>%
  spread(key = condition,
         value = anxiety) %>%
  select(-arm)

#Determining the mean (SD) anxiety rating per condition
mean(anxiety_wide$A, na.rm = TRUE)
```

```
## [1] 3.307692
```

```r
sd(anxiety_wide$A, na.rm = TRUE)
```

```
## [1] 1.123182
```

```r
mean(anxiety_wide$B, na.rm = TRUE)
```

```
## [1] 3.423077
```

```r
sd(anxiety_wide$B, na.rm = TRUE)
```

```
## [1] 1.137474
```

```r
var(anxiety_wide$B, na.rm = TRUE)
```

```
## [1] 1.293846
```

```r
#remove blinding to make graph clear
anxiety$condition[anxiety$condition == 'A'] <- 'Threat site'

anxiety$condition[anxiety$condition == 'B'] <- 'Control site'

ggplot(data = anxiety) +
  aes(x = condition,
      y = anxiety,
      group = interaction(condition),
     colour = condition) +
  geom_boxplot(alpha = 0.6) +
  geom_point() +
  theme(panel.grid.minor = element_blank()) +
    geom_jitter( height = 0, width = 0.4) +
  scale_colour_manual(values = c("#009E73", "#D55E00")) +
  guides(colour = guide_legend("Condition"), size = guide_legend("Condition")) +
 labs(y = 'Anxiety rating',
       x = 'Condition')
```

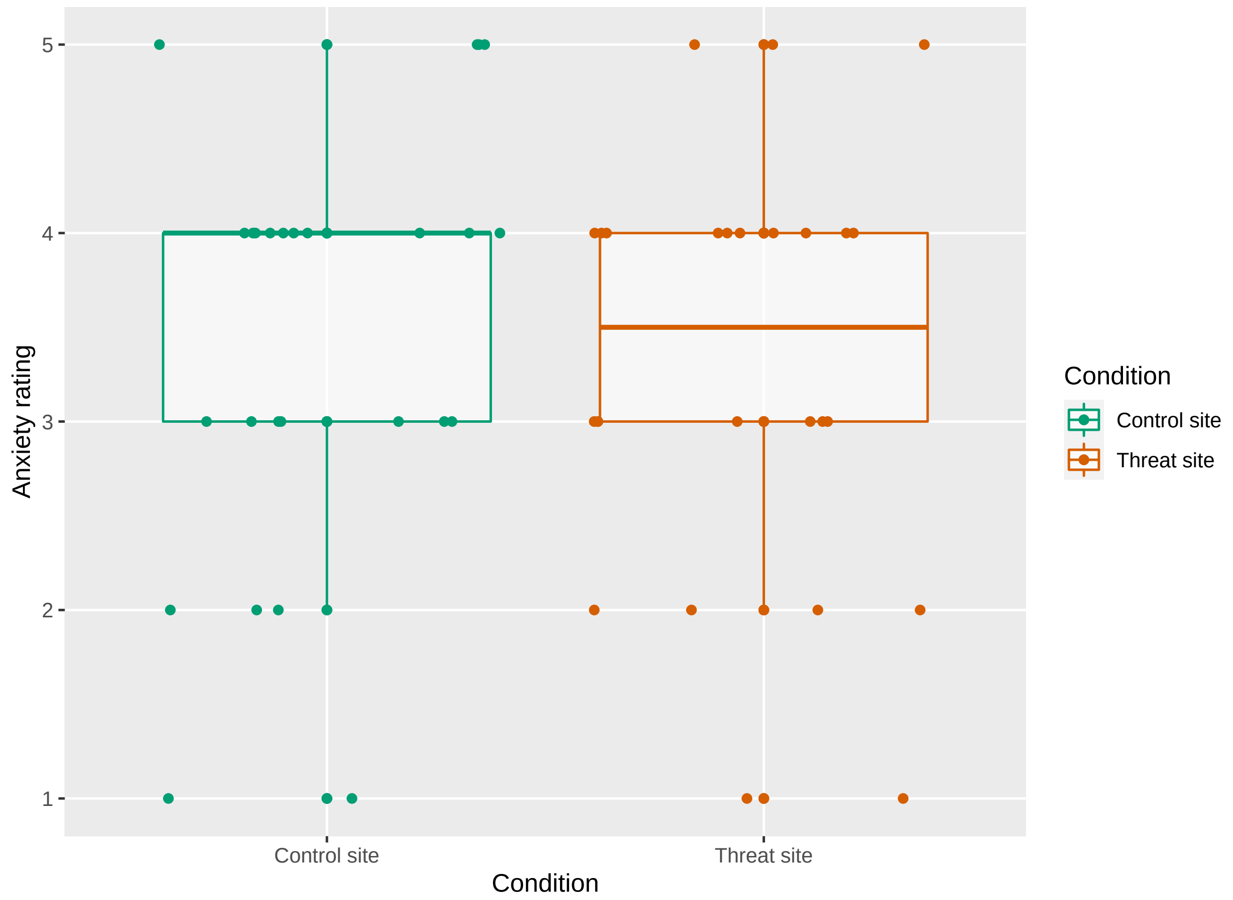


```
a1 <- ggplot(anxiety) +
  aes(x = arm,
      y = anxiety,
      group = condition,
      colour = condition) +
  theme(panel.grid.minor = element_blank()) +
  geom_point() +
  facet_grid(~ id) +
  labs(title = 'Individual effect of condition on anxiety rating',
       y = 'Anxiety ratings',
       x = 'Arm')

anxiety_null <- lmer(anxiety ~ 1 + (1|id),
                     data = anxiety)
anxiety_condition <- lmer(anxiety ~ condition + (1|id),
                     data = anxiety)
anova(anxiety_condition, anxiety_null) # No effect of condition.
```

```
## Data: anxiety
## Models:
## anxiety_null: anxiety ~ 1 + (1 | id)
## anxiety_condition: anxiety ~ condition + (1 | id)
##                   npar    AIC    BIC  logLik deviance  Chisq Df Pr(>Chisq)
```

```
## anxiety_null         3 129.51 135.37 -61.758   123.52
## anxiety_condition    4 130.50 138.30 -61.248   122.50 1.0197  1     0.3126
```

Threat ratings

We are interested in a main effect of condition on threat ratings.

```
threat <- master_data %>% select(Group_allocation,
                                  id,
                                  damage_right,
                                  damage_left)
threat <- unique(threat)

threat %<>%
  gather(key = arm,
         value = threat,
         3:4) %>%
  mutate(arm = case_when(
    arm == 'damage_left' ~ 'left',
    arm == 'damage_right' ~ 'right'
  ))

threat %<>% mutate(condition = case_when(
  Group_allocation == '1' & arm == 'right' ~ 'A',
  Group_allocation == '1' & arm == 'left' ~ 'B',
  Group_allocation == '2' & arm == 'right' ~ 'B',
  Group_allocation == '2' & arm == 'left' ~ 'A')) %>%
  select(-Group_allocation)

threat_wide <- threat %>%
    select(-arm) %>%
  group_by(id) %>%
  spread(key = condition,
         value = threat)

# Assessing for correlation in threat ratings across condition sites

plot(jitter(threat_wide$A), jitter(threat_wide$B))
```

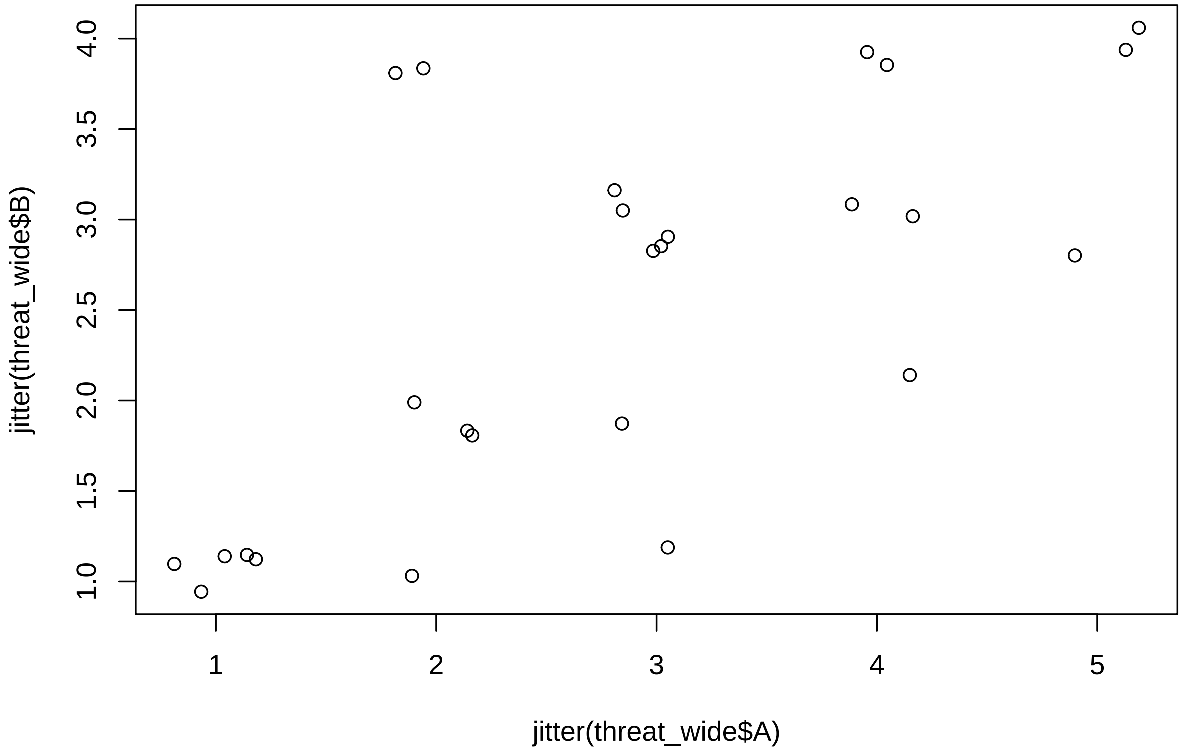


```
plot_threat_cor <- threat$threat

hist(plot_threat_cor, breaks=30) #Histogram
```

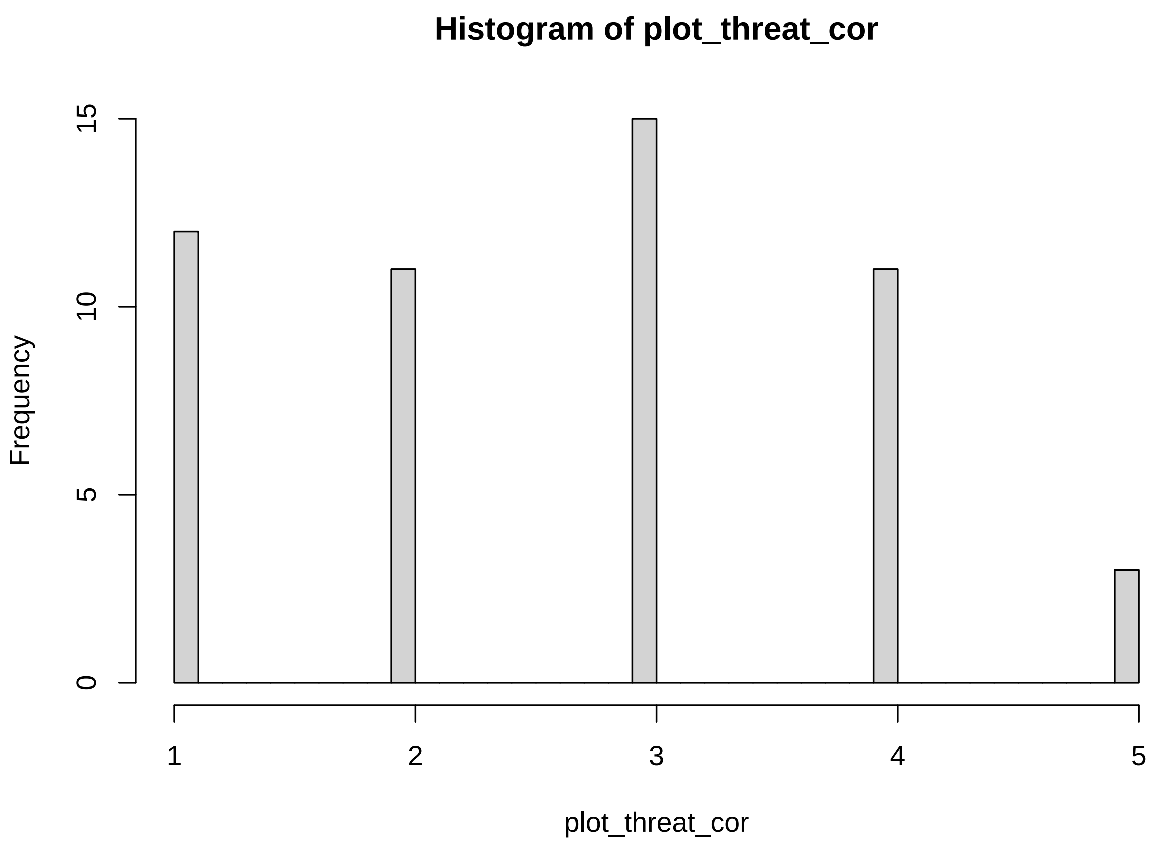


```
with(threat, shapiro.test(threat)) # P = 0.00038
```

```
##
##  Shapiro-Wilk normality test
##
## data:  threat
## W = 0.90068, p-value = 0.0003886
```

```
correlation <- cor.test(threat_wide$A, threat_wide$B, method = "spearman")
correlation # significant
```

```
##
##  Spearman's rank correlation rho
##
## data:  threat_wide$A and threat_wide$B
## S = 943.09, p-value = 0.0001431
## alternative hypothesis: true rho is not equal to 0
## sample estimates:
##       rho
## 0.6775763
```

```r
#Determining the mean (SD) threat rating per condition
mean(threat_wide$A, na.rm = TRUE)
```

```
## [1] 2.807692
```

```r
sd(threat_wide$A, na.rm = TRUE)
```

```
## [1] 1.296741
```

```r
mean(threat_wide$B, na.rm = TRUE)
```

```
## [1] 2.5
```

```r
sd(threat_wide$B, na.rm = TRUE)
```

```
## [1] 1.140175
```

```r
#remove blinding to make graph clear
threat$condition[threat$condition == 'A'] <- 'Threat site'

threat$condition[threat$condition == 'B'] <- 'Control site'

ggplot(data = threat) +
  aes(x = condition,
      y = threat,
      group = interaction(condition),
      colour = condition) +
  geom_boxplot(alpha = 0.6) +
  theme(panel.grid.minor = element_blank()) +
  geom_point () +
geom_jitter(height = 0, width = 0.4) +
  scale_colour_manual(values = c("#009E73", "#D55E00")) +
  guides(colour = guide_legend("Condition"), size = guide_legend("Condition")) +
  labs(y = 'Threat of tissue damage ratings',
       x = 'Condition')
```

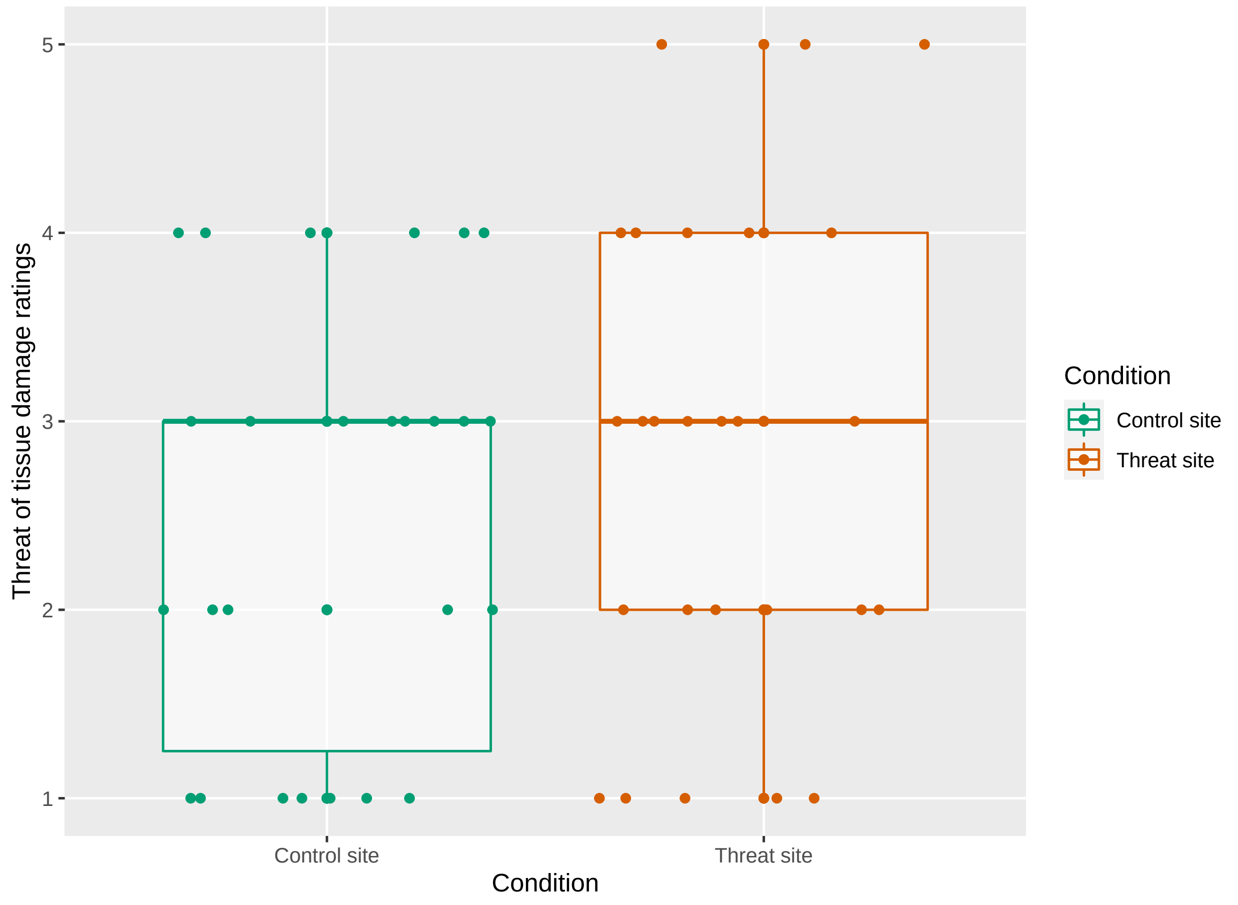


```
ggplot(data = threat) +
  aes(x = arm,
      y = threat,
      group = condition,
      colour = condition) +
  geom_point() +
  facet_wrap(~ id,
             nrow = 3) +
    guides(colour = guide_legend("Condition"), size = guide_legend("Condition")) +
  labs(title = 'The individual effect of condition on arm of tissue damage rating',
       y = 'Threat of tissue damage ratings',
       x = 'Arm')
```

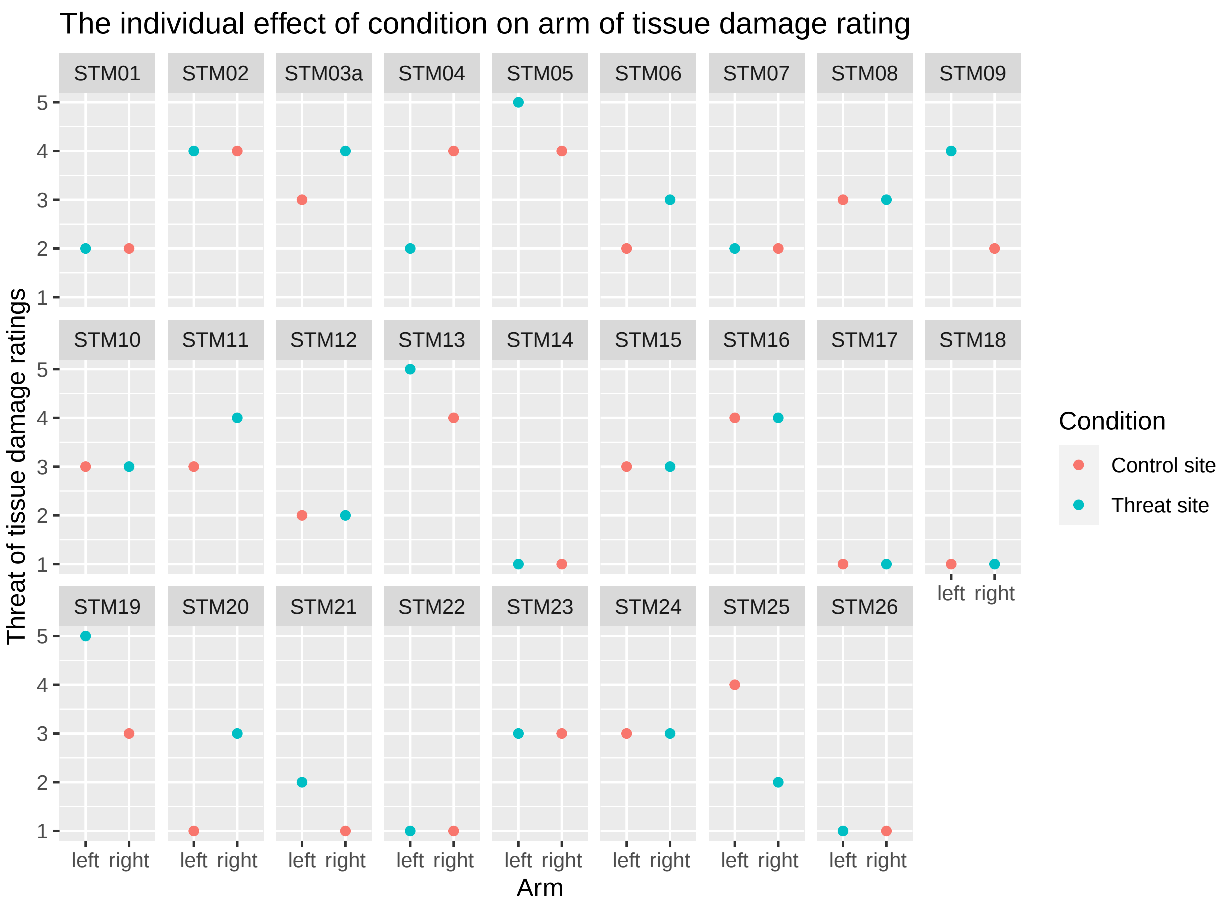


```
threat_null <- lmer(threat ~ 1 + (1|id),
                     data = threat)
threat_condition <- lmer(threat ~ condition + (1|id),
                     data = threat)
anova(threat_condition, threat_null) # No effect of condition.
```

```
## Data: threat
## Models:
## threat_null: threat ~ 1 + (1 | id)
## threat_condition: threat ~ condition + (1 | id)
##                  npar    AIC    BIC  logLik deviance Chisq Df Pr(>Chisq)
## threat_null         3 158.46 164.32 -76.232   152.46
## threat_condition    4 157.88 165.68 -74.939   149.88 2.586  1     0.1078
```

Blinding assessment

```
freq <- table(RA_blinding$threat_arm_RA)

barplot(freq, main = "Frequency with which research assistant believed  the HFS to have been \ndelivered
```

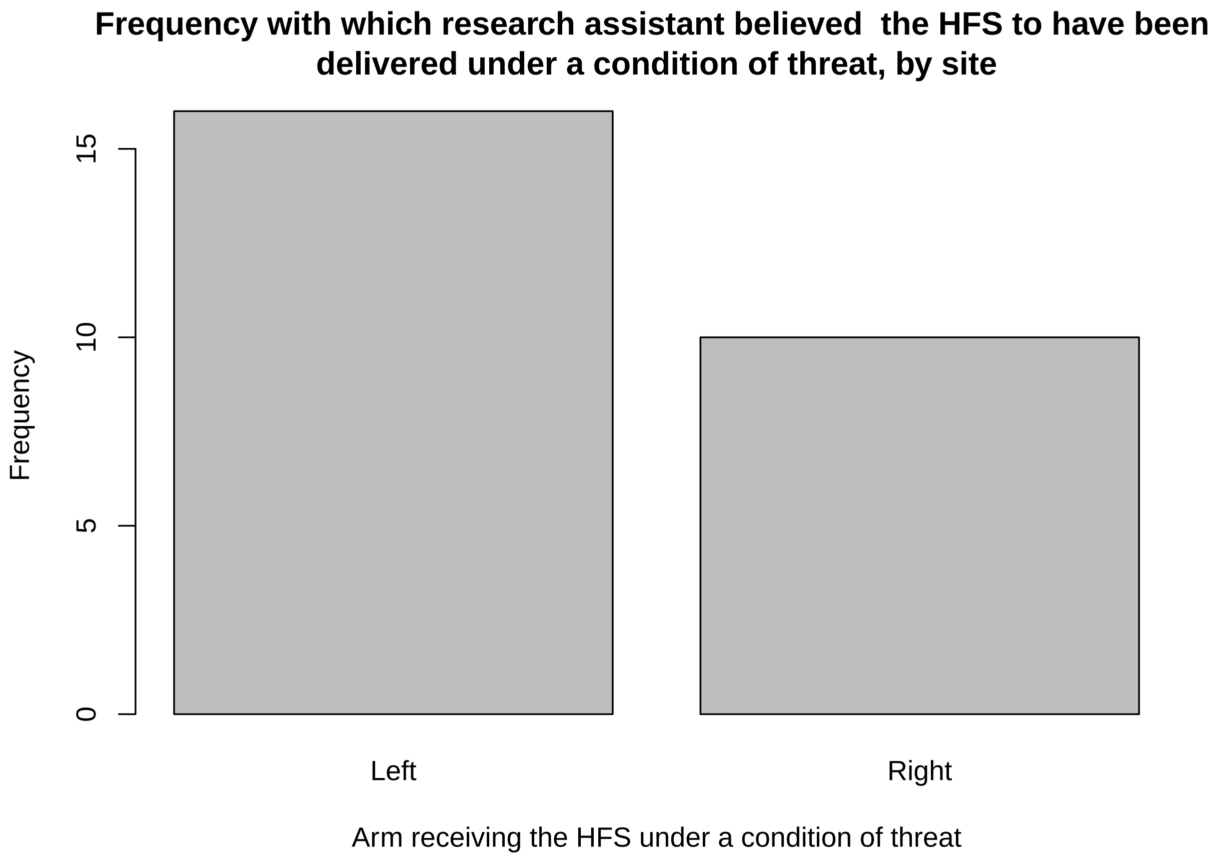


```
# compare RA's guesses to actual group allocation

actual_condition <- master_data %>%
  select(id,
         Group_allocation)

actual_condition <- unique(actual_condition)

names(actual_condition)[names(actual_condition) == 'Group_allocation'] <- 'threat_arm_actual'

# rename "group 1" to "right" and "group 2" to "left"

actual_condition %<>% mutate(threat_arm_actual = case_when(
  threat_arm_actual == '1' ~ 'Right',
  threat_arm_actual == '2' ~ 'Left'))

comparison <- merge(x = actual_condition, y = RA_blinding, by.x = 'id' , by.y = 'id' , all = TRUE)

# Now add a new column to see whether RA was correct or not. When threat_arm_actual and threat_arm_RA a

comparison %<>% mutate(accuracy = if_else(threat_arm_actual == threat_arm_RA, 'Correct','Incorrect'))

#Determine the percentage of correct responses
```

```r
Correctness <- table(comparison$accuracy)

view(Correctness)

# Percentage correct: 42.31%
(11/26)*100
```

```
## [1] 42.30769
```

```r
# Determine Cohen's kappa coefficient

blinding_ax_table <- table(comparison$threat_arm_actual,
                              comparison$threat_arm_RA)
blinding_ax_table
```

```
##
##          Left Right
##   Left      7     6
##   Right     9     4
```

```r
p0 <- (7 + 9 )/26
pa <- (13/26)*(16/26)
pb <- (13/26)*(10/26)
pe <- pa + pb

  kappa <- (p0 - pe) / (1 - pe)  #Kappa is 0.23

  # Add anchors for the confidence level scale

comparison %<>% mutate(Confidence_level = case_when(
    Confidence_level == '1' ~ 'Not at all confident',
    Confidence_level == '2' ~ 'Not confident',
    Confidence_level == '3' ~ 'Neutral',
    Confidence_level == '4' ~ 'Confident',
    Confidence_level == '5' ~ 'Extremely confident'))


# Plot degree of correctness

ggplot(data = comparison) +
  aes(x = accuracy,
      y = Confidence_level) +
geom_boxplot(width = 5) +
  geom_point(position = 'jitter') +
    scale_y_discrete(limits = c('Not at all confident', 'Not confident', 'Neutral', 'Confident' ,'Extrem
  labs(title = ' Boxplots showing the relationship between \nconfidence level and accuracy \nof guessin
       y = 'Confidence level',
       x = 'Number of guesses correct or incorrect (i.e accuracy)')
```

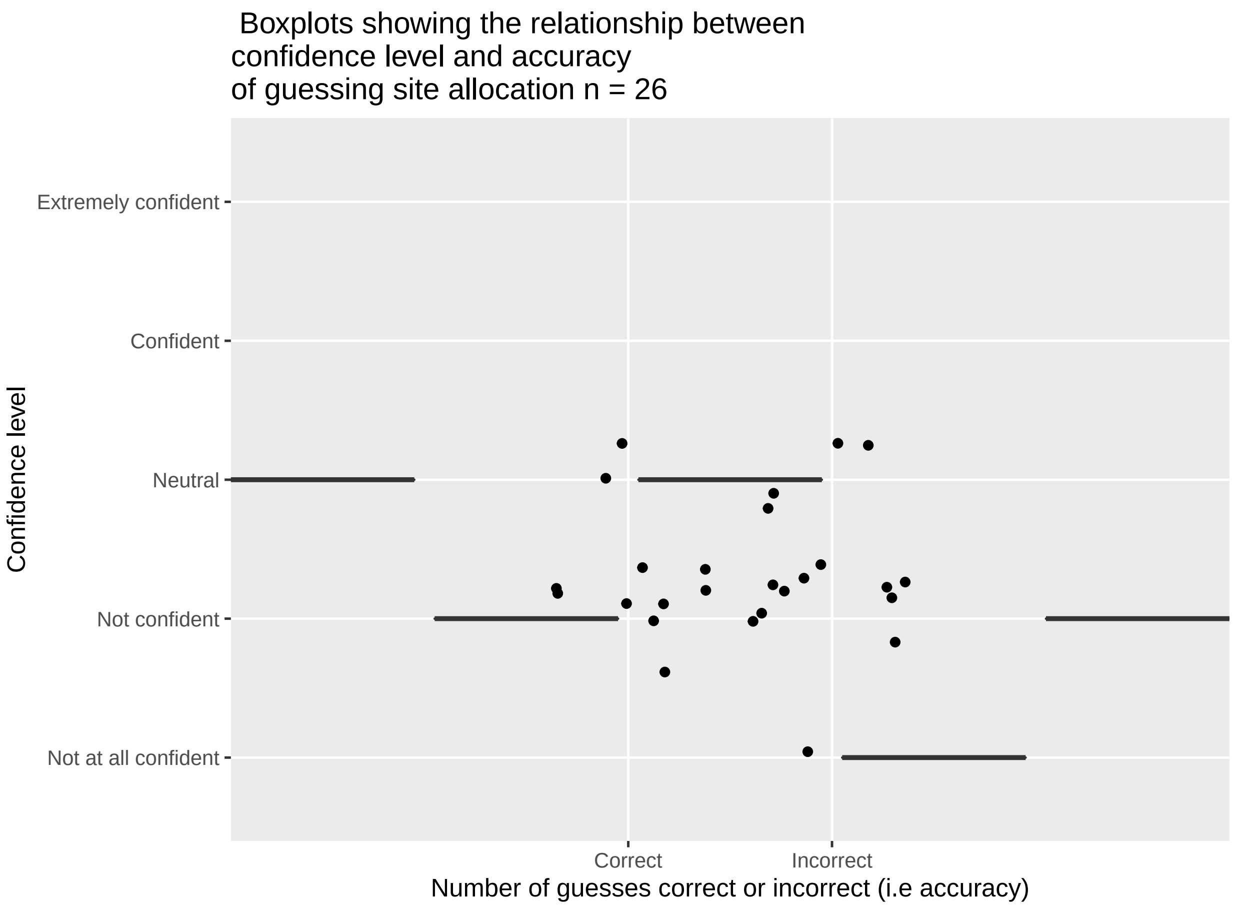


```
# Could the RA reliably predict condition? No: this plot shows that the RA made confident assertions th
```

Primary outcome: magnitude of secondary hyperalgesia

Aim: to determine the magnitude of secondary hyperalgesia (SH) on each arm, and the comparison between arms.

Data were from the baseline time points as well as follow-up time points, and for both arms (control and threat sites). The main interest was the change in ratings (from baseline to follow-up time points), and how these differed between the arms/conditions. Therefore, for the formal analysis, researchers: 1) calculated the mean rating for all baseline time points; 2) calculated the mean rating for all follow-up time points; 3) subtracted the mean baseline rating from the mean follow-up rating (within participant and condition), to provide the indication of change in rating (i.e. magnitude of hyperalgesia) for each condition, within each participant.

```
intensity <- master_data %>%
  select(Group_allocation,
         id,
         time,
         phase,
         site,
         modality,
         rating) %>%
```

```r
filter(modality == '128' | modality == '256') %>%
filter(phase != 'orientation') %>%
mutate(time = if_else(phase == 'baseline', time*-1L, time))

intensity %<>% mutate(condition = case_when(
  Group_allocation == '1' & site == 'right' ~ 'A',
  Group_allocation == '1' & site == 'left' ~ 'B',
  Group_allocation == '2' & site == 'right' ~ 'B',
  Group_allocation == '2' & site == 'left' ~ 'A')) %>%
  select(-Group_allocation,
         -site)

intensity$rating <- as.numeric(intensity$rating)
intensity %<>% group_by(id,
           time,
           phase,
           condition) %>%
  summarise(pp_rating = mean(rating))


int_bl <- intensity %>% filter(phase == 'baseline') %>%
  group_by(id, condition) %>%
  summarise(rating = mean(pp_rating)) %>%
  ungroup() %>%
  mutate(time = -4) %>% ungroup()

int_fu <- intensity %>% filter(phase != 'baseline') %>% ungroup () %>%
  select(-phase) %>%
  rename(rating = pp_rating)

intensity1 <- rbind(int_bl, int_fu)

# For interest, plot the actual data

ggplot(data = intensity1) +
  aes(x =time,
      y = rating,
      group = condition,
      colour = condition) +
  facet_wrap(~ id) +
  geom_point() +
  geom_line() +
  geom_hline(yintercept = 0) +
  labs(title = 'Individual intensity of secondary hyperalgesia, over time',
       y = 'Rating',
       x = 'Time (minutes)')
```

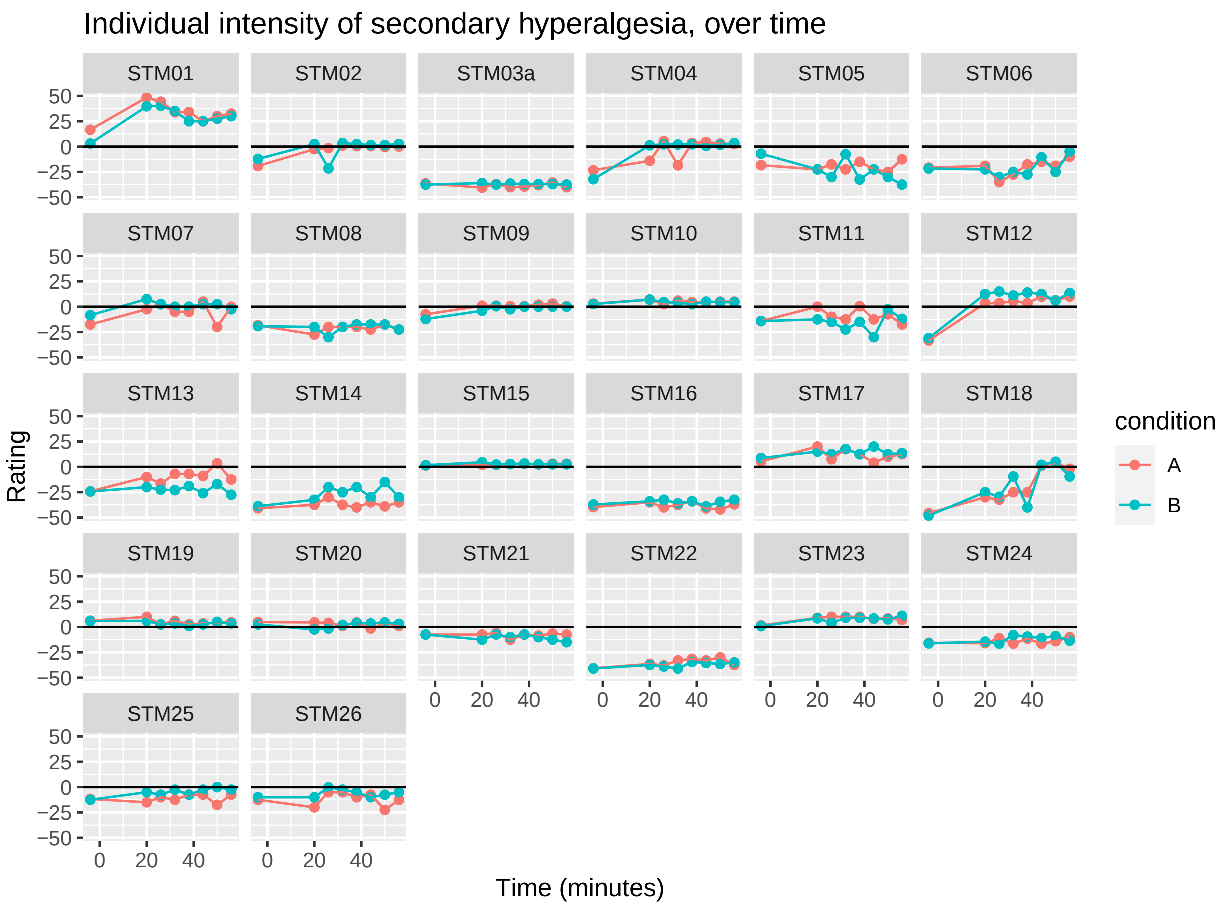


```
# Plot group mean ratings by condition over time

mean_intensity <- intensity1 %>%
  group_by(condition, time) %>%
  summarise(mean_rating = mean(rating, na.rm=TRUE))

# Plot group intensity of secondary hyperalgesia by condition, over time

intensity_groupmean <- intensity %>%
  ungroup() %>%
  select(-phase) %>%
  group_by(time, condition) %>%
  summarise(rating = mean(pp_rating)) %>%
  ungroup()

intensity_groupmean$time <- as.factor(intensity_groupmean$time)

 # Calculate difference between conditions for group boxplots

intensity_wide <- intensity1 %>%
  group_by(id,
           time) %>%
  spread(key = condition,
         value = rating) %>%
```

```
  mutate(site_diff = A-B)


#remove blinding to make graph clear
intensity_groupmean$condition[intensity_groupmean$condition == 'A'] <- 'Threaten site'

intensity_groupmean$condition[intensity_groupmean$condition == 'B'] <- 'Control site'

ggplot(data = intensity_groupmean) +
    aes(x = time,
      y = rating,
      group = condition,
      colour = condition,
      xmax = '60') +
  geom_point() +
  geom_vline(xintercept = 3.1, colour = 'red') +
  guides(colour = guide_legend("Condition"), size = guide_legend("Condition"),
  shape = guide_legend("Condition")) +
  labs(title = 'Mean intensity of secondary hyperalgesia at each time point, \ngrouped by condition n =
       y = 'SPARS rating',
       x = 'Time (minutes)')
```

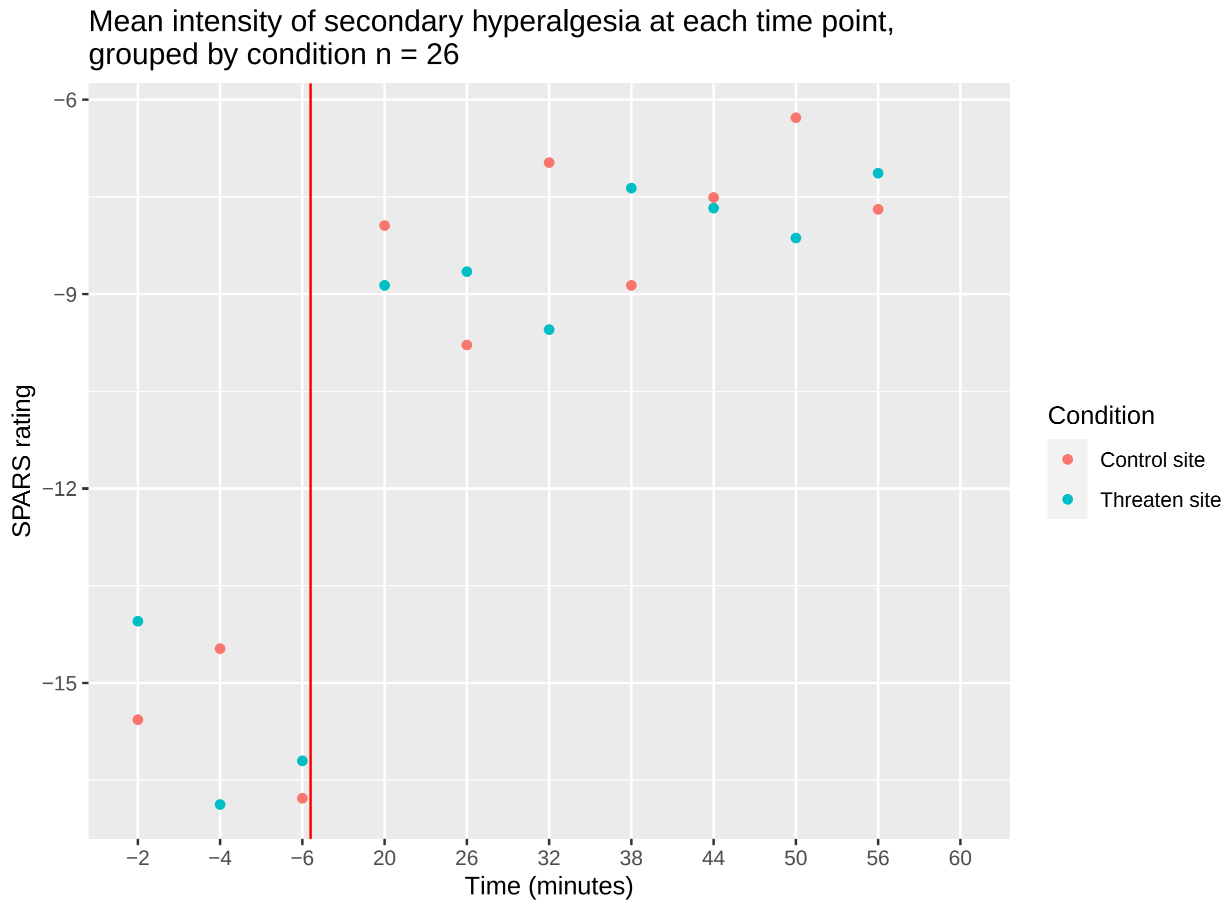

```r
# All participants, group data represented by boxplots

#remove blinding to make graph clear
intensity1$condition[intensity1$condition == 'A'] <- 'Threat site'

intensity1$condition[intensity1$condition == 'B'] <- 'Control site'

intensity1 %>%
ggplot(data = .) +
  aes(x = factor(time),
      y = rating,
      colour = condition) +
  geom_hline(yintercept = 0, colour = 'blue') +
  geom_boxplot() +
  geom_vline(xintercept = 1.4, colour = 'orange') +
  geom_boxplot(outlier.shape = NA) +
  geom_point(position = position_jitterdodge(jitter.width = 0.2)) +
  scale_colour_manual(values = c("#009E73", "#D55E00")) +
  guides(colour = guide_legend("Condition"), size = guide_legend("Condition")) +
  labs(y = 'SPARS rating',
       x = 'Time (mins) relative to induction')
```

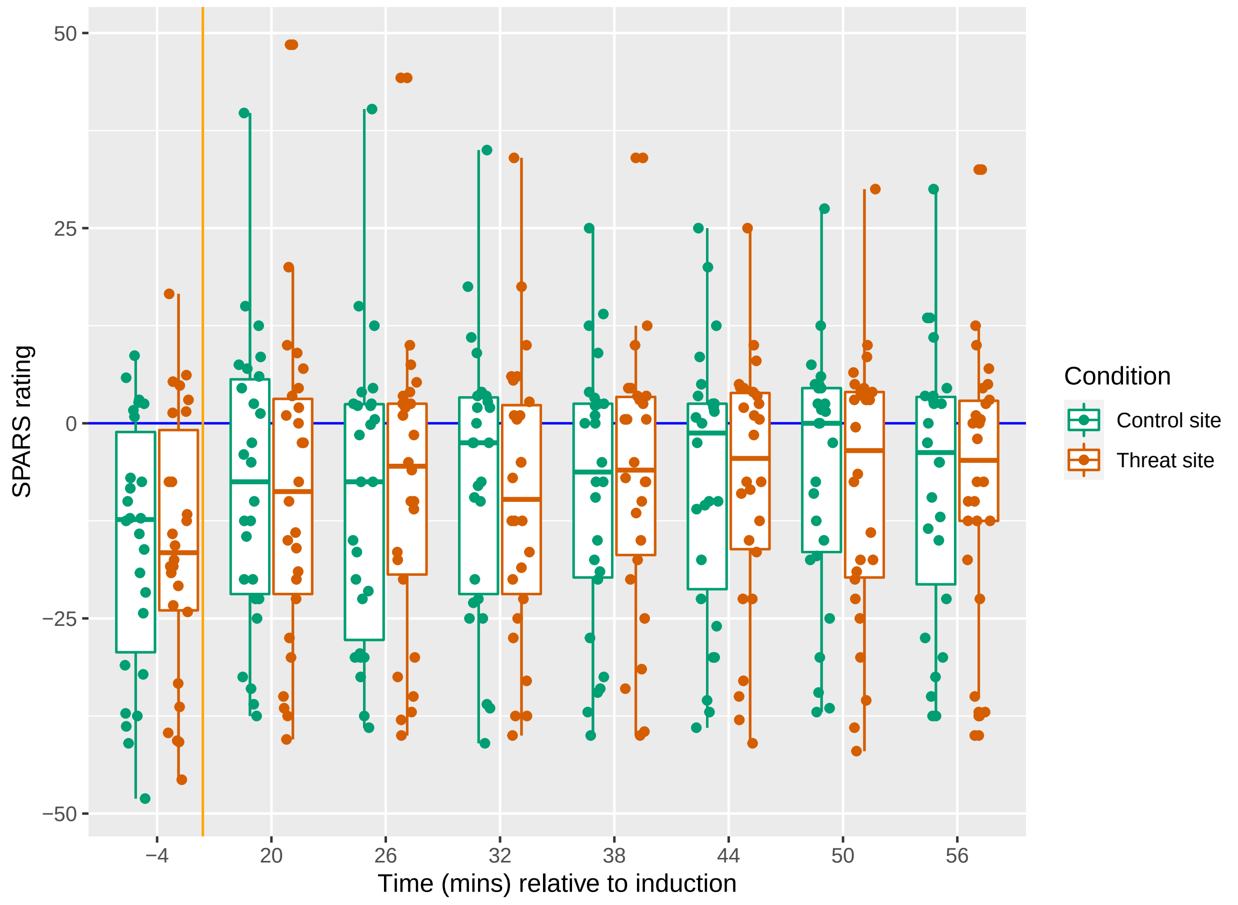

```
# All participants, group data represented by boxplots
ggplot(data = intensity_wide) +
  aes(x = time,
      y = site_diff,
      group = time) +
  geom_hline(yintercept = 0, colour = 'blue') +
  geom_boxplot(width = 2) +
  geom_vline(xintercept = 0, colour = 'orange') +
  geom_jitter(width = 1.2) +
  labs(y = 'Pinprick rating at threat site minus rating at control site',
       x = 'Time (mins) relative to induction')
```

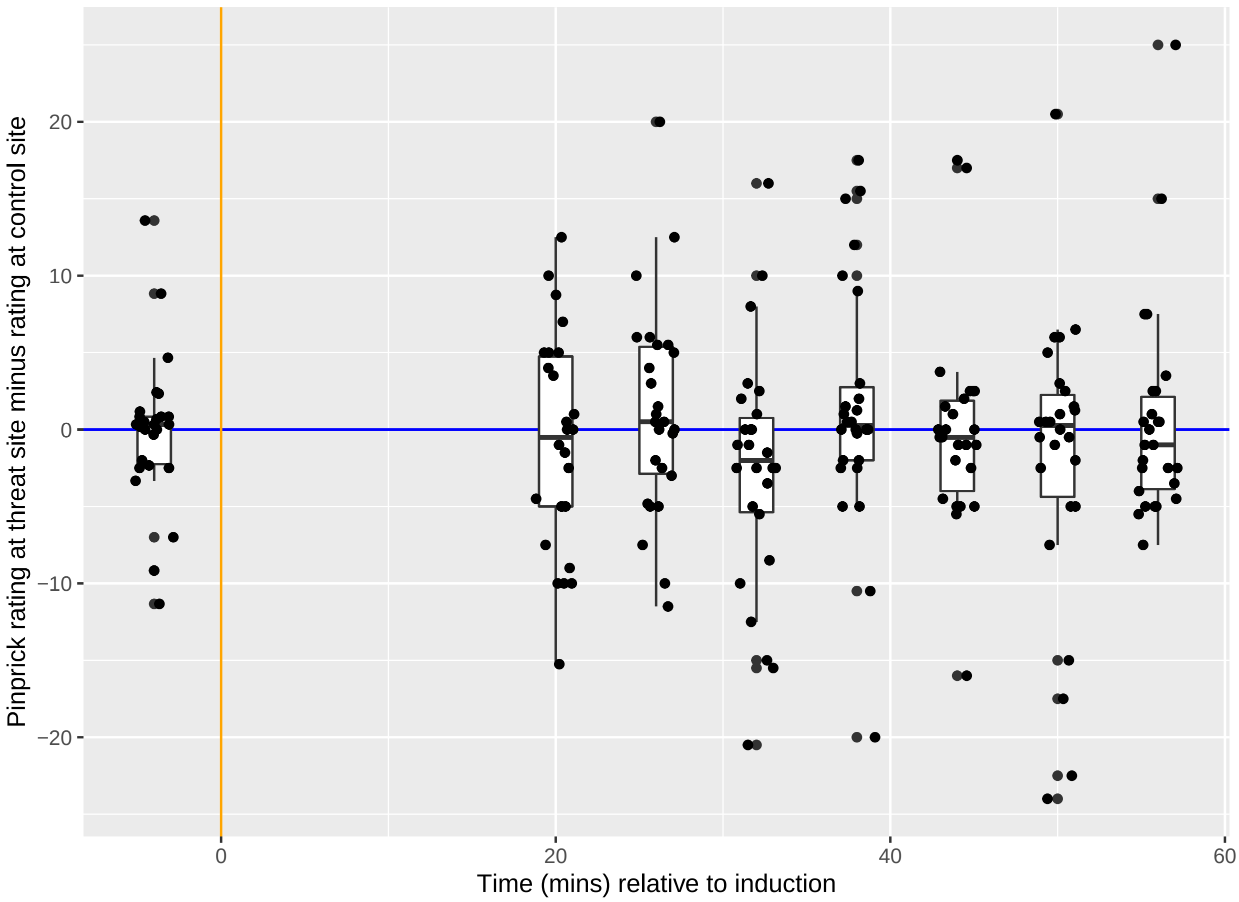


```
# Collapse all post-baseline ratings for each condition and then compare them using a RM ANOVA type mod

int_bl %<>% select(-time)
names(int_bl)[names(int_bl) == 'rating'] <- 'baseline_rating'
collapse <- int_fu %>% # resolve each rating relative to baseline
  right_join(int_bl) %>%
  mutate(rating_controlled = rating-baseline_rating) %>%
  select(id,
         time,
         condition,
```

```r
        rating_controlled) # spread to calculate difference between conditions

# We want to find out whether rating_controlled (for baseline) is predicted by condition.

model_null <- lmer(rating_controlled ~ (1|id),
                   data = collapse)
model_condition <- lmer(rating_controlled ~ condition + (1|id),
                       data = collapse)

anova(model_condition, model_null) # No improvement
```

```
## Data: collapse
## Models:
## model_null: rating_controlled ~ (1 | id)
## model_condition: rating_controlled ~ condition + (1 | id)
##                 npar    AIC    BIC  logLik deviance  Chisq Df Pr(>Chisq)
## model_null         3 2492.6 2504.3 -1243.3   2486.6
## model_condition    4 2494.5 2510.1 -1243.2   2486.5 0.1168  1     0.7326
```

```r
model_pre_condition_crossed <- lmer(rating_controlled ~ condition + (1|id/time),
                                    data = collapse)
summary(model_pre_condition_crossed)
```

```
## Linear mixed model fit by REML. t-tests use Satterthwaite's method [
## lmerModLmerTest]
## Formula: rating_controlled ~ condition + (1 | id/time)
##    Data: collapse
##
## REML criterion at convergence: 2480.7
##
## Scaled residuals:
##     Min      1Q  Median      3Q     Max
## -3.6524 -0.3753  0.0285  0.4071  3.0526
##
## Random effects:
##  Groups   Name        Variance Std.Dev.
##  time:id  (Intercept)   3.567   1.889
##  id       (Intercept) 134.624  11.603
##  Residual              38.141   6.176
## Number of obs: 364, groups:  time:id, 182; id, 26
##
## Fixed effects:
##              Estimate Std. Error       df t value Pr(>|t|)
## (Intercept)    7.5119     2.3253  25.9964   3.231  0.00334 **
## conditionB     0.2303     0.6474 181.0001   0.356  0.72250
## ---
## Signif. codes:  0 '***' 0.001 '**' 0.01 '*' 0.05 '.' 0.1 ' ' 1
##
## Correlation of Fixed Effects:
##            (Intr)
## conditionB -0.139
```

```r
anova(model_pre_condition_crossed, model_null)
```

```
## Data: collapse
## Models:
## model_null: rating_controlled ~ (1 | id)
## model_pre_condition_crossed: rating_controlled ~ condition + (1 | id/time)
##                             npar    AIC    BIC  logLik deviance  Chisq Df
## model_null                     3 2492.6 2504.3 -1243.3   2486.6
## model_pre_condition_crossed    5 2495.2 2514.7 -1242.6   2485.2 1.4364  2
##                             Pr(>Chisq)
## model_null
## model_pre_condition_crossed     0.4876
```

```r
# not a significant improvement

# Secondary hyperalgesia intensity is not predicted by condition.


# Assessing whether the individual calibration approach confounded the results (intensity of SH)

max_intensity <- intensity %>%
  group_by(id, condition) %>%
  summarise(max_rating = max(pp_rating, na.rm=TRUE))

max_vs_used <-merge(x=max_intensity, y=demo_info[,c(1,10)], by.x = 'id' , by.y = 'id' , all.x = TRUE)


# First check distributional assumptions for correlation.
# Shapiro-Wilk normality
with(max_vs_used, shapiro.test(Intensity_used)) # P = 0.011
```

```
##
##  Shapiro-Wilk normality test
##
## data:  Intensity_used
## W = 0.93955, p-value = 0.01074
```

```r
correlation <- cor.test(max_vs_used$max_rating, max_vs_used$Intensity_used, method = "spearman")
correlation # Not significant
```

```
##
##  Spearman's rank correlation rho
##
## data:  max_vs_used$max_rating and max_vs_used$Intensity_used
## S = 22496, p-value = 0.7799
## alternative hypothesis: true rho is not equal to 0
## sample estimates:
##        rho
## 0.03970626
```

Magnitude of secondary hyperalgesia: Assessment of model fit

```r
# The model is called model_pre_condition_crossed and has the structure: rating_controlled ~ condition

# Assumption 1: Homoscedasticity, i.e. equal variance across the range of predicted values.
plot(fitted(model_pre_condition_crossed), residuals(model_pre_condition_crossed))
```

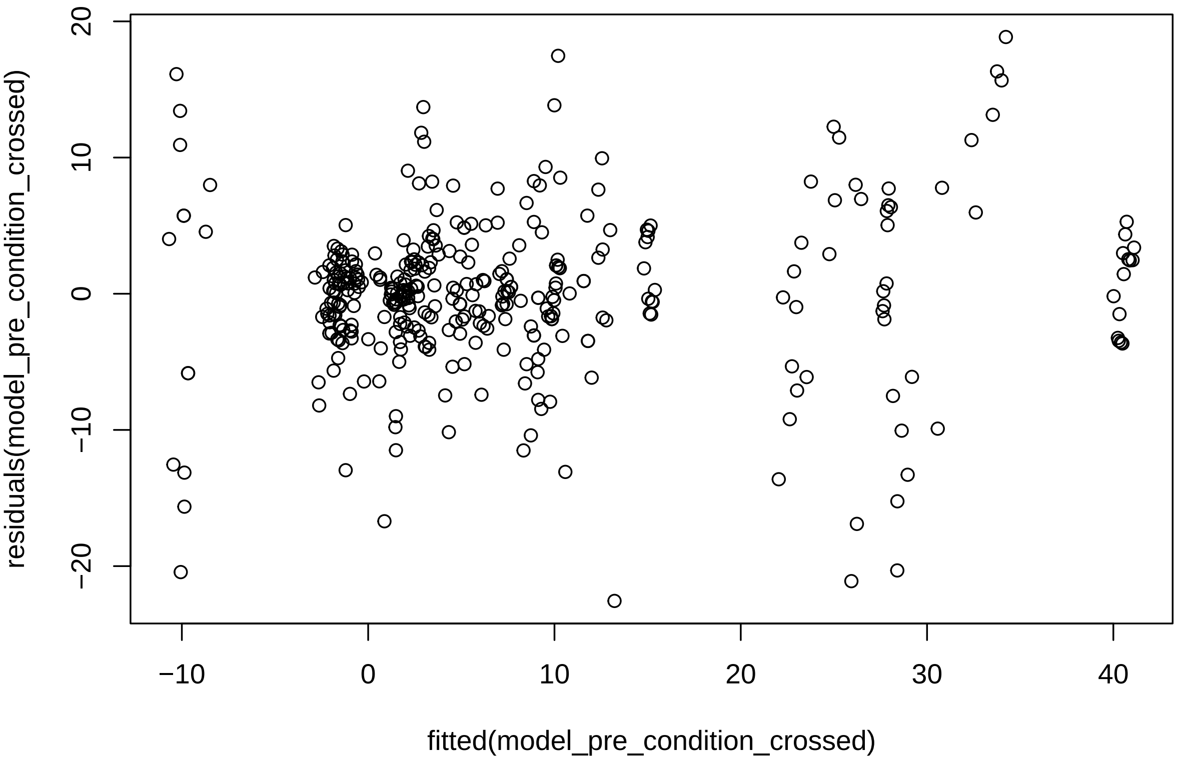


```r
# Interpretation: increased density in a blob on the left (i.e. more data points here), but the range o

# Assumption 2: Normally distributed residuals
# Plot residuals
hist(residuals(model_pre_condition_crossed))
```

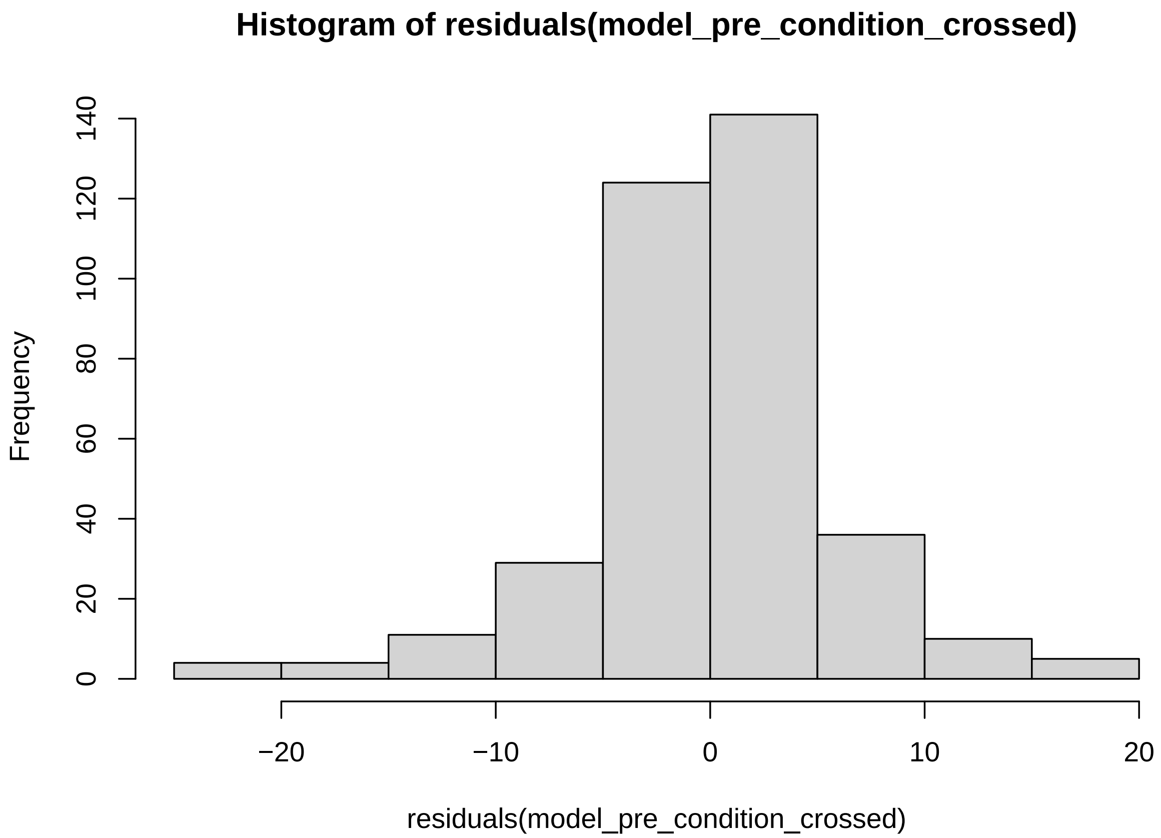


```
qqnorm(residuals(model_pre_condition_crossed))
```

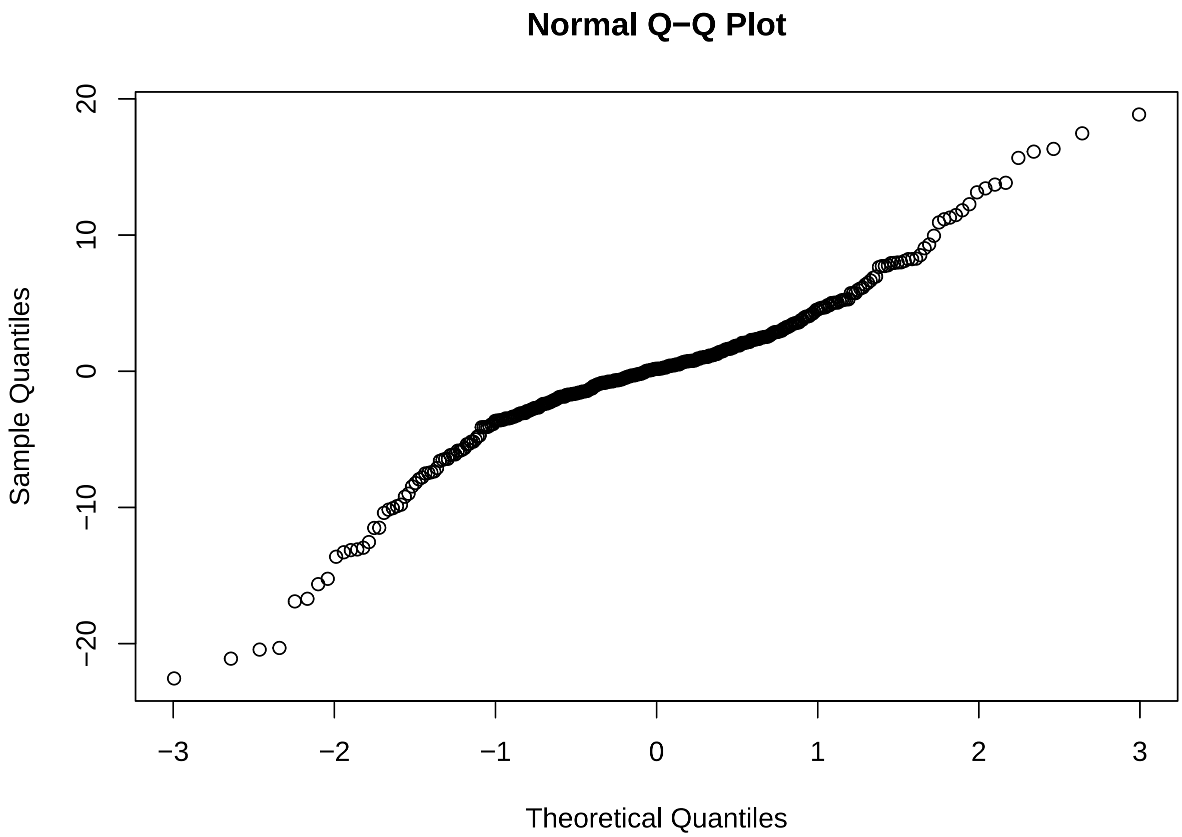


```
# Interpretation: Q-Q plot shows extremely minor deviation from the diagonal reference line. Histogram
```

Secondary outcome: surface area of secondary hyperalgesia

We are interested in determining the surface area of secondary hyperalgesia on each arm, and the comparison between arms.

```
SA <- master_data %>%
  select(group,
         id,
         time,
         phase,
         site,
         modality,
         rating) %>%
  filter(phase == 'test_sa')


SA %<>% mutate(condition = case_when(
  group == '1' & site == 'right' ~ 'A',
  group == '1' & site == 'left' ~ 'B',
  group == '2' & site == 'right' ~ 'B',
```

```
  group == '2' & site == 'left' ~ 'A')) %>%
  select(-group,
         -site,
         -phase)

# replace ND with 0

SA$rating[SA$rating == 'ND'] <- 0
names(SA)[names(SA) == 'rating'] <- 'point1'
names(SA)[names(SA) == 'modality'] <- 'radial_line'

SA %<>% group_by(id, time) %>%
  mutate(point2 = case_when(radial_line == 'A' ~ lead(point1, 5),
                            radial_line == 'B' ~ lag(point1, 2),
                            radial_line == 'C' ~ lag(point1, 2),
                            radial_line == 'D' ~ lag(point1, 2),
                            radial_line == 'E' ~ lead(point1, 7),
                            radial_line == 'F' ~ lag(point1, 2),
                            radial_line == 'G' ~ lag(point1, 2),
                            radial_line == 'H' ~ lag(point1, 2))) %>%
  ungroup()

SA$point1 <- as.numeric(SA$point1)
SA$point2 <- as.numeric(SA$point2)
# Now calculate surface area for each triangle
library(REdaS)
sinangle <- sin((deg2rad(45)))

sinangle
```

```
## [1] 0.7071068
```

```
SA %<>% mutate(triangle = (point1*point2*sinangle)/2)

# Now calculate SA for each id, site and time

SA %<>% select(-radial_line,
                     -point1,
                     -point2)

SA %<>% group_by(id,
                 time,
                 condition) %>%
  summarise(SA = sum(triangle)) %>%
  ungroup()

SA_plot <- ggplot(data = SA) +
  aes(x = as.factor(time),
      y = SA,
      colour = condition,
      group = condition) +
  geom_point() +
  geom_line(aes(colour = condition)) +
```

```
  facet_wrap(~ id,
             nrow = 2) +
  guides(colour = guide_legend("Condition"), size = guide_legend("Condition")) +
  labs(x = 'Time (minutes)',
       y = 'Surface area (cm2)',
       title = 'Individual area of SA, over time')
SA_plot
```

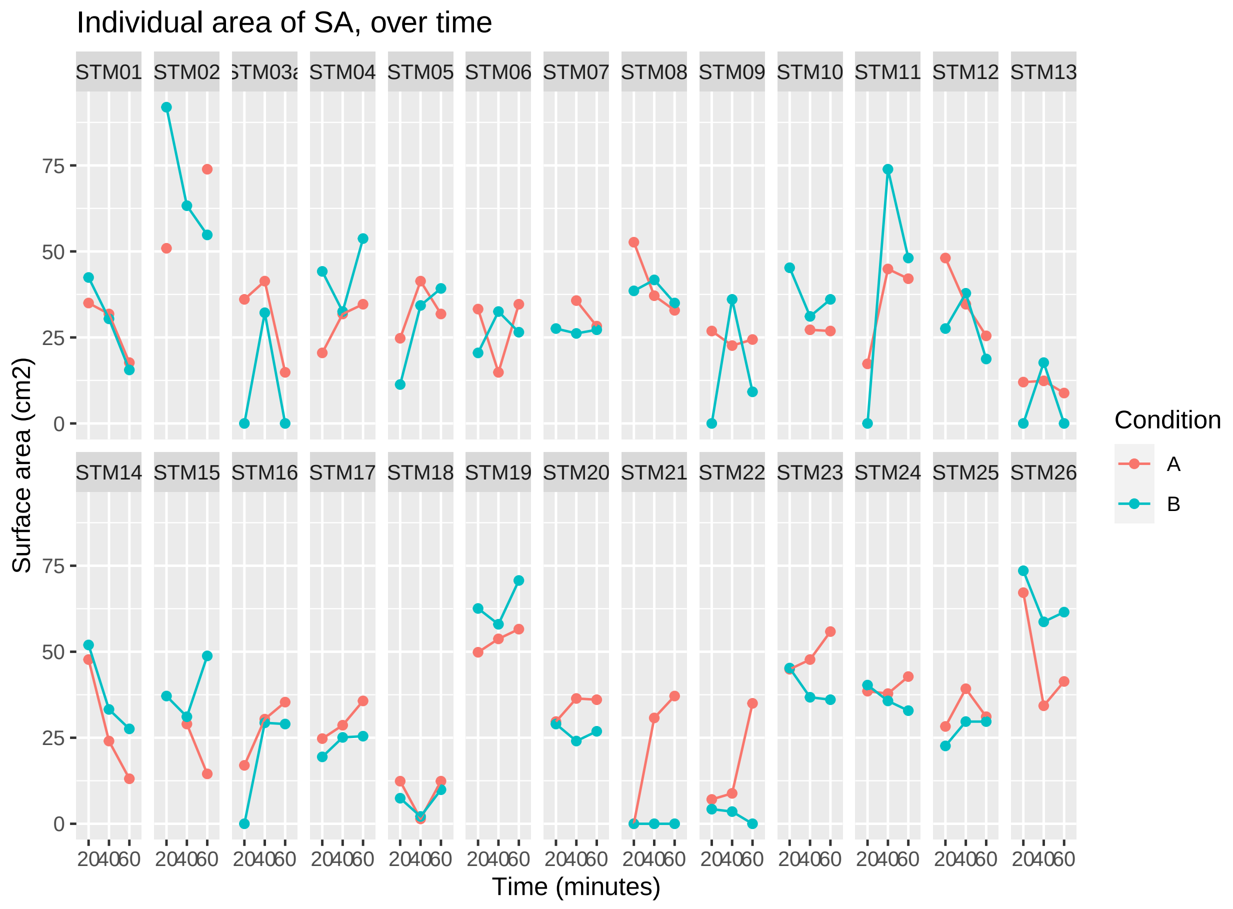


```
#remove blinding to make graph clear
SA$condition[SA$condition == 'A'] <- 'Threat site'

SA$condition[SA$condition == 'B'] <- 'Control site'


# All participants, group data represented by boxplots
ggplot(data = SA) +
  aes(x = factor(time),
      y = SA,
      group = interaction(time,condition),
      colour = condition) +
  geom_boxplot() +
  geom_point(position = position_jitterdodge()) +
```

```r
  scale_colour_manual(values = c("#009E73", "#D55E00")) +
  scale_x_discrete(breaks = seq(20,60,20)) +
  guides(colour = guide_legend("Condition"), size = guide_legend("Condition")) +
  labs(y = 'Surface area of secondary hyperalgesia (cm2)',
       x ='Time (mins)')
```

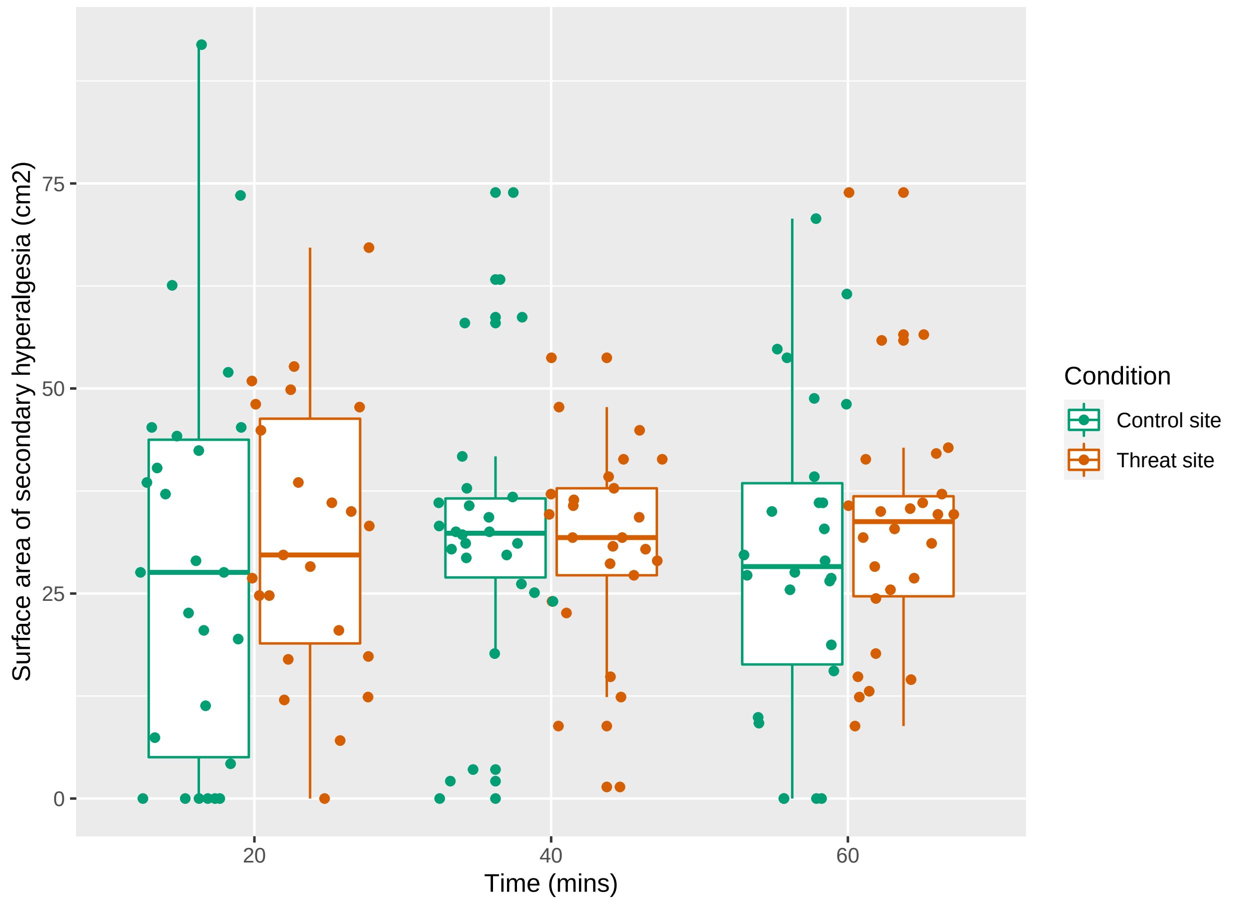


```r
# We are interested in a main effect of condition on area of secondary hyperalgesia

model_null_sa <- lmer(SA ~ (1|id),
                      data = SA)
model_condition_sa <- lmer(SA ~ condition + (1|id),
                          data = SA)
anova(model_condition_sa, model_null_sa) # Condition does not predict area of secondary hyperalgesia
```

```
## Data: SA
## Models:
## model_null_sa: SA ~ (1 | id)
## model_condition_sa: SA ~ condition + (1 | id)
##                     npar    AIC    BIC  logLik deviance  Chisq Df Pr(>Chisq)
## model_null_sa          3 1243.8 1252.9 -618.91   1237.8
## model_condition_sa     4 1244.9 1257.0 -618.44   1236.9 0.9326  1     0.3342
```

```r
model_pre_condition_crossed_sa <- lmer(SA ~ condition + (1|id/time),
                                       data = SA)

anova(model_pre_condition_crossed_sa, model_condition_sa)
```

```
## Data: SA
## Models:
## model_condition_sa: SA ~ condition + (1 | id)
## model_pre_condition_crossed_sa: SA ~ condition + (1 | id/time)
##                                npar    AIC    BIC  logLik deviance Chisq Df
## model_condition_sa                4 1244.9 1257.0 -618.44   1236.9
## model_pre_condition_crossed_sa    5 1243.2 1258.4 -616.62   1233.2 3.639  1
##                                Pr(>Chisq)
## model_condition_sa
## model_pre_condition_crossed_sa    0.05644 .
## ---
## Signif. codes:  0 '***' 0.001 '**' 0.01 '*' 0.05 '.' 0.1 ' ' 1
```

```r
# not a significant improvement

# Secondary hyperalgesia surface area is not predicted by condition.
```

Surface area of secondary hyperalgesia: Assessment of model fit

```r
# The model is called model_pre_condition_crossed_sa and has the structure: SA ~ condition + (1|id/time

# Assumption 1: Homoscedasticity, i.e. equal variance across the range of predicted values.
plot(fitted(model_pre_condition_crossed_sa), residuals(model_pre_condition_crossed_sa))
```

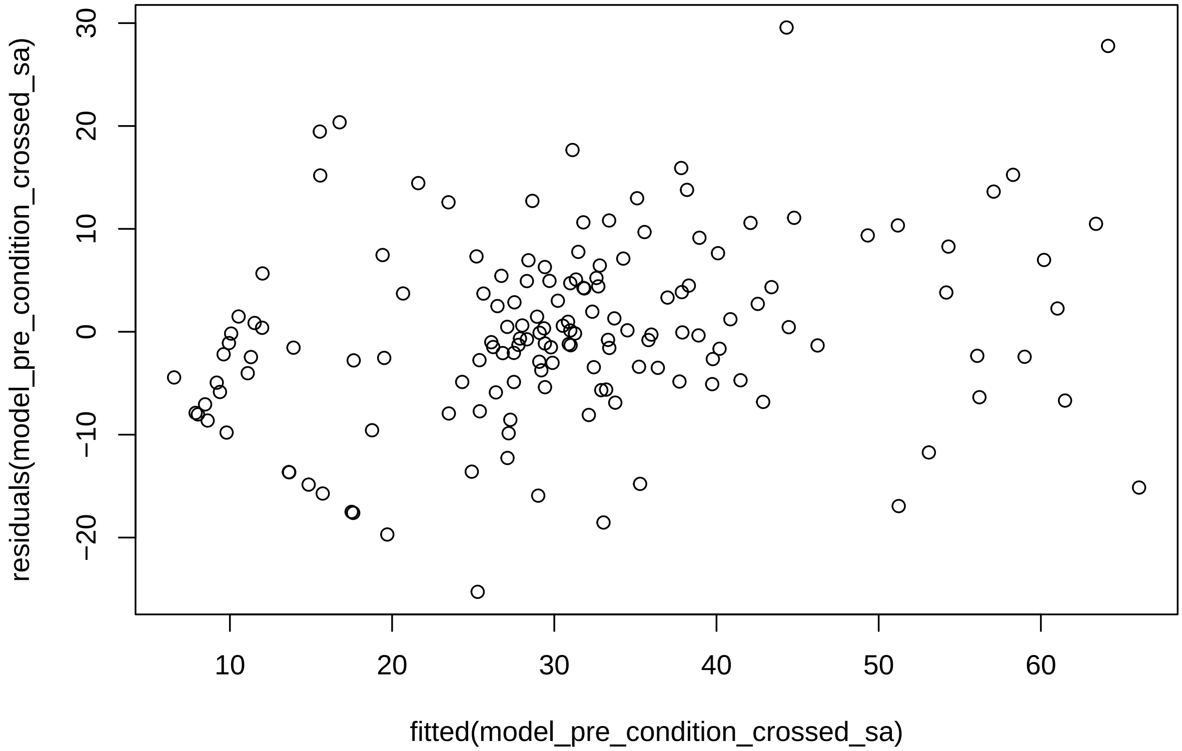


```r
## Interpretation: no clear pattern; assumption upheld.

# Assumption 2: Normally distributed residuals
# Plot residuals
hist(residuals(model_pre_condition_crossed_sa))
```

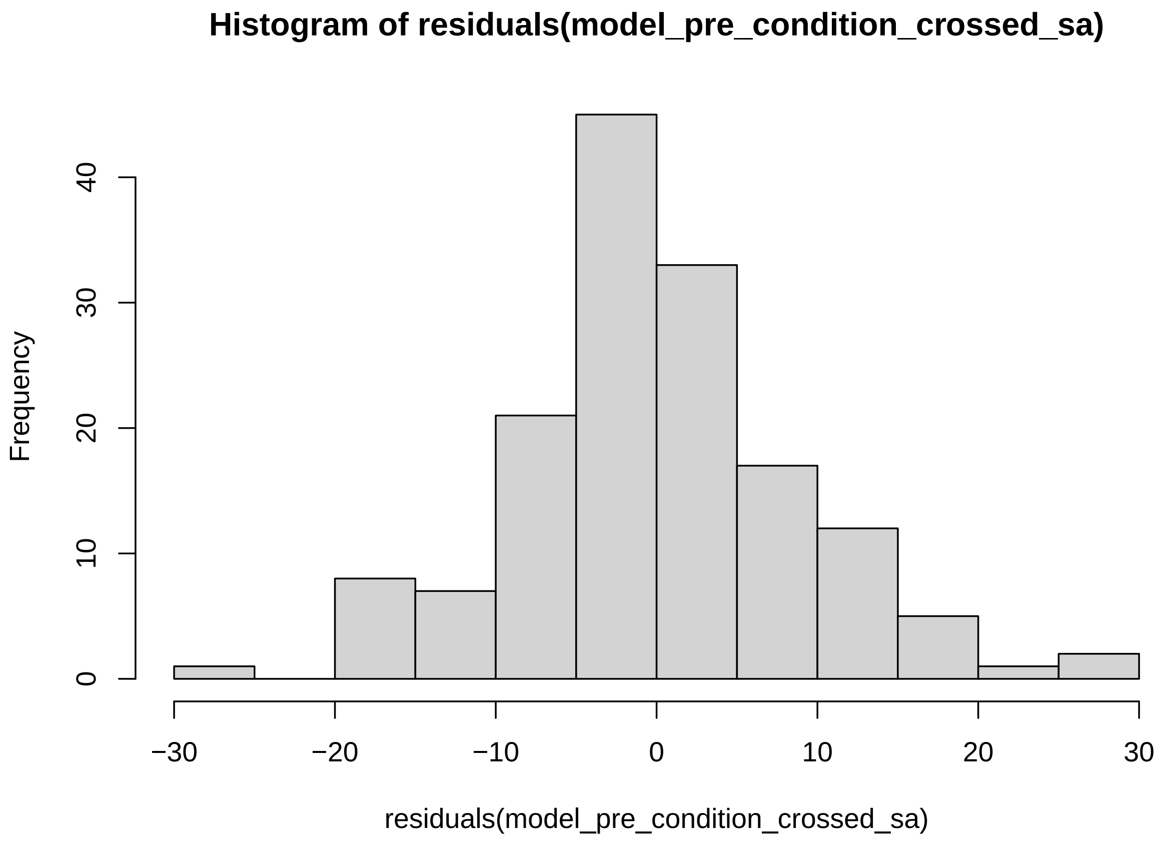


```
qqnorm(residuals(model_pre_condition_crossed_sa))
```

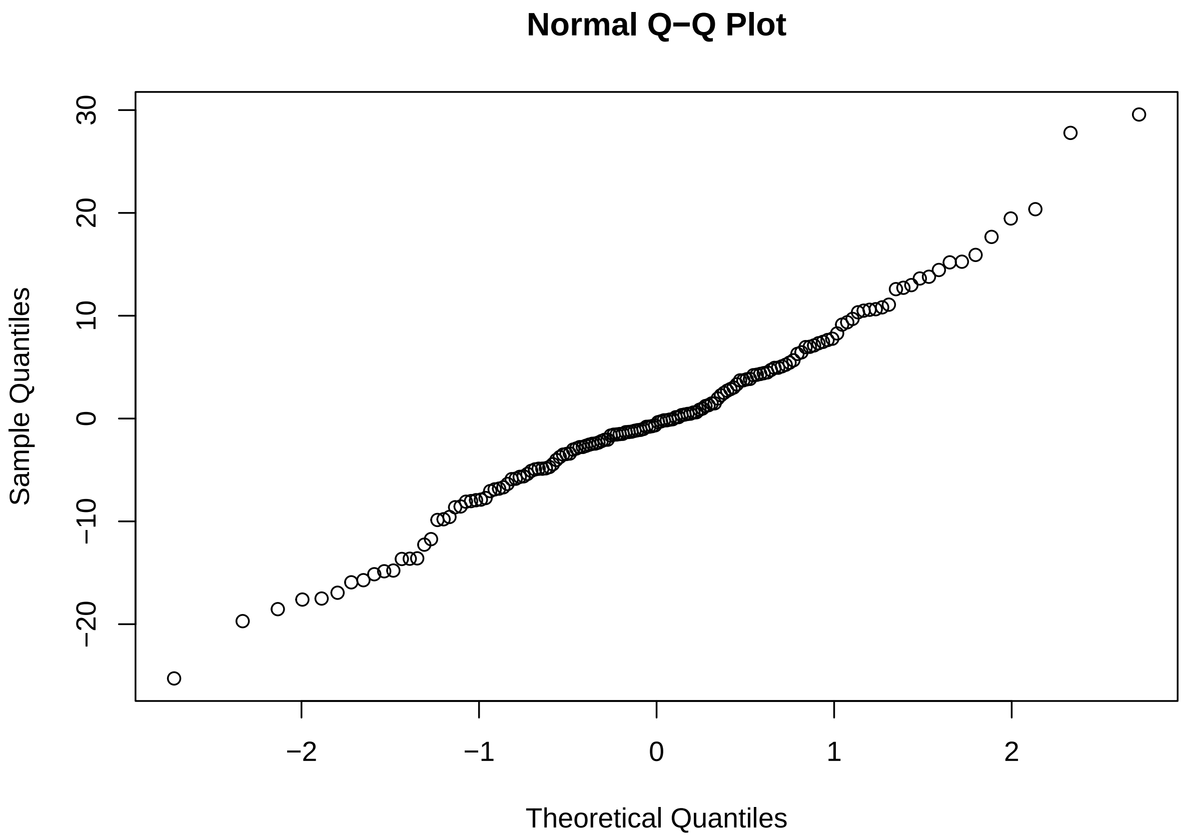


```
# Interpretation: Q-Q plot and histogram show normal distribution. Assumption was deemed to have been u
```

Childhood and recent trauma scores

Assess the relationship between trauma history and area of SH.

```
cidi <- master_data[ , grep('cidi', colnames(master_data))]
cidi$cidi_total <- rowSums(cidi)

cidi <- cbind(master_data$id, cidi)
cidi <- unique(cidi)

#extract ctq data only
ctq <- questionnaire_output_all_tab[ , grep('ctq', colnames(questionnaire_output_all_tab))]

#remove items 10, 16 and 22

ctq <- ctq %>%
  select(-ctq_10,
         -ctq_16,
         -ctq_22)
```

```r
#Now, reverse the score for items 2, 5, 7, 13, 19, 26, and 28

ctq_reverse <- ctq %>%
  select(ctq_2, ctq_5, ctq_7, ctq_13, ctq_19, ctq_26, ctq_28)

ctq_reverse %<>%
  mutate(ctq_2_reverse = case_when(
    ctq_2 == '1' ~ '5',
    ctq_2 == '2' ~ '4',
    ctq_2 == '3' ~ '3',
    ctq_2 == '4' ~ '2',
    ctq_2 == '5' ~ '1')) %>%
    mutate(ctq_5_reverse = case_when(
    ctq_5 == '1' ~ '5',
    ctq_5 == '2' ~ '4',
    ctq_5 == '3' ~ '3',
    ctq_5 == '4' ~ '2',
    ctq_5 == '5' ~ '1')) %>%
  mutate(ctq_7_reverse = case_when(
    ctq_7 == '1' ~ '5',
    ctq_7 == '2' ~ '4',
    ctq_7 == '3' ~ '3',
    ctq_7 == '4' ~ '2',
    ctq_7 == '5' ~ '1')) %>%
  mutate(ctq_13_reverse = case_when(
    ctq_13 == '1' ~ '5',
    ctq_13 == '2' ~ '4',
    ctq_13 == '3' ~ '3',
    ctq_13 == '4' ~ '2',
    ctq_13 == '5' ~ '1')) %>%
  mutate(ctq_19_reverse = case_when(
    ctq_19 == '1' ~ '5',
    ctq_19 == '2' ~ '4',
    ctq_19 == '3' ~ '3',
    ctq_19 == '4' ~ '2',
    ctq_19 == '5' ~ '1')) %>%
  mutate(ctq_26_reverse = case_when(
    ctq_26 == '1' ~ '5',
    ctq_26 == '2' ~ '4',
    ctq_26 == '3' ~ '3',
    ctq_26 == '4' ~ '2',
    ctq_26 == '5' ~ '1')) %>%
  mutate(ctq_28_reverse = case_when(
    ctq_28 == '1' ~ '5',
    ctq_28 == '2' ~ '4',
    ctq_28 == '3' ~ '3',
    ctq_28 == '4' ~ '2',
    ctq_28 == '5' ~ '1'))

#Now remove the original scores that had not be reversed

ctq_reverse %<>%
  select(ctq_2_reverse, ctq_5_reverse, ctq_7_reverse, ctq_13_reverse, ctq_19_reverse, ctq_26_reverse, c
```

```
ctq_reverse$ctq_2_reverse <- as.numeric(ctq_reverse$ctq_2_reverse)
ctq_reverse$ctq_5_reverse <- as.numeric(ctq_reverse$ctq_5_reverse)
ctq_reverse$ctq_7_reverse <- as.numeric(ctq_reverse$ctq_7_reverse)
ctq_reverse$ctq_13_reverse <- as.numeric(ctq_reverse$ctq_13_reverse)
ctq_reverse$ctq_19_reverse <- as.numeric(ctq_reverse$ctq_19_reverse)
ctq_reverse$ctq_26_reverse <- as.numeric(ctq_reverse$ctq_26_reverse)
ctq_reverse$ctq_28_reverse <- as.numeric(ctq_reverse$ctq_28_reverse)

#Now join ctq_reverse with the remaining questions that did not need to be reversed

ctq_normal <- ctq %>%
  select(-ctq_2, -ctq_5, -ctq_7, -ctq_13, -ctq_19, -ctq_26, -ctq_28)

ctq_all <- cbind(ctq_normal, ctq_reverse)

#Sum the rows

ctq_all$ctq_total <- rowSums(ctq_all, na.rm = TRUE)

CA_all_with_pid <- cbind(questionnaire_output_all_tab$id...1, ctq_all)
names(CA_all_with_pid)[names(CA_all_with_pid) == 'questionnaire_output_all_tab$id...1'] <- 'id'


trauma <- cbind(cidi, CA_all_with_pid)
trauma %<>% mutate(trauma = cidi_total + ctq_total) %>%
  select(id,
         trauma)


SA_trauma <- SA %>% right_join(trauma)

# We need to identify the best time point from which to draw the SA data for each participant. We will

# Considering that secondary hyperalgesia needs first to be verified before the surface area becomes a

intensity_time <- intensity %>% group_by(id, time) %>%
  summarise(mean_int = mean(pp_rating)) %>%
  filter(time != 0) %>%
  ungroup() %>%
  group_by(id) %>%
  filter(mean_int == max(mean_int))

# Now identify SA at those time point, using the df 'intensity_time'. Adjust values to closest applicab

SA_at_peak <- SA_trauma %>%
  filter(id == 'STM01' & time == 20 |
           id == 'STM02' & time == 40 |
           id == 'STM03a' & time == 60 |
           id == 'STM04' & time == 20 |
           id == 'STM05' & time == 40 |
           id == 'STM06' & time == 60 |
           id == 'STM07' & time == 40 |
           id == 'STM08' & time == 60 |
```

```
           id == 'STM09' & time == 60 |
           id == 'STM10' & time == 20 |
           id == 'STM11' & time == 60 |
           id == 'STM12' & time == 60 |
           id == 'STM13' & time == 60 |
           id == 'STM14' & time == 20 |
           id == 'STM15' & time == 20 |
           id == 'STM16' & time == 40 |
           id == 'STM17' & time == 20 |
           id == 'STM18' & time == 60 |
           id == 'STM19' & time == 20 |
           id == 'STM20' & time == 40 |
           id == 'STM21' & time == 20 |
           id == 'STM22' & time == 40 |
           id == 'STM23' & time == 40 |
           id == 'STM24' & time == 40 |
           id == 'STM25' & time == 60 |
           id == 'STM26' & time == 20 ) # for STM20 the mean_int was the same at time points 38 and 50.


names(SA_at_peak)[names(SA_at_peak) == 'SA'] <- 'peak_SA'

ggplot(data = SA_at_peak) +
  aes(x = trauma,
      y = peak_SA) +
  geom_point() +
  geom_smooth(method = 'lm')+
  labs(title = 'Individual peak SA, over summed trauma scores',
       y = 'Peak SA (cm2)',
       x = 'Summed trauma scores')
```

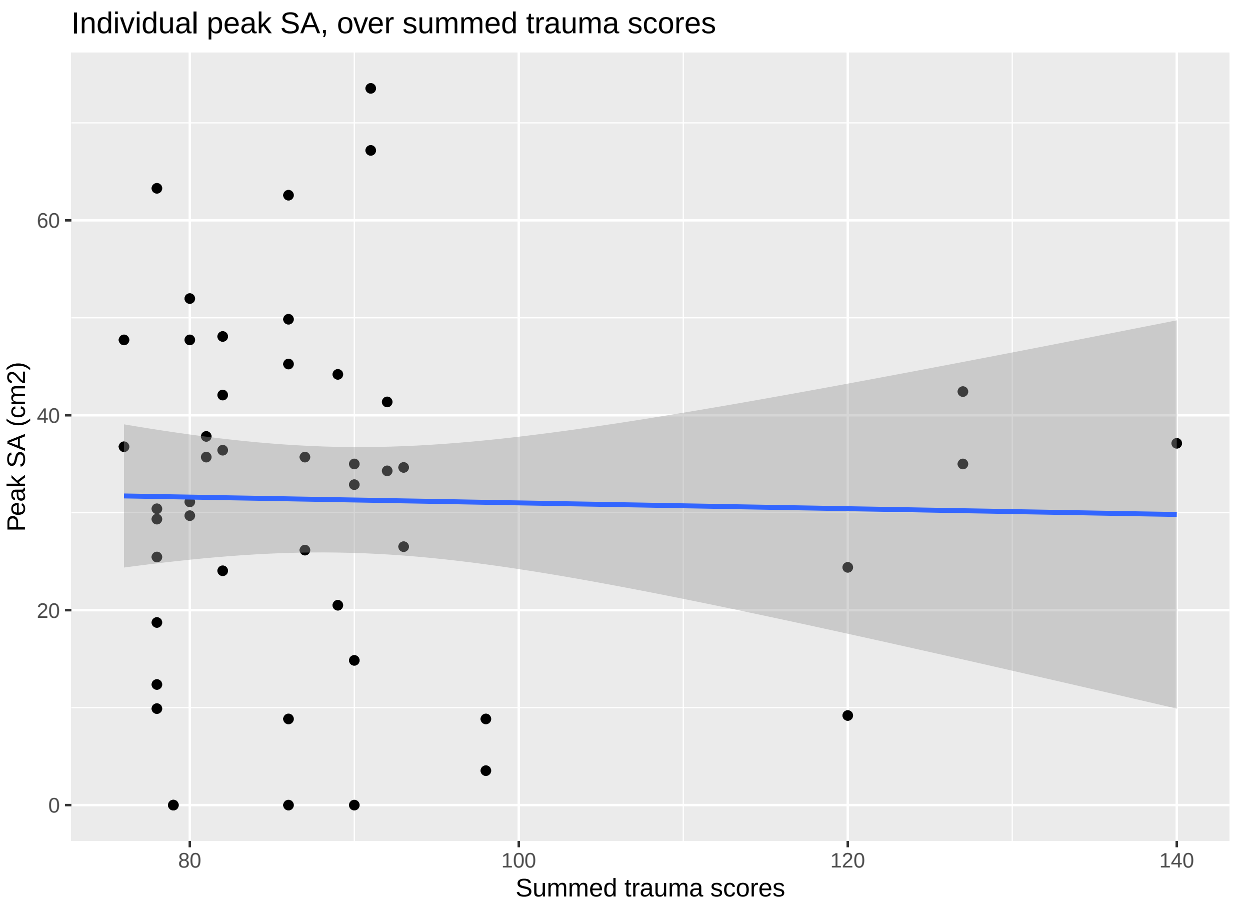


```r
# First check distributional assumptions for correlation.
# Shapiro-Wilk normality test for peak surface area
with(SA_at_peak, shapiro.test(peak_SA)) # P = 0.483
```

```
##
##  Shapiro-Wilk normality test
##
## data:  peak_SA
## W = 0.9738, p-value = 0.3406
```

```r
# Pearsons correlation

correlation <- cor.test(SA_at_peak$trauma, SA_at_peak$peak_SA, method= "pearson")
correlation # P = 0.307 therefore no significant relationship between summed trauma scores and peak SA.
```

```
##
##  Pearson's product-moment correlation
##
## data:  SA_at_peak$trauma and SA_at_peak$peak_SA
## t = -0.15885, df = 45, p-value = 0.8745
## alternative hypothesis: true correlation is not equal to 0
## 95 percent confidence interval:
##  -0.3087412  0.2652970
```

```
## sample estimates:
##         cor
## -0.02367332
```

#Post-hoc analyses Post-hoc analyses were performed on the mean (range) of the Pain Catastrophising Scale and the 10-item Connor-Davidson Resilience Scale. These analyses were not initially planned nor described in this study's protocol. These analyses were performed for exploratory purposes to inform the development of future research questions.

```
#PCS

 PCS_results_only <- read_delim('C:/Users/Gill/Desktop/Formal data analysis Jan 2020/Gill_formal_data_HI
    ';', escape_double = FALSE, trim_ws = TRUE)

pcs <- PCS_results_only[-c(1,2),] # removing participants STM01 and STM02 because they are missing data

pcs_range <- pcs %>%
  select(-SUBJ_ID)

pcs_range$pcs_total <- rowSums(pcs_range)

#Determining the overall mean PCS scores among the 24 participants
mean(pcs_range$pcs_total)
```

```
## [1] 31
```

```
#Determining the range
range(pcs_range$pcs_total)
```

```
## [1] 14 50
```

```
#Post-hoc: CD-RISC-10

cdrisc <- master_data[ , grep('cdrisc', colnames(master_data))]

cdrisc<- unique(cdrisc)

cdrisc$cdrisc_total <- rowSums(cdrisc)
# Determining the mean
mean(cdrisc$cdrisc_total)
```

```
## [1] 40.80769
```

```
sd(cdrisc$cdrisc_total)
```

```
## [1] 4.534483
```

```
#Determining the range
range(cdrisc$cdrisc_total, na.rm = TRUE)
```

```
## [1] 32 48
```

Order effect

Assess the effect of order -i.e. between-group difference in differential effect when participants are grouped by order, with those who underwent threat first as one group, and those who underwent threat second in the second group. We anticipate a greater differential effect in those who underwent threat second. Alternatively, the second group could also have generalized safety to the second induction.

```
# Order effect: HFS

group_alloc <- master_data %>% select(id,
                                      Group_allocation) %>%
  unique()

order_effect_hfs <- hfs %>% right_join(group_alloc)

#Second, plot ratings on a graph

order_effect_hfs$rating <- as.numeric(order_effect_hfs$rating)

order_effect_hfs %<>% group_by(id, condition) %>%
  mutate(train = row_number()) %>%
  ungroup()

ggplot(data = order_effect_hfs) +
  aes(x = condition,
      y = rating,
      colour = condition) +
  geom_boxplot() +
  geom_point() +
  facet_grid(~ Group_allocation) +
  geom_line() +
  guides(colour = guide_legend("ID"), size = guide_legend("ID")) +
  labs( y = 'HFS Ratings',
        x = 'Condition',
        title = 'Individual SPARS ratings during each HFS train')
```

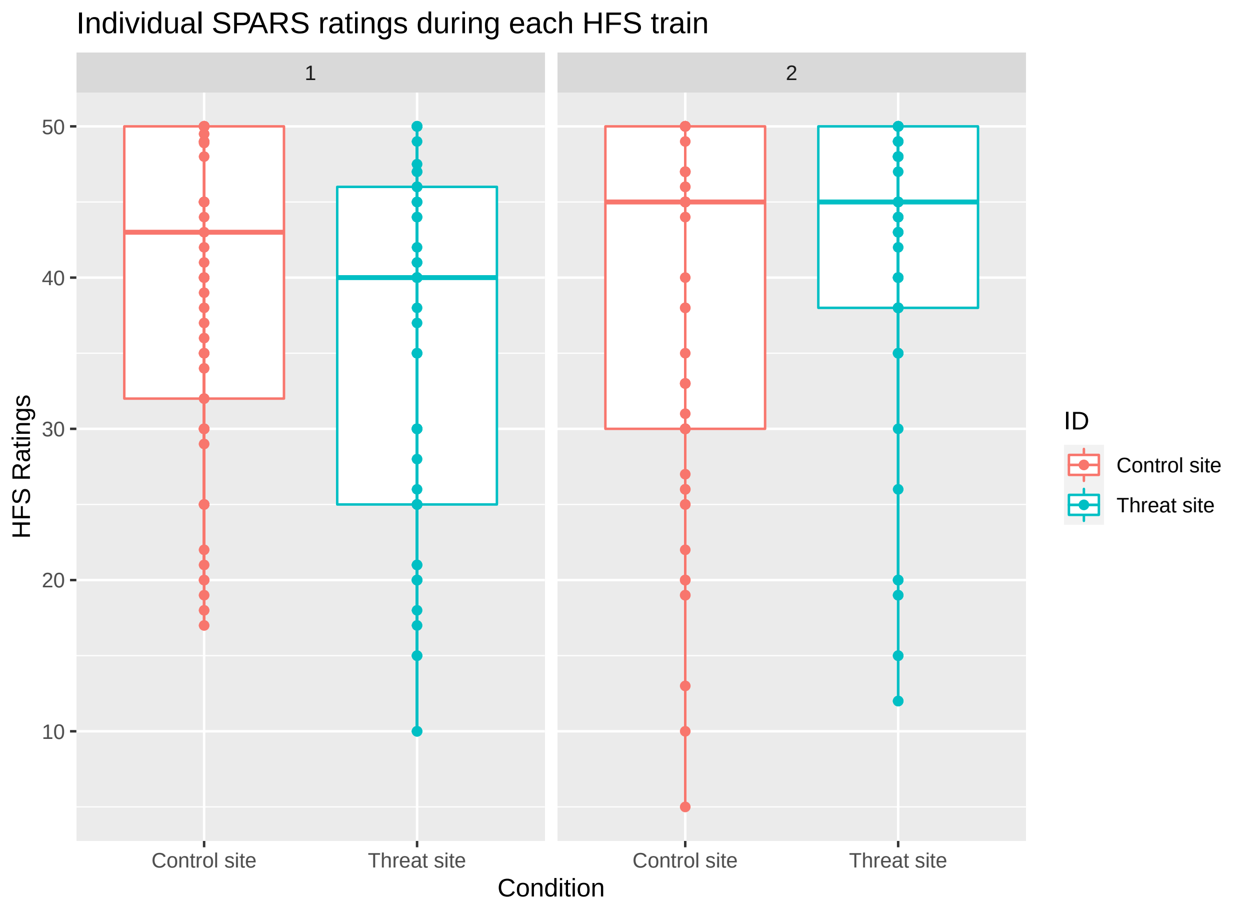


```r
# No significant order effect with HFS ratings

# Order effect: anxiety ratings

group_alloc <- master_data %>% select(id,
                                      Group_allocation) %>%
  unique()

order_effect_anxiety <- anxiety %>% right_join(group_alloc)


ggplot(data = order_effect_anxiety) +
  aes(x = condition,
      y = anxiety,
      colour = condition ) +
  geom_boxplot() +
  facet_grid(~ Group_allocation) +
  geom_line() +
  guides(colour = guide_legend("ID"), size = guide_legend("ID")) +
  labs( y = 'Anxiety ratings',
        x = 'Condition',

              title = 'Individual anxiety ratings during each HFS train')
```

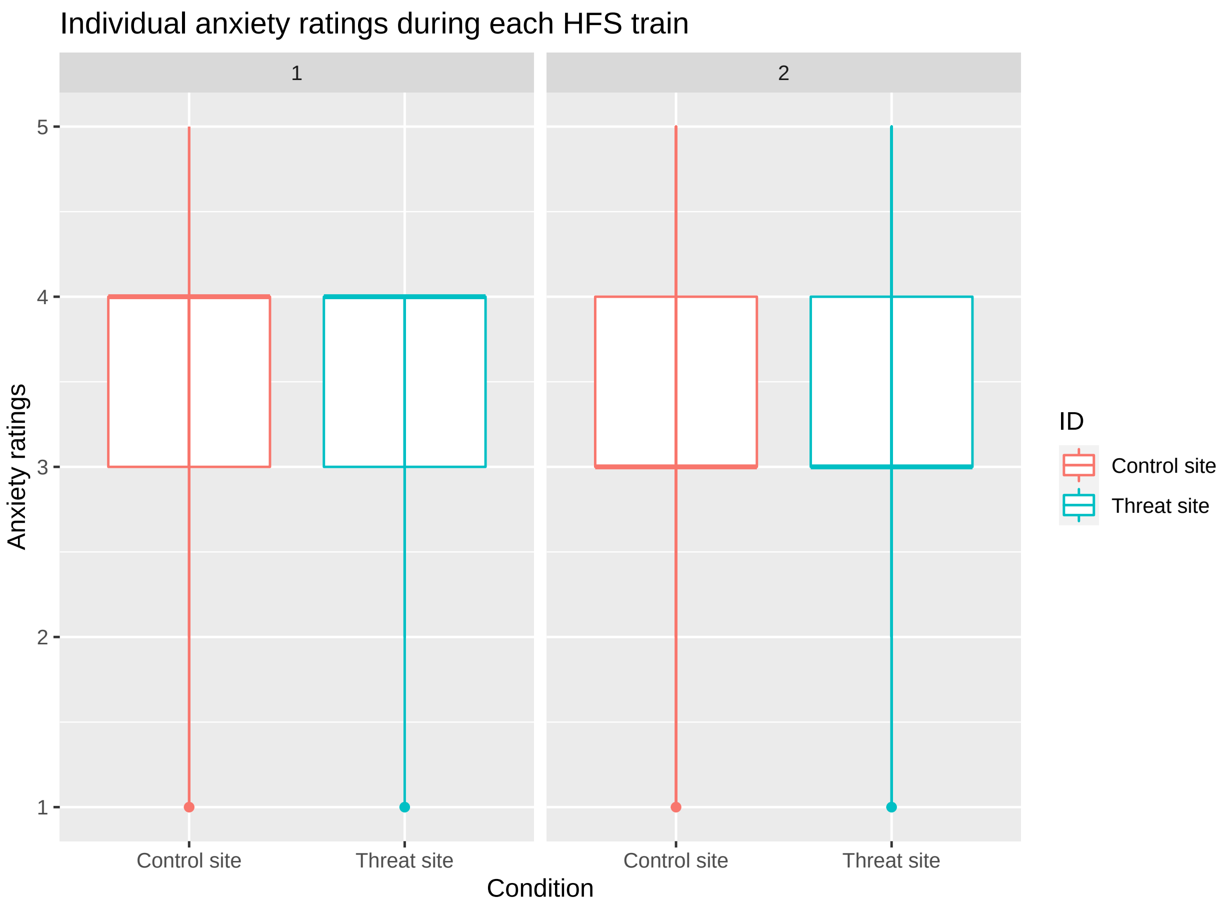


```
# Order effect: threat ratings

group_alloc <- master_data %>% select(id,
                                      Group_allocation) %>%
  unique()

order_effect_threat <- threat %>% right_join(group_alloc)


ggplot(data = order_effect_threat) +
  aes(x = condition,
      y = threat,
      colour = condition ) +
  geom_boxplot() +
  facet_grid(~ Group_allocation) +
  geom_line() +
  guides(colour = guide_legend("ID"), size = guide_legend("ID")) +
  labs( y = 'Threat ratings',
        x = 'Condtion' ,
        title = 'Individual threat ratings during each HFS train')
```

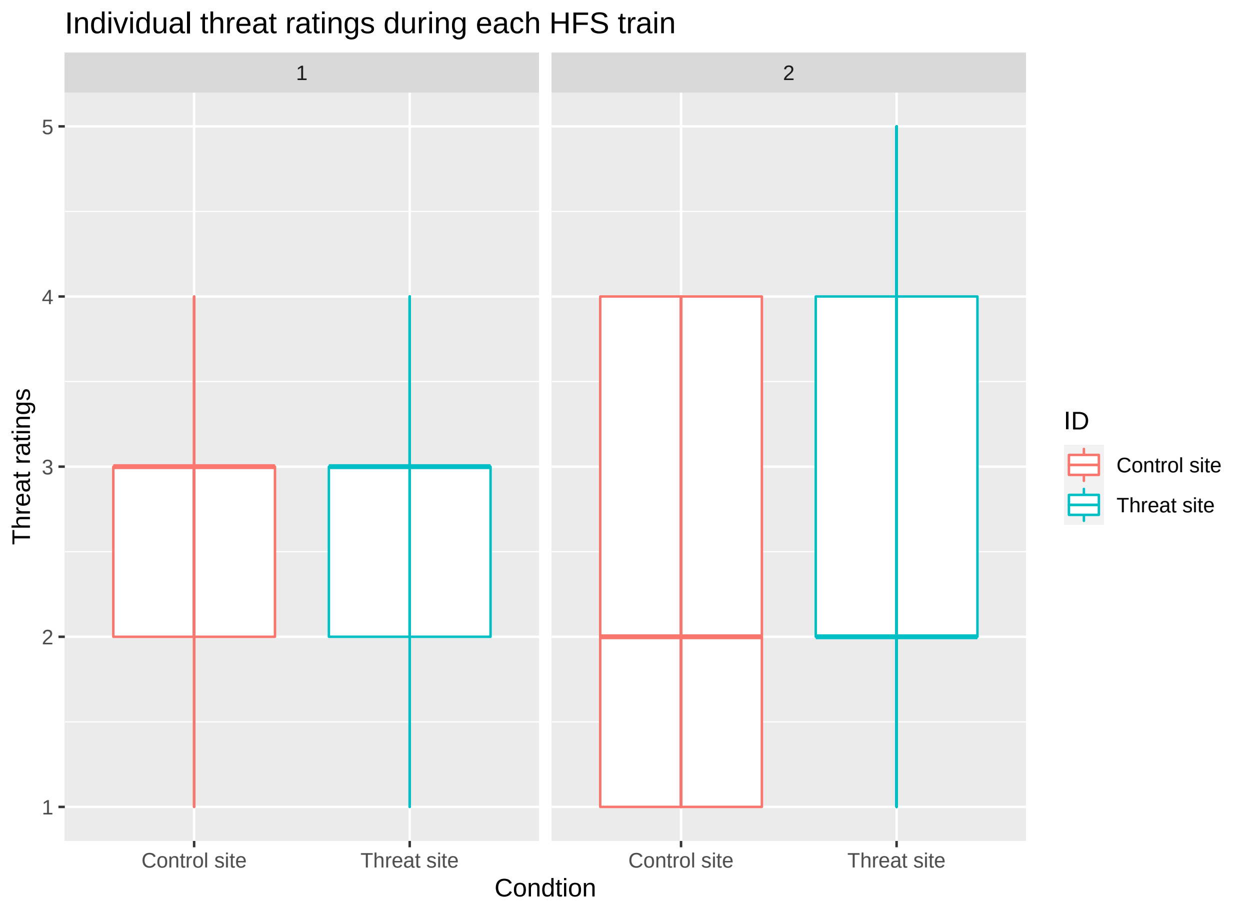

Exploratory outcomes:

Static light touch

We are interested in determining the magnitude of static light touch on each arm, and the comparison between arms.

```
slt <- master_data %>%
  select(group,
         id,
         time,
         phase,
         site,
         modality,
         rating) %>%
  filter(modality == 'VFF') %>%
  filter(phase != 'orientation') %>%
  mutate(time = if_else(phase == 'baseline', time*-1L, time))

slt %<>% mutate(condition = case_when(
  group == '1' & site == 'right' ~ 'A',
  group == '1' & site == 'left' ~ 'B',
```

```
  group == '2' & site == 'right' ~ 'B',
  group == '2' & site == 'left' ~ 'A')) %>%
  select(-group,
         -site)
slt$rating <- as.numeric(slt$rating)

slt_bl <- slt %>% filter(phase == 'baseline') %>%
  group_by(id, condition) %>%
  summarise(rating = mean(rating)) %>%
  ungroup() %>%
  mutate(time = 0) %>% ungroup()

slt_fu <- slt %>% filter(phase != 'baseline') %>% ungroup () %>%
  select(-phase,
         -modality)

slt <- rbind(slt_bl, slt_fu)

slt_wide <- slt %>%
  group_by(id) %>%
  spread(key = condition,
         value = rating)

#Determining the mean (SD) slt rating per condition
mean(slt_wide$A, na.rm = TRUE)
```

```
## [1] -29.26619
```

```
sd(slt_wide$A, na.rm = TRUE)
```

```
## [1] 20.4084
```

```
mean(slt_wide$B, na.rm = TRUE)
```

```
## [1] -29.58072
```

```
sd(slt_wide$B, na.rm = TRUE)
```

```
## [1] 20.78741
```

```
# For interest, plot the pilot data

ggplot(data = slt) +
  aes(x = time,
      y = rating,
      group = condition,
      colour = condition) +
  facet_wrap(~ id) +
  geom_point() +
  geom_line() +
```

```r
  guides(colour = guide_legend("Condition"), size = guide_legend("Condition")) +
  labs(title = 'Individual static light touch ratings by condition, over time',
       y = 'Rating',
       x = 'Time (minutes)')
```

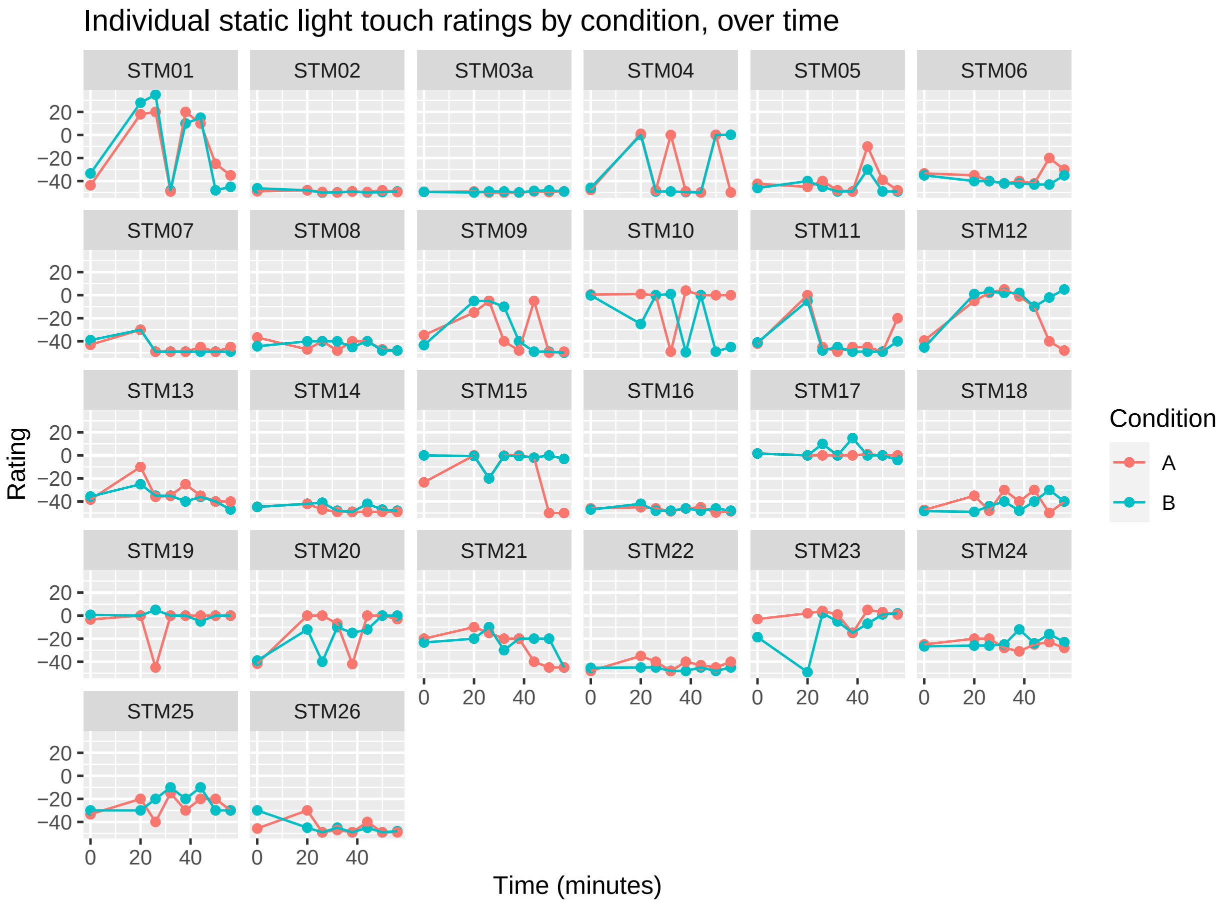


```r
# All participants, group data represented by boxplots
ggplot(data = slt) +
  aes(x = time,
      y = rating,
      group = interaction(condition, time),
      colour = condition) +
  geom_boxplot(width = 4) +
   geom_jitter(width = 0.2) + # Same solution as before
  scale_colour_manual(values = c("#009E73", "#D55E00")) +
  guides(colour = guide_legend("Condition"), size = guide_legend("Condition")) +
  labs(title = 'Group data: static light touch ratings by condition, over time N = 26',
       y = 'SPARS rating',
       x = 'Time (minutes)')
```

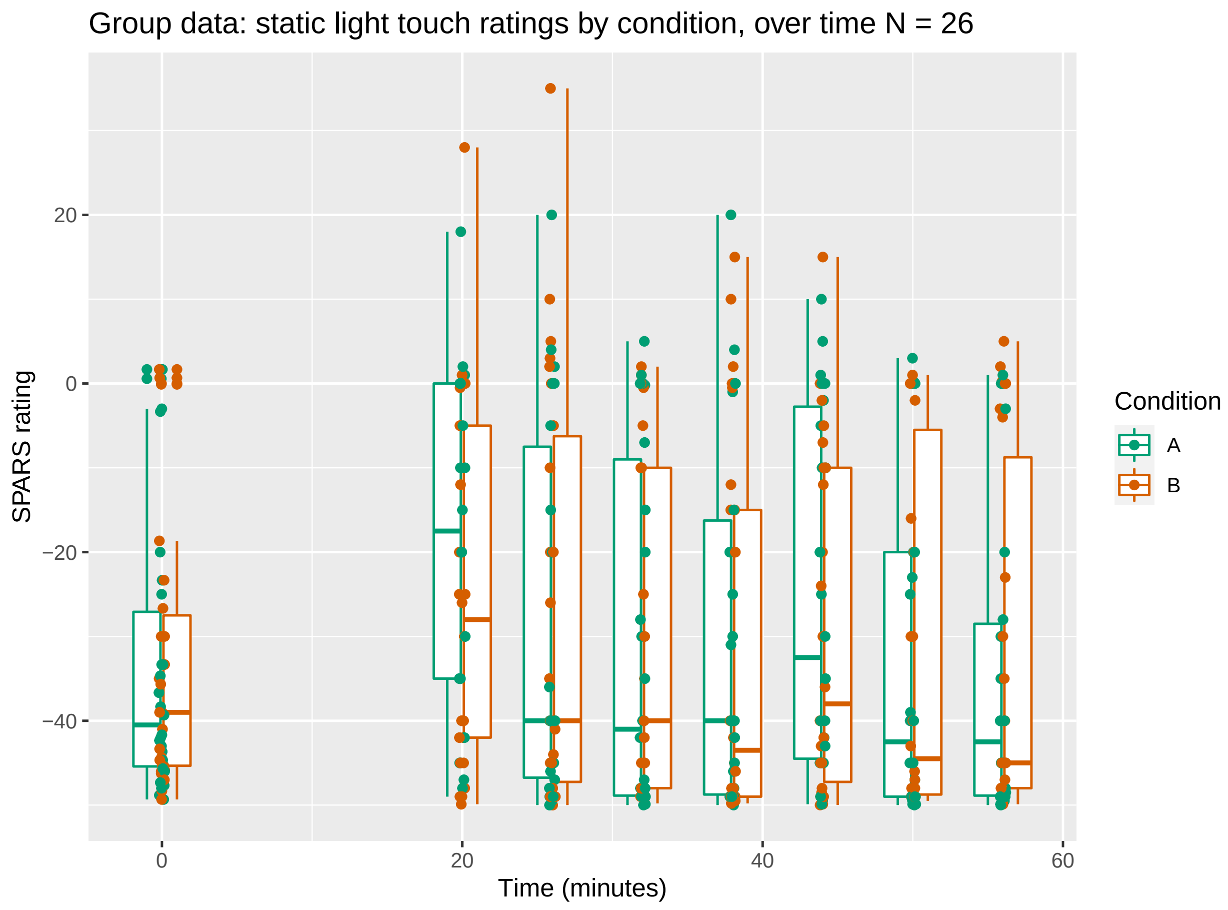


```
 # Calculate diff between conditions for group boxplots

slt_wide <- slt %>%
  group_by(id,
           time) %>%
  spread(key = condition,
         value = rating) %>%
  mutate(site_diff = A-B)

# All participants, group data represented by boxplots
ggplot(data = slt_wide) +
  aes(x = time,
      y = site_diff,
      group = time) +
  geom_boxplot(width = 2) +
  geom_point(position = 'jitter') +
  geom_hline(yintercept = 0) +
  labs(title = 'Grouped data: static light touch ratings difference between conditions N = 26',
       y = 'Static light touch ratings of condition A minus rating of condition B',
       x = 'Time (minutes)')
```

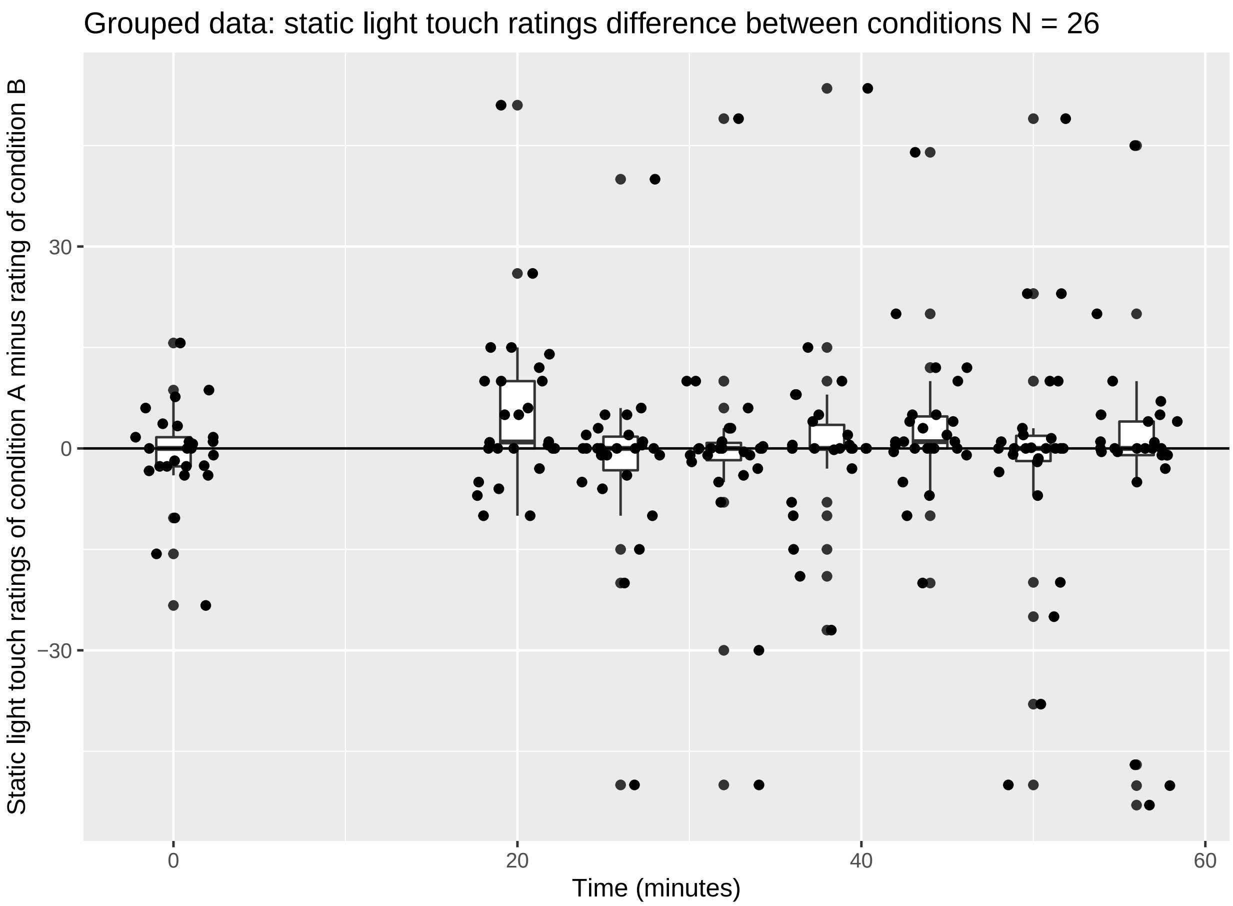


```
# Collapse all post-baseline ratings for each condition and then compare them using a RM ANOVA type mod

slt_bl %<>% select(-time)
names(slt_bl)[names(slt_bl) == 'rating'] <- 'baseline_rating'
collapse_slt <- slt_fu %>% # resolve each rating relative to baseline
  right_join(slt_bl) %>%
  mutate(rating_controlled = rating-baseline_rating) %>%
  select(id,
         time,
         condition,
         rating_controlled) # spread to calculate difference between conditions
```

Dynamic light touch

We are interested in determining the magnitude of dynamic light touch on each arm, and the comparison between arms.

```
dlt <- master_data %>%
  select(group,
         id,
         time,
         phase,
         site,
```

```
         modality,
         rating) %>%
  filter(modality == 'CW' | modality == 'BR') %>%
  filter(phase != 'orientation') %>%
  mutate(time = if_else(phase == 'baseline', time*-1L, time))

dlt %<>% mutate(condition = case_when(
  group == '1' & site == 'right' ~ 'A',
  group == '1' & site == 'left' ~ 'B',
  group == '2' & site == 'right' ~ 'B',
  group == '2' & site == 'left' ~ 'A')) %>%
  select(-group,
         -site)
dlt$rating <- as.numeric(dlt$rating)

dlt_bl <- dlt %>% filter(phase == 'baseline') %>%
  group_by(id, condition) %>%
  summarise(dlt_rating = mean(rating)) %>%
  ungroup() %>%
  mutate(time = 0) %>% ungroup()

dlt_fu <- dlt %>% filter(phase != 'baseline') %>% ungroup() %>%
  group_by(id,
           time,
           condition) %>%
  summarise(dlt_rating = mean(rating)) %>%
  ungroup()

dlt <- rbind(dlt_bl, dlt_fu)


dlt_wide <- dlt %>%
  group_by(id) %>%
  spread(key = condition,
         value = dlt_rating)

#Determining the mean (SD) dlt rating per condition
mean(dlt_wide$A, na.rm = TRUE)
```

```
## [1] -41.70841
```

```
sd(dlt_wide$A, na.rm = TRUE)
```

```
## [1] 8.393769
```

```
mean(dlt_wide$B, na.rm = TRUE)
```

```
## [1] -41.41579
```

```
sd(dlt_wide$B, na.rm = TRUE)
```

```
## [1] 8.308582
```

```r
# For interest, plot the data

ggplot(data = dlt) +
  aes(x = time,
      y = dlt_rating,
      group = condition,
      colour = condition) +
  facet_wrap(~ id) +
  geom_point() +
  geom_line() +
  guides(colour = guide_legend("Condition"), size = guide_legend("Condition")) +
  labs(title = 'Individual dynamic light touch ratings by condition, over time',
       y = 'Rating',
       x = 'Time (minutes)')
```

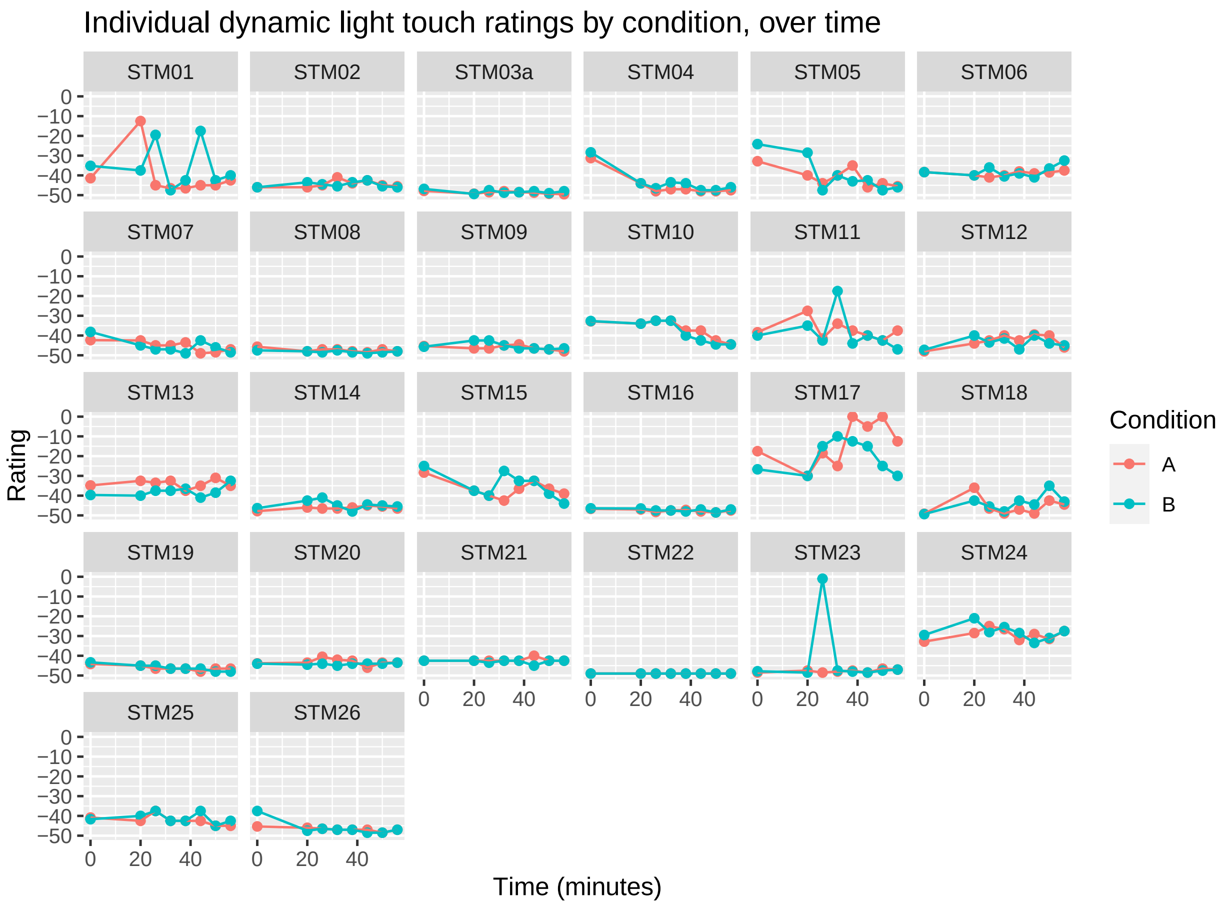


```r
# All participants, group data represented by boxplots
ggplot(data = dlt) +
  aes(x = time,
      y = dlt_rating,
      group = interaction(condition, time),
      colour = condition) +
  geom_boxplot(width = 4) +
```

```
  geom_jitter(width = 0.2) + # Same solution as before
  scale_colour_manual(values = c("#009E73", "#D55E00")) +
  guides(colour = guide_legend("Condition"), size = guide_legend("Condition")) +
  labs(title = 'Group data: dynamic light touch ratings by condition, over time N = 26',
       y = 'SPARS rating',
       x = 'Time (minutes)')
```

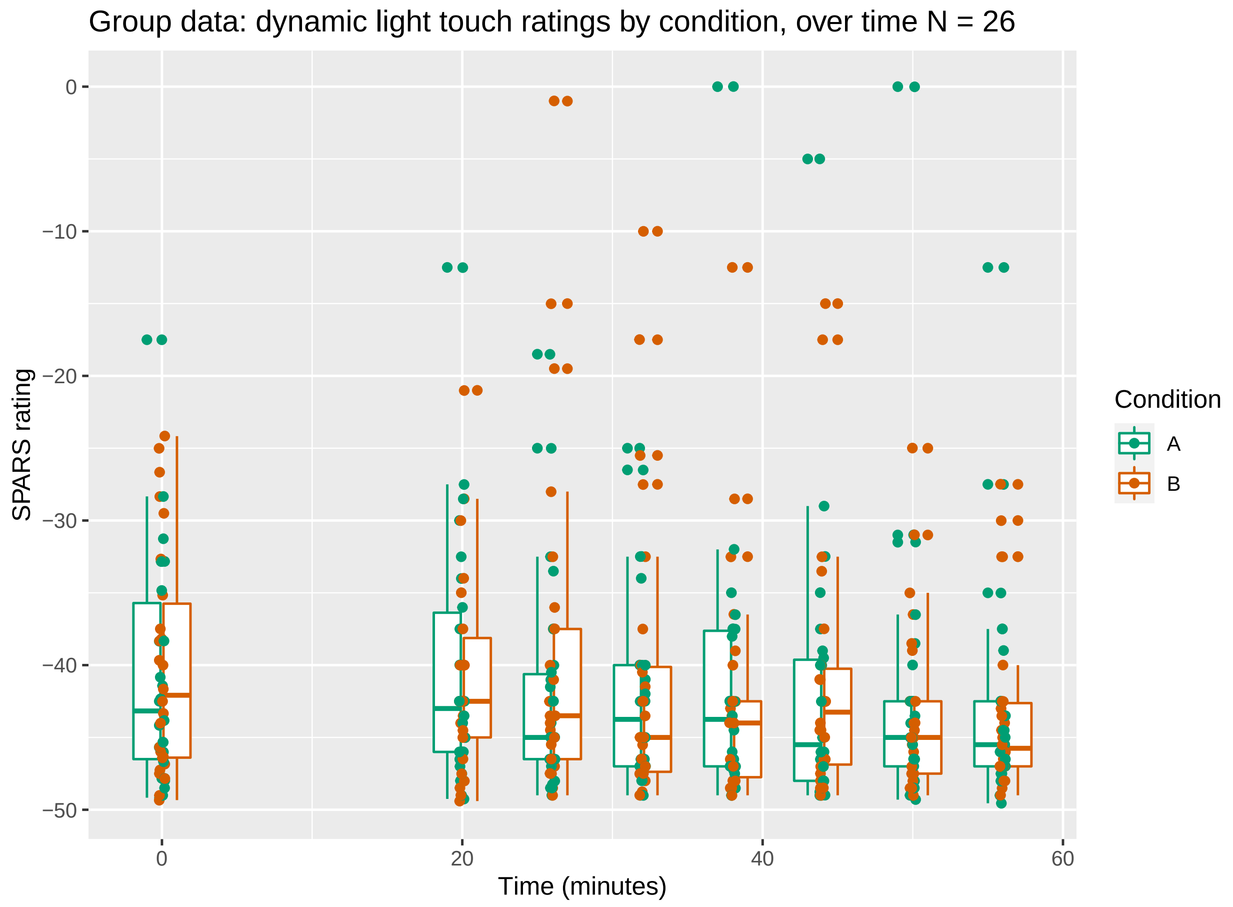


```
 # Calculate diff between conditions for group boxplots

dlt_wide <- dlt %>%
  group_by(id,
           time) %>%
  spread(key = condition,
         value = dlt_rating) %>%
  mutate(site_diff = A-B)

# All participants, group data represented by boxplots
ggplot(data = dlt_wide) +
  aes(x = time,
      y = site_diff,
      group = time) +
  geom_boxplot(width = 2) +
```

```
  geom_point(position = 'jitter') +
  geom_hline(yintercept = 0) +
  labs(title = 'Grouped data: dynamic light touch rating difference between condition  N = 26',
       y = 'Dynamic light touch ratings of condition A minus rating of condition B',
       x = 'Time (minutes)')
```

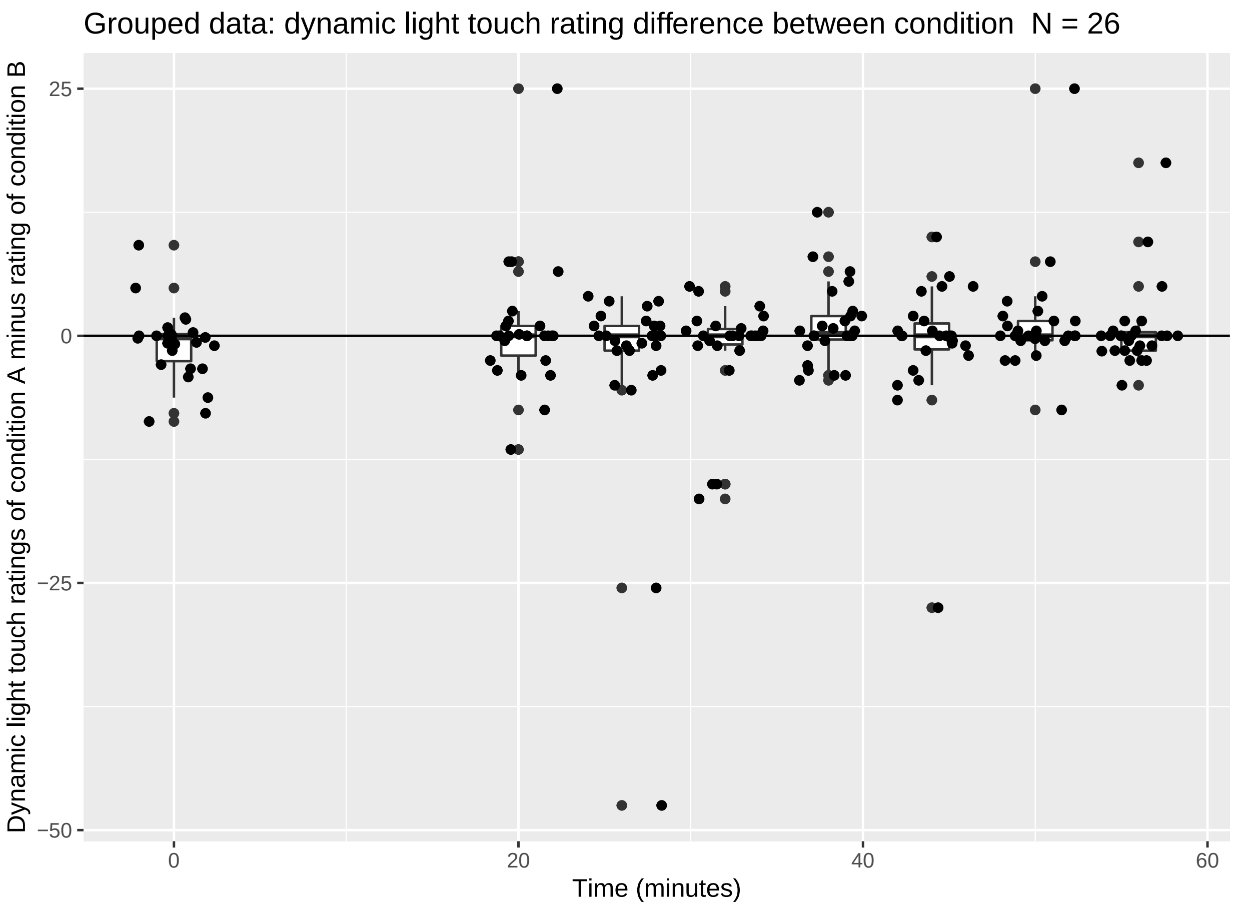

Electrical stimulation

We are interested in determining the magnitude of electrical stimulation on each arm, and the comparison between arms.

```
estim <- master_data %>%
  select(group,
         id,
         time,
         phase,
         site,
         modality,
         rating) %>%
  filter(modality == 'VFF') %>%
  filter(phase != 'orientation') %>%
```

```r
  mutate(time = if_else(phase == 'baseline', time*-1L, time))

estim %<>% mutate(condition = case_when(
  group == '1' & site == 'right' ~ 'A',
  group == '1' & site == 'left' ~ 'B',
  group == '2' & site == 'right' ~ 'B',
  group == '2' & site == 'left' ~ 'A')) %>%
  select(-group,
         -site)
estim$rating <- as.numeric(estim$rating)

estim_bl <- estim %>% filter(phase == 'baseline') %>%
  group_by(id, condition) %>%
  summarise(rating = mean(rating)) %>%
  ungroup() %>%
  mutate(time = 0) %>% ungroup()

estim_fu <- estim %>% filter(phase != 'baseline') %>% ungroup () %>%
  select(-phase,
         -modality)

estim <- rbind(estim_bl, estim_fu)

estim_wide <- estim %>%
  group_by(id) %>%
  spread(key = condition,
         value = rating)

#Determining the mean (SD) estim rating per condition
mean(estim_wide$A, na.rm = TRUE)
```

```
## [1] -29.26619
```

```r
sd(estim_wide$A, na.rm = TRUE)
```

```
## [1] 20.4084
```

```r
mean(estim_wide$B, na.rm = TRUE)
```

```
## [1] -29.58072
```

```r
sd(estim_wide$B, na.rm = TRUE)
```

```
## [1] 20.78741
```

```r
# For interest, plot the data

ggplot(data = estim) +
  aes(x = time,
      y = rating,
```

```r
      group = condition,
      colour = condition) +
  facet_wrap(~ id) +
  geom_point() +
  geom_line() +
  guides(colour = guide_legend("Condition"), size = guide_legend("Condition")) +
  labs(title = 'Individual electrical stimulation ratings by condition, over time',
       y = 'Rating',
       x = 'Time (minutes)')
```

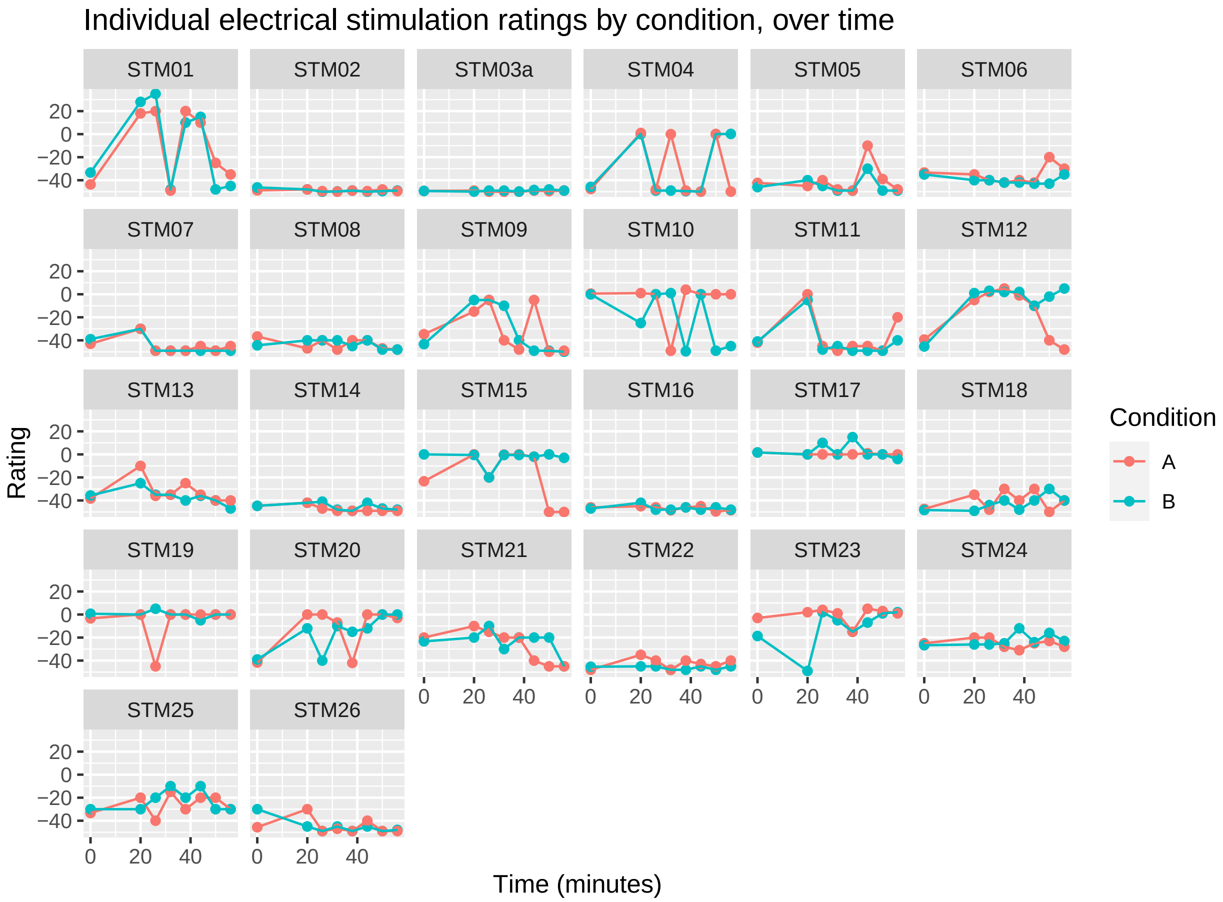


```r
# All participants, group data represented by boxplots
ggplot(data = estim) +
  aes(x = time,
      y = rating,
      group = interaction(condition, time),
      colour = condition) +
  geom_boxplot(width = 4) +
  geom_jitter(width = 0.2) + # Same solution as before
  scale_colour_manual(values = c("#009E73", "#D55E00")) +
  guides(colour = guide_legend("Condition"), size = guide_legend("Condition")) +
  labs(title = 'Group data: Electrical stimulation ratings by condition, over time N = 26',
       y = 'SPARS rating',
       x = 'Time (minutes)')
```

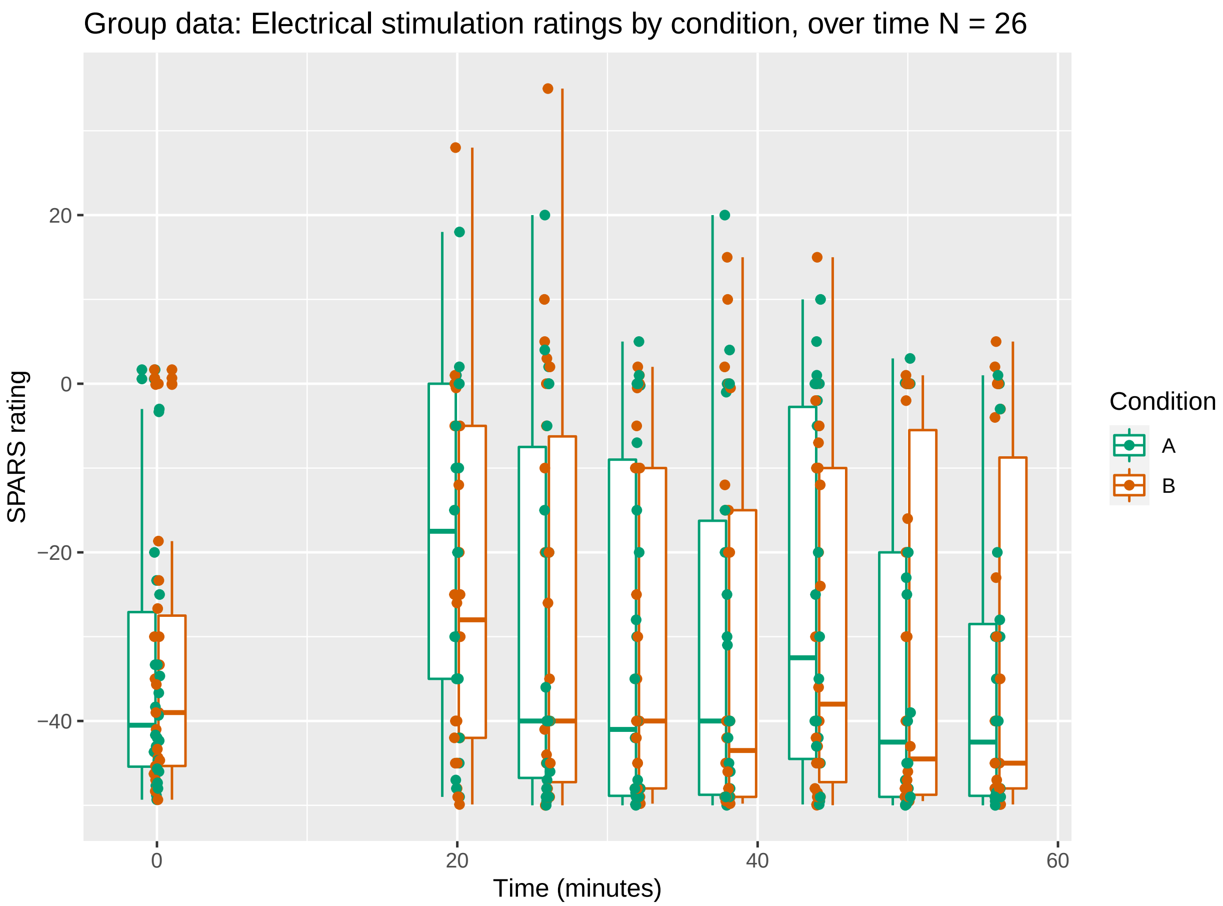


```
# Calculate diff between conditions for group boxplots

estim_wide <- estim %>%
  group_by(id,
           time) %>%
  spread(key = condition,
         value = rating) %>%
  mutate(site_diff = A-B)

# All participants, group data represented by boxplots
ggplot(data = estim_wide) +
  aes(x = time,
      y = site_diff,
      group = time) +
  geom_boxplot(width = 2) +
  geom_point(position = 'jitter') +
  geom_hline(yintercept = 0) +
  labs(title = 'Grouped data: Electrical stimulation ratings difference between conditions N = 26',
       y = 'Electrical stimulation ratings of condition A minus rating of condition B',
       x = 'Time (minutes)')
```

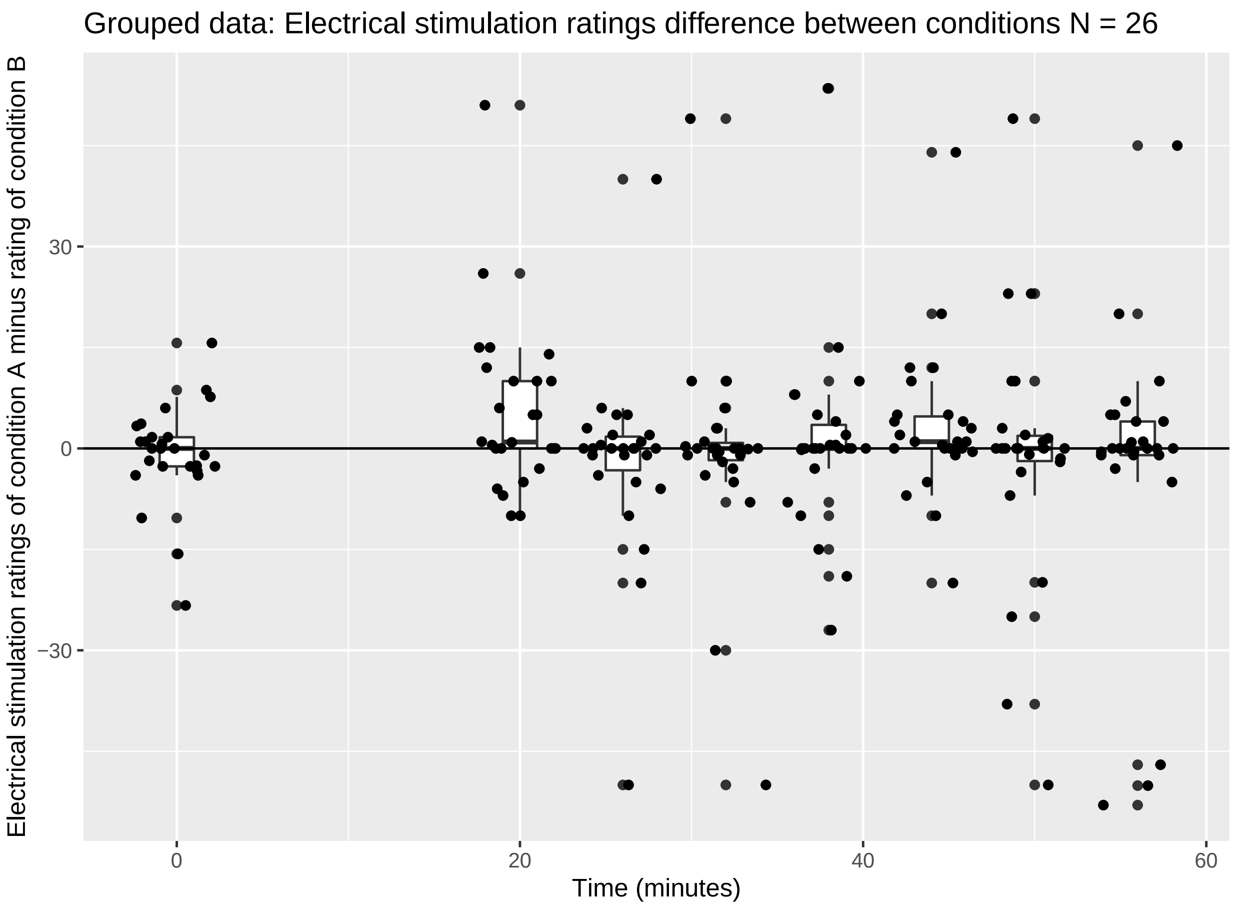

Session information

```r
sessionInfo()
```

```
## R version 4.1.1 (2021-08-10)
## Platform: x86_64-w64-mingw32/x64 (64-bit)
## Running under: Windows 10 x64 (build 19044)
##
## Matrix products: default
##
## locale:
## [1] LC_COLLATE=English_South Africa.1252  LC_CTYPE=English_South Africa.1252
## [3] LC_MONETARY=English_South Africa.1252 LC_NUMERIC=C
## [5] LC_TIME=English_South Africa.1252
##
## attached base packages:
## [1] grid      stats     graphics  grDevices utils     datasets  methods
## [8] base
##
## other attached packages:
##  [1] REdaS_0.9.3     lmerTest_3.1-3  yaml_2.2.2      here_1.0.1
```

```
##  [5] gridExtra_2.3   lme4_1.1-27.1   Matrix_1.3-4    readxl_1.3.1
##  [9] magrittr_2.0.1  forcats_0.5.1   stringr_1.4.0   dplyr_1.0.7
## [13] purrr_0.3.4     readr_2.1.1     tidyr_1.1.4     tibble_3.1.5
## [17] ggplot2_3.3.5   tidyverse_1.3.1
##
## loaded via a namespace (and not attached):
##  [1] httr_1.4.2          bit64_4.0.5         vroom_1.5.7
##  [4] jsonlite_1.7.3      splines_4.1.1       modelr_0.1.8
##  [7] assertthat_0.2.1    cellranger_1.1.0    numDeriv_2016.8-1.1
## [10] pillar_1.7.0        backports_1.4.1     lattice_0.20-45
## [13] glue_1.4.2          digest_0.6.29       rvest_1.0.2
## [16] minqa_1.2.4         colorspace_2.0-2    htmltools_0.5.2
## [19] pkgconfig_2.0.3     broom_0.7.12        haven_2.4.3
## [22] scales_1.1.1        tzdb_0.2.0          mgcv_1.8-38
## [25] farver_2.1.0        generics_0.1.2      ellipsis_0.3.2
## [28] withr_2.4.3         cli_3.0.1           crayon_1.4.2
## [31] evaluate_0.14       fs_1.5.2            fansi_0.5.0
## [34] nlme_3.1-155        MASS_7.3-55         xml2_1.3.3
## [37] tools_4.1.1         hms_1.1.1           lifecycle_1.0.1
## [40] munsell_0.5.0       reprex_2.0.1        compiler_4.1.1
## [43] rlang_0.4.11        nloptr_1.2.2.3      rstudioapi_0.13
## [46] labeling_0.4.2      rmarkdown_2.11      boot_1.3-28
## [49] gtable_0.3.0        DBI_1.1.2           R6_2.5.1
## [52] lubridate_1.8.0     knitr_1.37          fastmap_1.1.0
## [55] bit_4.0.4           utf8_1.2.2          rprojroot_2.0.2
## [58] stringi_1.7.6       parallel_4.1.1      Rcpp_1.0.8
## [61] vctrs_0.3.8         dbplyr_2.1.1        tidyselect_1.1.1
## [64] xfun_0.29
```
