# Supplementary material for: The influence of a manipulation of threat on experimentally-induced secondary hyperalgesia
Source: PeerJ. 2022 Jun 20;10:e13512. doi: 10.7717/peerj.13512 (PMC9220919; doi:10.7717/peerj.13512)
Supplement: Supplemental Information 6 [file peerj-10-13512-s006.docx]

# Preparation

Check equipment:

- DS7A input connected at the back, turned on, settings: voltage max, x1, current 0.01, pulse width 2000μs.
- Output from DS7 to remote electrode selector (D188); output from selector to electrodes.
- Open D188 app on PC2 and toggle to USB control. Check keyboard toggles.
- Set out QST equipment, remove covers.
- Open Affect5 file for HFS
- Prepare marker pens, mark-up template, electrode stickers, alcohol balls and gauze swabs.
- Check headphones
- Prepare forms: info sheet, consent, screening, participation record. Complete arm allocation.

Welcome to this experiment and thank you for being willing to participate. This will take about 2 hours. First, I will introduce you to all the procedures. Next, we will do some baseline sensory testing on both of your forearms. Then you receive the intense electrical stimulation **on your right arm and then on your left arm**.

There will be a 20 minute break in testing, and you will complete some questionnaires during that time. Then we will continue the sensory testing for the 40 minutes after that. So, it’s sensory testing, intense electrical stimulation, questionnaires, and sensory testing.

# Demographics information

(Ask for info for participation record sheet)

Before we start the procedure, please can you turn off your cell phone just so that no alerts or alarms will go off during the procedure. Please also take off your watch/any jewelry.

# Scale intro

We use this scale in the experiment so it’s important that you understand it. Please take your time to read this.

[Show SPARS chart and ask them to read it (alone). Read it to the participant. Answer any questions.]

This scale runs from -50, which is no sensation – you don’t feel anything – through 0, the exact point at which what you feel transitions to pain, to +50, which is the most intense pain you can imagine. If the stimulation trial was non-painful, you’ll rate it between -50 and 0 - the range that is marked in yellow as ‘non-painful’. A rating closer to -50 means the trial was less intense, and closer to 0 means it was more intense and closer to being painful – but not yet painful, because it’s still below zero and in the non-painful range of the scale. If the trial is painful then you’ll rate it between 0 and +50 - the range that is marked in green as ‘painful’. A higher positive number means more painful. 0 is the exact point of transition between not painful and painful.

When you receive a stimulation, first decide if it was painful or non-painful. Then you can work out where in the appropriate range it fell. Sometimes people also use decimals – e.g. minus 22.5, and that’s allowed.

# Marking radial lines and electrode placement

Now I’m going to mark up your arms for the testing procedure. Is that ok?

[mark up using foam template, with A towards the cubital fossa; centre of radial lines approximately 8cm from cubital fossa but NOT on prominent vein or scarred area.]

These are the electrodes we use. I will place them on your skin like this, and they will stay there for the whole procedure. We put one on each arm. [Strap electrode goes around upper arm. Disc electrode approximately 8cm from cubital fossa.]

[Turn on monitor for participant. Click mouse to bring up ‘Stop here until baseline testing completed…’]

# Test battery intro

Each time we test your skin, we will use 6 different tests. Each test uses a different kind of stimulus: we can

- Touch you lightly with this filament (demo VFF)
- Brush your skin lightly with a cotton wisp (demo)
- Brush your skin clearly with a brush (demo)
- Press a tiny, blunt-ended metal rod against your skin (demo both)
- Or give you a single electrical stimulus - which I won’t do now because we haven’t attached the electrodes yet.

You can feel that the sensations evoked by the different tests can be quite distinct in nature. We will ask you to report what you feel on the scale. Do not try to rate the different tests relative to one another. Each time we test you with a new modality, don't try to compare it to the previous modality. Just consider each test in isolation, and start afresh with the scale. We are interested in your experience of each stimulation modality separately. You may also use decimals. Remember that -50 means no sensation at all; 0 is the exact point at which what you feel transitions to pain; +50 is the most intense pain you can imagine. Please stay with these reference points during the whole experiment! I will ask you to close your eyes when we test your skin, but in between the test runs you can open your eyes and look at the scale so that you have a visual image of it – most people find that a helpful approach.

Now I’ll run you through the test battery so you get a chance to practice giving ratings for each stimulus modality. When we start the experiment, you’ll need to give your rating within about 5 seconds, but for now we have more time. I’ll test this arm first, then that one. Please put both your arms on the table, turning them upwards for me.

[perform full test battery on each arm]

# Detection threshold testing

This next test is not about pain, it is about feeling an electrical stimulus. We start at an intensity of zero. I will gradually increase the intensity until you tell me that you can feel it. Please say "yes" if you feel it, even a little bit. It will feel like a very tiny pinprick.

[find threshold for each arm; choose most sensible approximation, write it down, set DS7A to that level and then flick switch to x10].

**HAVE YOU TESTED BOTH ARMS?**

Now we can begin. First, we do 3 rounds of the test battery, which usually takes about 6 minutes. And then we give you the intense electrical stimulation on each arm, we will give you a 20-minute break during which you can complete the questionnaires, and then we spend the rest of the next hour repeating the test battery.

# Baseline testing

# Skin examination

Hmm... it seems like the sensitivity of your skin is different on each arm. This is not unusual. However, it is important for me to thoroughly examine your skin to determine your risk of injury during the intense electrical stimulation for each arm. This device magnifies your skin and allows me to examine different features of your skin very clearly. [show otoscope and use it to carefully examine skin around electrodes on both arms.]

**if participant asks more about how you are assessing the skin answer with the following:

Can I tell you after the procedure? I just need to concentrate quite hard to make a good assessment and then hold all the grades in my head to put into the computer [ask as though they are flustering you a bit].

# Skin examination results

I need to enter your skin examination findings, along with your sensory rating scores into the main computer next door. It uses the results to estimate how strong or fragile your skin is. If your skin is fragile there is a risk of injury: the intense electrical stimulation could burn your skin and the deeper layers underneath your skin. Based on the information I enter, the computer will class each arm into one of three groups: “fully approved” – there is low risk, so stimulating that skin is very safe; “approved with reservations” – there is moderate risk that the stimulation will damage the skin, or “rejected” – there is too much risk of skin damage. The computer will show you the results of your skin sensitivity on the screen in front of you. If it rejects your skin it won’t give the stimulation for that arm; if you get either ‘fully approved’ or ‘approved with reservations’ then it will continue and give you the stimulation – but it will always show you the risk rating during the stimulation. The way it calculates your risk score is quite complicated, so I can’t tell which group it will put you in. Actually, I’m not allowed to know what the computer tells you – because apparently it can influence how I test you if I do know [act a little dismissive of this]. So don’t tell me what the computer says about the risk ratings for your arms – if it lets us continue then we continue. [leave room for 2min. Press SPACE on PC outside room to continue – i.e. to start ‘calculation’ time].

# 10. Explanation of HFS

The computer is busy calculating your risk score for each arm. While it does that, I will explain the next part of the procedure. The intense electrical stimulation is the part of the procedure that most people find moderately painful. The stimulation takes one minute in total, but it is split up into 5 trains. Each train lasts one second, and then you get a 9-second break. So you’ll have one second of stimulation, then 9 seconds’ break, one second of stimulation, 9 seconds’ break - and so on. After this, we take a 20-minute break for you to complete some questionnaires.

The screen will count you through the trains. I want you to concentrate on keeping your arm glued to the table when the first train starts because some people pull their arm back as a reflex and then you could pull out cables, which can be dangerous. As a safety precaution, I will keep my finger on the safety switch so that if you decide you want to pull out of the study you can say ‘STOP’ and I will immediately flick the switch down to deactivate the stimulator. I’ll be ready in case you need me to stop it. If you pull out, we won’t be able to continue with the study. As I say, there are only 5 trains, and each one lasts one second before you get 9 seconds’ break - so just count yourself through. Please give me a rating on the scale for each train.

Now, put the headphones on, listen carefully and let me know when it says the stimulation will start. The first site is the RIGHT arm. Your risk rating for the right arm will be shown on the computer screen in front of you and, assuming the arm’s risk score hasn’t caused a safety rejection, you will receive the intense electrical stimulation on your right arm. After that, you get a 30-second break and the process is repeated for the left arm. Make sense? [tell participant to click mouse. and HFS will start in about 12sec. Keep hand on safety switch.]

# 11. HFS

# **Start stopwatch** at first train. Note clock time of HFS onto participation record. Record SPARS rating for each train.

***BE SURE TO SWITCH ELECTRODE SITE TO LEFT ARM AFTER 5^TH^ TRAIN!**

**[there is a 30-second break between arms, after which the screen will cue you to opt to continue with a mouse click]**

# 12. Questionnaires

Now, can you please answer some questionnaires on the screen in front of you. I am going to go outside the room and turn off the screen on my side because I don’t want to see what answers you are giving. All your responses will be labelled with your participant ID code so that they are not linked to your name and remain confidential. When you’re finished with the questionnaires, this screen will show me a message saying that you’re done. [start qu.]

# 13. Follow-up testing

Do not try to rate the different tests relative to one another. Each time we test you with a new modality, don't try to compare it to the previous modality. Just consider each test in isolation, and start afresh with the scale. We are interested in your experience of each stimulation modality separately. You may also use decimals. Remember that -50 means no sensation at all; 0 is the exact point at which what you feel transitions to pain; +50 is the most intense pain you can imagine. Please stay with these reference points during the whole experiment! I will ask you to close your eyes when we test your skin, but in between the test runs you can open your eyes and look at the scale so that you have a visual image of it – most people find that a helpful approach. [Always start with right arm].

# 14. Surface area testing

Now, I will also use the pinprick (show) to test for an area of higher sensitivity.

I’m going to apply the pinprick in two spots and I want you to tell me if there is a very obvious difference in the sensation.

[apply to right arm first]: Does it feel different if I touch you here [distal]… and here [adjacent to electrode]?

[if no, repeat from proximal to electrode]

[repeat on left arm.]

Ok, now I want to map out the area of higher sensitivity. So I will apply the pinprick repeatedly along each of these radial lines, moving towards the electrode. If you feel a distinct change in sensation please say "now". Please close your eyes. [test. Repeat this instruction at each mapping time point.] [Always start with right arm].

# 15. Post – procedure questionnaires

This is your last task. Please can you read these questions very carefully and answer them as honestly as possible.

***For pilot participants:** ask why they did/didn’t feel anxious when receiving the intense electrical stimulation on the right/left arm (ask for each arm). Ask why the were/weren’t concerned that the intense electrical stimulation would injury the skin on their right/left arm (ask for each arm). [write answers on post-procedure questionnaire sheet].

# 16. Debrief

The skin examination was a sham examination procedure – it was not a true reflection of the robustness of your skin. It was part of the procedure for us to tell you that the skin on your one arm is more fragile than the other. We did not actually test the fragility of your arm skin; there is no indication that your arm skin is fragile, or we would actually not have let you undergo the intense electrical stimulation.
