# Supplementary material for: The influence of a manipulation of threat on experimentally-induced secondary hyperalgesia
Source: PeerJ. 2022 Jun 20;10:e13512. doi: 10.7717/peerj.13512 (PMC9220919; doi:10.7717/peerj.13512)
Supplement: Supplemental Information 7 [file peerj-10-13512-s007.docx]

Supplementary Table 1 Participants' responses to the semi-structured interview explaining why they were/were not anxious and/or fearful of tissue damage during the HFS. ‘Threat site’ denotes the site receiving the HFS under a condition of threat.

| **ID code** | **Threat site** | **Site associated with increased anxiety and/or threat of tissue damage** | **Semi-structured interview response asking participants why they agreed/disagreed to (1) being anxious and (2) threatened about tissue damage during the HFS induction.** | **Reason for increase/no change in anxiety and/or threat of tissue damage** |
| --- | --- | --- | --- | --- |
| STM01 | Left |  | No data^[[1]](#footnote-1)^ |  |
| STM02 | Left | Equal concern | “I was worried that skin would be affected on both arms not really affect by what it said on the computer screen, stimulations to each arm felt equal so [I] was equally concerned.” | Pain intensity during HFS |
| STM03 | Right | Both right and left, but more concerned at the left | Participant selected *‘agree’* to being anxious about the *right* arm “because of what the computer screen said. I was not expecting that type of pain”.  Participant selected *‘strongly agree’* to being anxious about the *left* arm, because the “left arm felt a lot more painful and my fingers moved I so felt anxious”. | Threat manipulation  Pain intensity during HFS |
| STM04 | Left | Right | Participant reported to *‘agree’* to threat relating to the *right* arm because the “right was more painful. My [dominant] right arm is more important to me than my left”. Participant reported that their concern was “not really due to what the computer screen said”. | Hand importance |
| STM05 | Left | Left | Participant selected *‘agree’* to threat relating to the *right* arm because "[the HFS] started with right arm. It was more of a shock, so I was more concerned.”  Participant selected *‘strongly agree’* to threat relating to the *left* arm “based on what the computer said, and it felt more pain on that side”. | Threat manipulation  Pain intensity during HFS |
| STM06 | Right | Equal concern | “Right arm felt more painful than left. However, I was equally anxious because of pain intensity”. | Pain intensity during HFS |
| STM07 | Left | Right | “I wasn't too concerned about skin damage. I trusted enough precautions had been taken.”  Participant selected *‘agree’* to being anxious about their *right* arm, they felt anxious because “of the anticipation”. “Maybe I was a bit more anxious about the right arm.” | Anticipation of HFS  Trust in precautionary measures *reduced* anxiety and threat |
| STM08 | Right | Equal concern | “The anticipation of the HFS made me feel anxious. I wasn't too concerned about the rating on the screen. I trusted it wasn't going to damage the skin.” | Anticipation of HFS  Trust in precautionary measures *reduced* anxiety and threat |
| STM09 | Left |  | Participant selected *‘agree’* to threat relating to the *right* arm because “I felt from the beginning that the left arm was more sensitive”.  Participant selected *‘strong agree’* to being anxious about both their *right and left* arm, they felt anxious because “the anticipation of when the stimulus would happen made me feel really anxious”. | Anticipation of HFS |
| STM10 | Right | Equal concern | “Expecting the pain was going to increase after each stimulation made me concerned that the increase may be causing damage and associated anxiety.” | Anticipation of HFS |
| STM11 | Right | Equal concern | Participant selected *‘agree’* to threat relating to the *right* arm “based on what it said on the screen I felt concerned about damage”.  Participant selected *‘agree’* to being anxious about their *left* arm, they felt anxious because “it was more painful on the left, so I was more anxious”. | Threat manipulation  Pain intensity during HFS |
| STM12 | Right | Equal concern | Participant selected *‘disagree’* to threat relating to both the *right and left* arm because “at the beginning of the study, the informed consent said there shouldn't be any harm or damage. I trusted that”.  Participant selected *‘agree’* to being anxious about both their *right and left* arm because “anticipation of pain, and unexpected pain. I was worried whether the next [HFS train] would be better than one before”. | Anticipation of HFS  Trust in precautionary measures *reduced* anxiety and threat |
| STM13 | Left | Left | “I was concerned about what it said on the screen, which obviously made me anxious”. | Threat manipulation |
| STM14 | Left | Not at all concerned | “I trusted that the researchers wouldn’t put me through anything dangerous. I had forgotten one [arm] was more fragile by the time we started baseline testing after the questionnaires.” | Trust in precautionary measures *reduced* anxiety and threat |
| STM15 | Right | Equal concern | “The intensity and anticipation of stimulation was anxiety provoking”. | Anticipation of HFS |
| STM16 | Right | Equal concern | Participant selected *‘agree’* to threat relating to both the *right and left* arm because “I felt equally concerned that there could be skin damage to both arms, even based on what the computer screen said. It was more just the overall risk that there could be damage”. | Risk of procedure |
| STM17 | Right | Equal concern | Participant selected *‘strongly disagree’* to threat relating to both the *right and left* arm because “the stimulation trains were too short to cause damage”.  Participant selected *‘neutral’* to be anxious about both their *right and left* arm because “waiting for the next stimulation made me feel anxious”. | Skin damage implausible  Anticipation of HFS |
| STM18 | Right | Equal concern | Participant selected *’disagree’* to being anxious about both their *right and left* arm, they felt anxious because “I was more nervous about the anticipation of the stimulus than what was on the screen". | Anticipation of HFS |
| STM19 | Left | Equal concern | Participant selected *‘strongly disagree’* to threat relating to their *left* arm because “of what the computer screen said”.  Participant selected *‘strongly agree’* to being anxious about both their *right and left* arm, they felt anxious because “of anticipation between trains and how intense the pain was”. | Anticipation of HFS |
| STM20 | Right | Right | “I have worked with UCT human ethics before. I know they are strict. I was slightly concerned about the right because of what the screen said but not enough to 'agree' but [the right] felt a bit stranger than left arm. I believed the skin examination, but I was slightly interested as to why you were using an otoscope.” | Pain intensity during HFS  Trust in precautionary measures *reduced* anxiety and threat |
| STM21 | Left | Not at all concerned | Participant selected *‘disagree’* to threat relating to their *left* arm because "I wasn't too concerned. Maybe a small hint of doubt about left arm, but not enough to think it would cause tissue damage”. | Skin damage implausible |
| STM22 | Left | Equal concern | "More the discomfort that made it feel uncomfortable" | Pain intensity during HFS |
| STM23 | Left | Equal concern | “I was equally concerned but not enough to be totally convinced there could be tissue damage. But this did make me feel anxious.” | Threat manipulation |
| STM24 | Right | Equal concern | Participant selected *‘neutral’* to threat relating to both their *right and left* arm because “I trusted the controlled environment, even though the screens said one arm was more fragile”.  Participant selected *‘agree’* to being anxious about both their *right and left* arm, they felt anxious because “I felt generally anxious just because of pain intensity”. | Pain intensity during HFS  Trust in precautionary measures *reduced* anxiety and threat |
| STM25 | Right | Equal concern | Participant selected *‘agree’* to threat relating to their *left* arm because “it felt more painful on the left.  Participant selected *‘strongly disagree’* and ‘*neutral’* to being anxious about their *right and left* arm, respectively, they felt anxious because “I was anxious about the next train. Also [I was anxious about] what the screen said”. | Pain intensity during HFS  Anticipation of HFS  Threat manipulation |
| STM26 | Left | Equal concern | Participant selected *‘strongly disagree’* to threat relating to both their *right and left* arm because “I trusted the controlled environment”.  Participant selected *‘neutral’* to being anxious about both their *right and left* arm, they felt anxious because “of not knowing what to expect, and the anticipation between trains”. | Anticipation of HFS  Trust in precautionary measures *reduced* anxiety and threat |

1. The researchers in this current study only decided to conduct the semi-structured interview after the first participant had been assessed. [↑](#footnote-ref-1)
