# Supplementary material for: The influence of a manipulation of threat on experimentally-induced secondary hyperalgesia
Source: PeerJ. 2022 Jun 20;10:e13512. doi: 10.7717/peerj.13512 (PMC9220919; doi:10.7717/peerj.13512)
Supplement: Supplemental Information 13 [file peerj-10-13512-s013.doc]

**
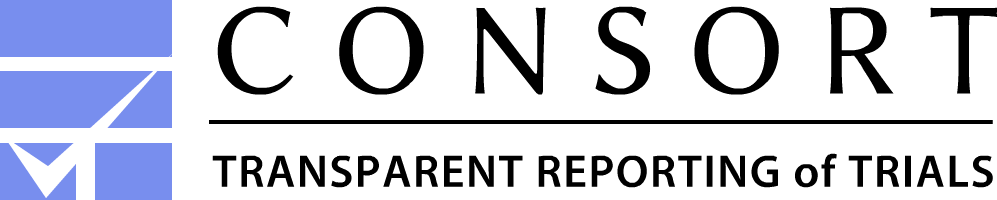
**

**CONSORT 2010 Flow Diagram**

**Allocation**

**Analysis**

**Follow-Up**

**Enrollment**

Assessed for eligibility (n= 40)

Excluded (n=14)

♦  Not meeting inclusion criteria (n=13)

- Tattoo distal to anode (n=5)
- Chronic pain (n=5)
- History of mental illness (n=3)

♦  Declined to participate (n= 0)

♦  Other reasons (n= 1) (participant was unavailable for testing)

Analysed (n= 26)
♦ Excluded from analysis (give reasons) (n= 0)

Lost to follow-up (give reasons) (n=0)

Discontinued intervention (give reasons) (n=0)

Allocated to intervention (n= 26)

♦ Received allocated intervention (n= 26)

♦ Did not receive allocated intervention (give reasons) (n=0)

Lost to follow-up (give reasons) (n=0)

Discontinued intervention (give reasons) (n= 0)

Allocated to intervention (n= 0)

♦ Received allocated intervention (n= 0)

♦ Did not receive allocated intervention (give reasons) (n= 0)

Analysed (n= 0)
♦ Excluded from analysis (give reasons) (n= 0)

Randomized (n=26)
